# Metal-Free C–H Alkyliminylation and Acylation of Alkenes with Secondary Amides

**DOI:** 10.1038/srep28801

**Published:** 2016-06-29

**Authors:** Pei-Qiang Huang, Ying-Hong Huang, Hui Geng, Jian-Liang Ye

**Affiliations:** 1 Department of Chemistry and The Key Laboratory for Chemical Biology of Fujian Province, iChEM (Collaborative Innovation Center of Chemistry for Energy Materials), College of Chemistry and Chemical Engineering, Xiamen University, Xiamen, Fujian 361005, P. R. China

## Abstract

Carbon–carbon bond formation by metal-free cross-coupling of two reactants with low reactivity represents a challenge in organic synthesis. Secondary amides and alkenes are two classes of bench-stable compounds. The low electrophilicity of the former and low nucleophilicity of the latter make the direct coupling of these two partners challenging yet highly desirable. We report herein an unprecedented intermolecular reaction of secondary amides with alkenes to afford *α*,*β*-unsaturated ketimines or enones, which are versatile intermediates for organic synthesis and are prevalent in bioactive compounds and functional materials. Our strategy relies on the chemoselective activation of the secondary amide with trifluoromethanesulfonic anhydride (Tf_2_O)/2-fluoropyridine to generate a highly reactive nitrilium intermediate, which reacts efficiently with alkenes. This metal-free synthesis is characterized by its mild reaction conditions, excellent functional group tolerance and chemoselectivity, allowing the preparation of multi-functionalized compounds without using protecting groups.

Organic chemistry is the chemistry of carbon compounds. Thus carbon–carbon (C–C) bond-forming reactions occupy the central position in organic synthesis. Most of these reactions are polar^1^ by nature and involve the reaction of a nucleophile with an electrophile. The direct reaction of a weak nucleophile with a weak electrophile (Nu_*w*_-El_*w*_) is difficult under conventional reaction conditions. Alkenes are a class of stable and easily available weak π-nucleophiles, which can only undergo transition metal-mediated C–C bond forming reactions, or react with reactive electrophiles such as acyl chlorides (Friedel-Crafts acylation of alkenes)^2^ or *in situ* generated highly electrophilic intermediates, such as nitrilium ions [the extended Bischler–Napieralski (B–N) reaction^3^,^4^,^5^,^6^,^7^], iminium^8^/*N*-acyliminium ions^9^, and acid-activated aldehydes/ketones (Prins reaction^10^). On the other hand, although nitrilium ions are key intermediates in several classical reactions such as Houben-Hoesch^11^, Ritter^12^,^13^, von Braun^13^, Bischler-Napieralski^13^, Beckmann^13^, Schmidt^13^, and Ugi reactions^14^, their participation in synthetically useful intermolecular reactions with alkenes is unknown. Amides are another class of bench-stable compounds with low electrophilicity^15^,^16^ due to the strong resonance between the π^*^ orbital of the carbonyl group and the nitrogen lone pair. It is thus challenging to couple alkenes with amides, especially secondary amides because of the acidic proton on the *N*-atom. As a result, only isolated examples of intramolecular coupling reactions under harsh conditions are known (Fig. 1a). An efficient intermolecular cross-coupling reaction of alkenes with secondary amides remains elusive (Fig. 1b). However, such a transformation would be highly useful considering the widespread use of secondary amides as intermediates in organic synthesis and the requisite conversion of these species into other classes of compounds^16^,^17^ at lower oxidation states^15^,^18^,^19^,^20^,^21^,^22^,^23^,^24^ as well as the versatility of *α*,*β*-unsaturated ketimines (enimines)^25^ and *α*,*β*-unsaturated ketones (enones) in organic synthesis, medicinal chemistry^26^, and molecular switches^27^.

In response to this challenge, we report herein a metal-free intermolecular coupling reaction of secondary amides with alkenes to afford multi-functionalized *α*,*β*-unsaturated ketimines and enones (Fig. 1b). Importantly, with the use of trifluoromethanesulfonic (triflic) anhydride (Tf_2_O) as a powerful yet chemoselective amide-activating reagent, the reactions are conducted under mild conditions and tolerate a host of sensitive functional groups in both the nucleophilic (alkenes) and electrophilic (secondary amides) reaction partners.

## Results and Discussion

### Reaction design

**Figure 2:**
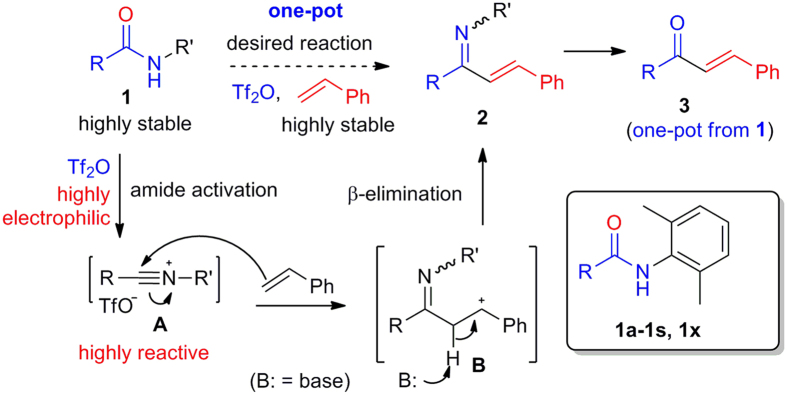
Research design. Strategy for the bimolecular coupling of two highly stable reaction partners: alkenes and secondary amides.

To realize the direct cross-coupling of an alkene with a secondary amide, it is necessary to activate one of the reaction partners. Inspired by the B–N reaction, we opted for the *in situ* activation of the amide group. Considering the low efficiency of the classical amide activators such as P_2_O_5_ and POCl_3_^3^,^4^,^10^, highly electrophilic trifluoromethanesulfonic (triflic) anhydride (Tf_2_O)^28^ was selected for our purpose. Tf_2_O in combination with a base such as 2,6-di-*tert*-butyl-4-methylpyridine (DTBMP)^29^, Hünig base^30^, 2-chloropyridine^31^, 2-fluoropyridine^32^,^33^,^34^,^35^,^36^, 2-iodopyridine^37^, 2,4,6-collidine^38^,^39^,^40^,^41^, and 3-cyanopyridine^42^ had been employed for the activation of amides in various C–C bond-forming reactions. A secondary amide **1**, once treated with Tf_2_O, would generate a highly reactive nitrilium intermediate **A**^32^,^34^,^35^,^36^,^43^ (Fig. 2). The latter could then be captured by an alkene to give an enimine **2** after C–C bond formation and deprotonation. Hydrolysis of the enimine **2** would afford enone **3** in one-pot from amide **1**.

### Optimization of reaction conditions

**Figure 3:**
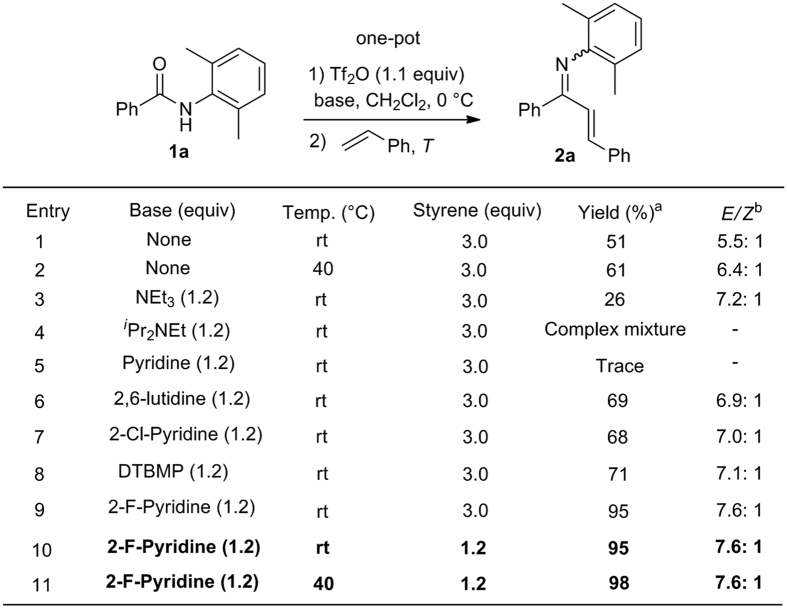
Optimization of the reaction conditions. ^a^Isolated yield. ^b^The *E/Z* ratio of imine was determined by ^1^H NMR. Tf_2_O = trifluoromethanesulfonic anhydride. 2,6-lutidine = 2,6-dimethylpyridine. DTBMP = 2,6-di-*tert*-butyl-4-methylpyridine.

To avoid possible side reactions such as 1,5-hydride migration reaction^36^, the amides **1** bearing a *N*-2,6-dimethylphenyl group were designed as substrates for the investigation (Fig. 3). At the outset of our studies, base-free amide activation protocol was attempted. To our delight, successive treatment of a solution of secondary amide **1a** (1.0 equiv) in CH_2_Cl_2_ (0.25 M) with Tf_2_O (1.1 equiv) at 0 °C for 10 min and then styrene (3.0 equiv) at room temperature for 2 h produced the desired *α*,*β*-unsaturated ketimine **2a** in 51% yield as a mixture of *E/Z* isomers in a ratio of 5.5:1 (entry 1). The stereochemistry of major geometric isomer was determined as *E* by NOESY technique (cf. Supplementary Figure 53). Note that Tf_2_O failed to promote the B–N cyclization reaction in the absence of a base unless highly electron-rich substrates were used^44^. Encouraged by this result, the effects of base were surveyed. Among the bases screened, 2-F-pyridine was found to be the best (entries 3–9). Under these conditions, the amount of styrene could be reduced to 1.2 equiv without affecting the reaction efficiency (entries 10 and 11). The optimal conditions were thus defined as successive treatment of a solution of secondary amide **1a** (1.0 equiv) and 2-fluoropyridine (1.2 equiv) in CH_2_Cl_2_ (0.25 M) with Tf_2_O (1.1 equiv) at 0 °C for 10 min, and then with styrene (1.2 equiv) at room temperature or 40 °C for 2 h. The reaction mixture was concentrated without work-up and subjected to flash chromatographic purification to give *α,β*-unsaturated ketimine **2a**.

### Substrate scope of the direct C–H alkyliminylation

**Figure 4:**
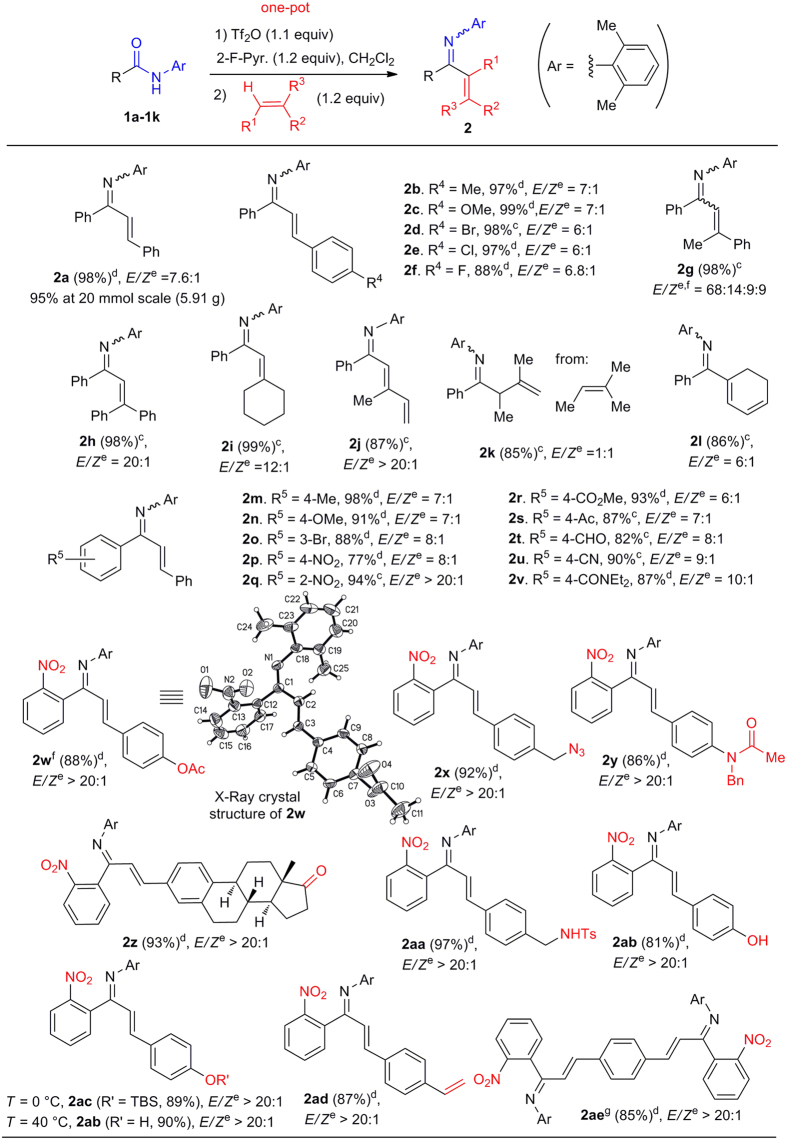
Metal-free direct coupling of *N*-2,6-dimethylbenzamides with alkenes to give α,β-unsaturated ketimines 2. ^a^Reaction conditions: Amide (1.0 equiv), 2-F-Pyr. (1.2 equiv), CH_2_Cl_2_ (0.25 M), then 0 °C, Tf_2_O (1.1 equiv), 10 min. Alkene (1.2 equiv), 2 h. ^b^Isolated yield. ^c^Reaction ran at room temperature (rt). ^d^Reaction ran at 40 °C. ^e^The *E/Z* ratio of imines was determined by ^1^H NMR. ^f^The structure was determined by X-Ray analysis (cf. Supplementary Figure 54). ^g^2.5 equiv of amide **1f** and 1.0 equiv of 1,4-divinylbenzene were used. Ts = 4-toluenesulfonyl, TBS = *tert*-butyldimethylsilyl.

With optimized conditions in hand, the coupling reactions of a series of *N*-(2,6-dimethylphenyl)benzamides **1** with a number of alkenes were investigated (Fig. 4). Styrene bearing electron-donating groups (Me, OMe) and electron-withdrawing halogens (Br, Cl, F) reacted smoothly to give the corresponding enimines in excellent yields (**2b**–**2f**, 88–99% yields), demonstrating superior reactivity compared with reported methods. *α*-Methylstyrene and *α*-phenylstyrene were also competent substrates (**2g**, **2h**). Gratifyingly, the reaction was also compatible with the use of di- and trisubstituted aliphatic alkenes and 1,3-dienes (**2i**–**2l**). The reaction of 2-methyl-2-butene produced non-conjugated *β*,*γ*-unsaturated ketimine **2k**. Further investigation revealed that the reaction was rather insensitive to the electronic properties of the benzamide derivatives and tolerated electron-donating groups such as methyl group (**2m**) and methoxy group (**2n**), as well as the highly electron-withdrawing nitro group (**2p**, **2q**).

The current reaction is characterized by its broad tolerance of sensitive functional groups including bromo (**2o**), nitro (**2p, 2q**), ester (**2r**, **2w**), ketone (**2s, 2z**), aldehyde (**2t**), cyano (**2u**), azido (**2x**), tertiary amide (**2v, 2y**), sulfonamide (**2aa**), phenol (**2ab**) and silyl ether groups (**2ac**), many of which are not compatible with organometallic reagents. The highly functionalized products were all obtained in good to excellent yields, demonstrating great potential for the Tf_2_O-promoted method in the synthesis of complex structures. Interestingly, *p*-vinylstyrene could react selectively at one end giving **2ad** in 87% yield, or at both alkenes leading to **2ae** in 85% yield. Finally, the reaction could be scaled up to 20 mmol-scale without yield loss as demonstrated by the reaction of **1a** with styrene (**2a**, yield: 95%, 5.91 g, Fig. 4).

### Substrates scope of the direct C–H acylation

**Figure 5:**
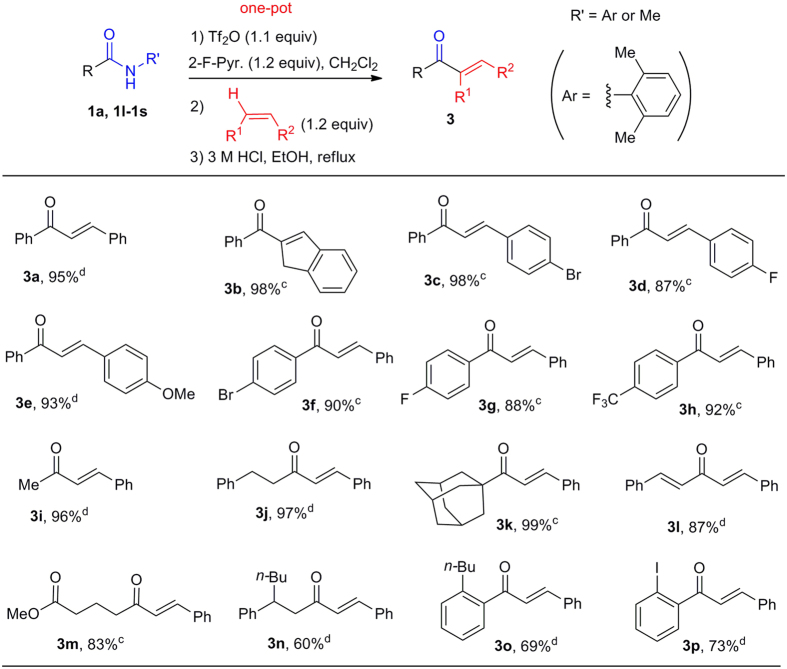
Metal-free direct coupling of benzamide derivatives with alkenes to give enones 3. ^a^Reaction conditions: Amide (1.0 equiv), 2-F-Pyr. (1.2 equiv), CH_2_Cl_2_ (0.25 M), then 0 °C, Tf_2_O (1.1 equiv), 10 min. Alkene (1.2 equiv), 2 h. EtOH and 3 M HCl, reflux. ^b^Isolated yield. ^c^Reaction ran at room temperature (rt). ^d^Reaction ran at 40 °C.

We then turned our attention to the synthesis of enones by *in situ* hydrolysis of the ketimine products. After the Tf_2_O/2-fluoropyridine-mediated dehydracoupling, the reaction mixture was concentrated and heated to reflux in a mixture of ethanol and 3 M HCl (1:1, *v/v*) to afford the desired enones (Fig. 5). Functionalized chalcones **3c**–**3h** were synthesized in good to excellent yields by employing styrenyl alkenes and benzamide derivatives as substrates. Aliphatic and *α*,*β*-unsaturated amides were also excellent substrates (**3i**–**3k**, **3m**). *N*-Alkyl amides are valuable directing group for both classical lithiation-functionalization^45^ and modern C–H functionalization reactions^46^,^47^,^48^,^49^. As a result, the transformation of the functionalized amide products obtained in these reactions into other classes of compounds are imperative. To demonstrate the value of our method in this context, the *N*-methyl amides **1t**^45^, **1u**^48^ and **1v**^49^, which were previously obtained through transition-metal-catalyzed C–H activation, were converted into the corresponding enones **3n**–**3p** in 60–73% yields.

### Synthetic applications

**Figure 6:**
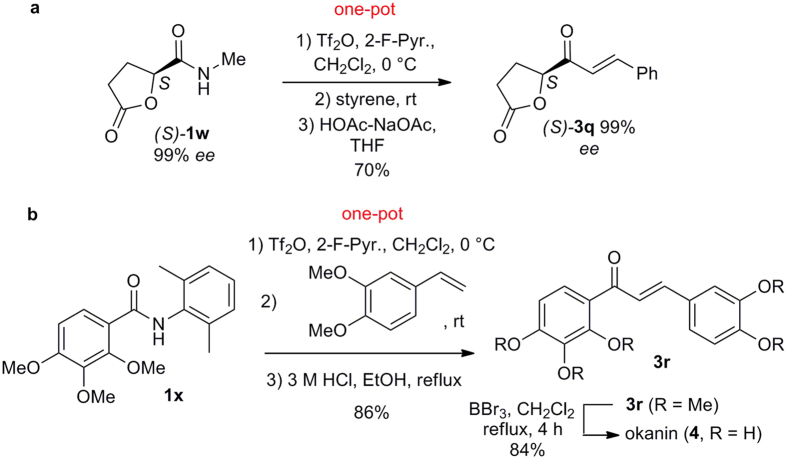
Mildness of the method and Synthetic applications. (**a**) Racemization-free synthesis of a versatile chiral building block **3q**. (**b**) Short synthesis of okanin (**4**).

To demonstrate the synthetic potential of the enone synthesis, the coupling reaction of styrene with (*S*)-*N*-methyl-tetrahydro-5-oxo-2-furaneamide (**1w**), readily available in 99% *ee* from L-glutamic acid^50^, was undertaken (Fig. 6a). To our delight, the desired enone (*S*)-**3q** was obtained in 70% yield without racemization (cf. Supplementary Figure 55). Multi-functionalized lactone-enones like **3q** are versatile building blocks for the synthesis of bioactive natural products^51^.

The synthetic utility was further demonstrated by the synthesis of okanin (**4**) (Fig. 6b), a natural product that has been found in various folk medications used in China and Korea for treating inflammation, malaria, hypertension, diabetes, snake bite and smallpox^52^. The amide **1x**, prepared in one step from commercially available 2,3,4-trimethoxybenzoic acid by amidation using Ye’s coupling reagent^53^ (cf. Supplementary Figure 60), reacted smoothly with 3,4-dimethoxystyrene to afford enone **3r** in 86% yield. Exhaustive demethylation using BBr_3_ furnished okanin (**4**) in 84% yield.

### Mechanistic investigation

**Figure 7:**
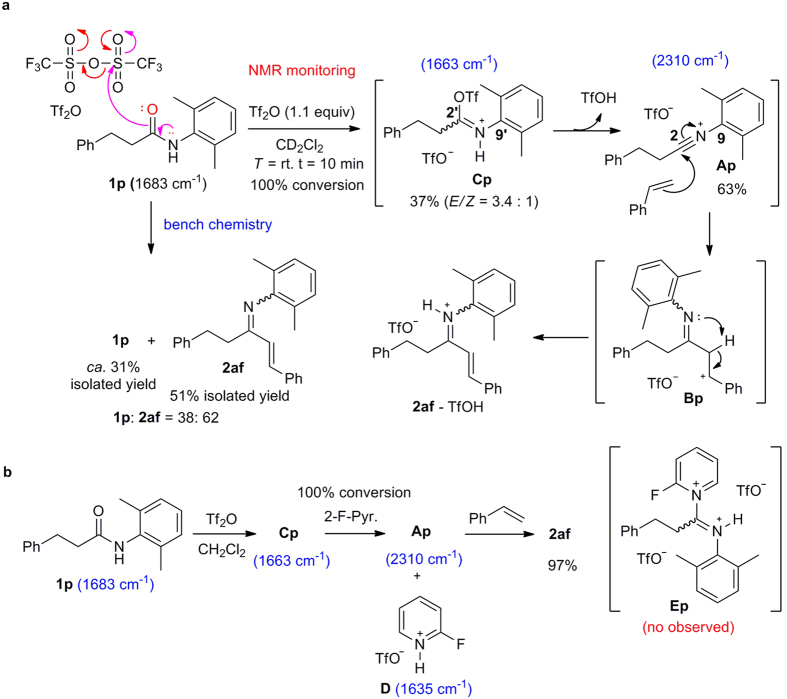
Proposed mechanisms for the direct C–H functionalization of alkenes with secondary amides. (**a**) Reaction in the absence of a base. (**b**) Tf_2_O/2-fluoropyridine-mediated reaction.

**Figure 8:**
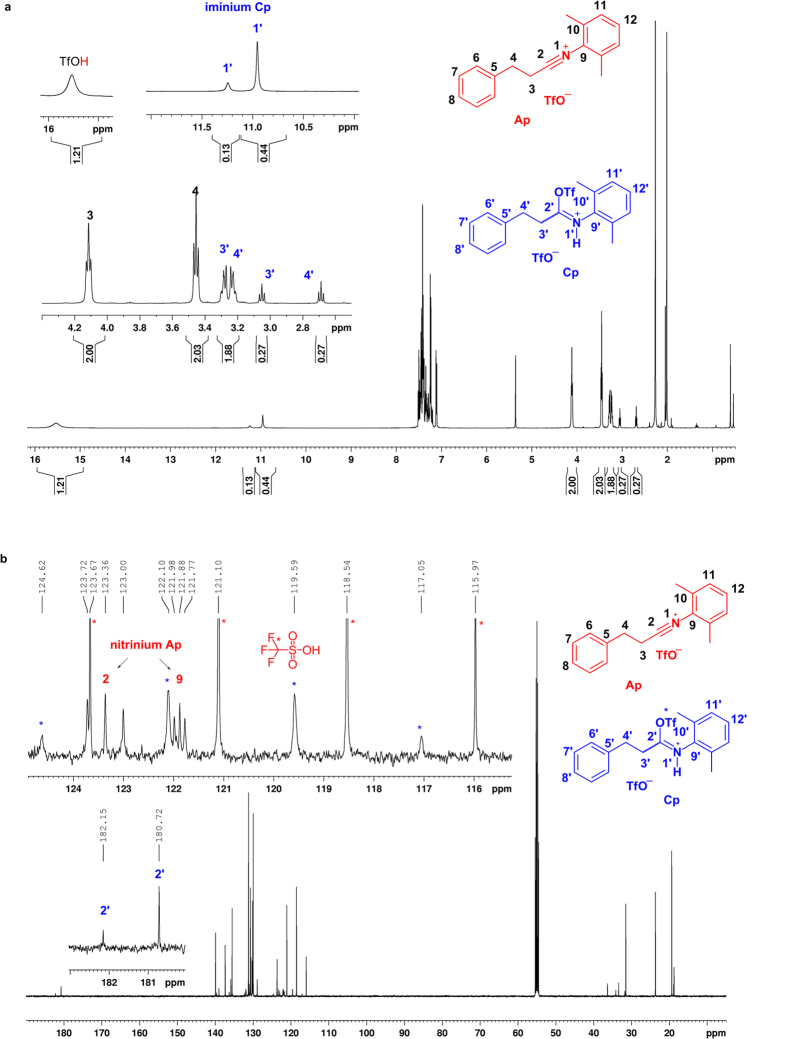
*In situ* NMR monitoring of the base-free direct C–H functionalization of styrene with secondary amide 1p. (**a**) ^1^H NMR spectrum. (**b**) ^13^C NMR spectrum.

To provide some experimental proofs for the presumed intermediacy of a highly electrophilic nitrilium ion, a series of NMR experiments were carried out. Secondary amide **1p** was chosen for the mechanistic studies and base-free amide activation with Tf_2_O was first investigated (Fig. 7a). After addition of Tf_2_O into a solution of amide **1p**, the formation of iminium salt **Cp** (as a 3.4:1 mixture of two geometric isomers) and nitrilium ion **Ap** in a ratio of **Cp**:**Ap** = 37:63 (^1^H NMR, Fig. 8a) was observed. The presence of nitrilium ion **Ap** was manifested by the characteristic triplet resonance and the coupling constant *J*_13C-14N_ of a nitrilium which appeared at *δ*_C2_* = *123.4 (t, *J*_13C-14N_ = 45.6 Hz^54^), as well as the nitrilium *N*-α aromatic carbon at *δ*_C9_* = *121.9 (t, *J*_13C-14N_ = 13.5 Hz^54^) (^13^C NMR, Fig. 8b). Besides, the formation of TfOH was also observed by ^1^H and ^13^C NMR spectra. The same reaction by bench chemistry produced the enimine **2af** in 51% yield along with the recovered starting **1p** in 31% yield (Fig. 7a). Hence, the results obtained from the NMR experiments (**Cp**:**Ap** = 37:63) and those from the bench reaction (**2af**:**1p** = 38:62) suggested that nitrilium ion **Ap** was probably the only competent intermediate that reacted with styrene to produce enimine **2af**. The less reactive iminium salt **Cp** was inert to styrene addition and hydrolyzed upon work-up to regenerate the starting material **1p**. These results also implicated that addition of a base would facilitate the conversion of iminium salt **Cp** to nitrilium ion **Ap**, and thus improve the yield of enimine **2af**. Experimentally, the addition of 1.2 equiv of 2-fluoropyridine boosted the yield of **2af** to 97%. In addition, treating a mixture of amide **1p** and 2-fluoropyridine in CD_2_Cl_2_ with Tf_2_O at 0 °C resulted in quantitative formation of nitrilium ion intermediate **Ap** (Fig. 7b) along with 2-fluoropyridinium salt **D** within 10 min (cf. Supplementary Figure 57 for NMR spectrum). These results confirmed that 2-fluoropyridine promoted the transformation of the iminium salt **Cp** to nitrilium ion intermediate **Ap** (Fig. 7b). Moreover, the *in situ* IR monitoring showed the formation of iminium salt **Cp** (1663 cm^−1^) and nitrilium ion **Ap** (2310 cm^−1^) upon treatment of amide **1p** with Tf_2_O. The former was converted completely into the latter by action of 2-fluoropyridine. A strong absorption of 2-F-pyridinium trifluoromethanesulfonate^32^ (1635 cm^−1^) was observed, while no absorption corresponding to pyridinium ion **Ep** was observed (cf. Supplementary Figure 58 for *in situ* IR spectra).

## Conclusion

In summary, we have developed a general method for the metal-free intermolecular C–H functionalization of alkenes with secondary amides. This method provides a direct and high-yielding access to *α*,*β*-unsaturated ketimines and enones from two classes of readily available and stable starting materials. The one-pot reaction exhibits excellent functional group tolerance for both alkenes and amides allowing convenient and efficient synthesis of a variety of functionalized *α*,*β*-unsaturated ketimines and enones. The present method could find wide applications in organic synthesis especially considering the remarkable chemoselectivity.

## Methods

### General procedure for the direct C–H alkyliminylation and acylation of alkenes with secondary amides to give *α*,*β*-unsaturated ketimines (enimines) 2 and *α*,*β*-unsaturated ketones 3 (enones)

Into a dry 10-mL round-bottom flask equipped with a magnetic stirring bar were added successively a secondary amide (0.5 mmol, 1.0 equiv), 2 mL of anhydrous CH_2_Cl_2_ and 2-fluoropyridine (0.6 mmol, 1.2 equiv) under an argon atmosphere. After being cooled to 0 °C, trifluoromethanesulfonic anhydride (Tf_2_O) (0.55 mmol, 1.1 equiv) was added dropwise *via* a syringe and the reaction was stirred for 10 min. To the resulting mixture, an alkene (0.6 mmol, 1.2 equiv) was added dropwise at 0 °C. The mixture was allowed to warm-up to room temperature (or 40 °C) and stirred for 2 h. The reaction mixture was concentrated under reduced pressure, and the residue was purified by flash column chromatography (FC) on silica gel (pre-neutralized with 2% Et_3_N in *n*-hexane) to afford the desired *α*,*β*-unsaturated ketimine **2**.

Alternately, to the resulting residue were added 5 mL of EtOH and 5 mL of an aqueous solution of HCl (3.0 M). The resulting mixture was heated to reflux until completion of the reaction as monitored by TLC analysis (2–12 h). After being cooled to room temperature, 10 mL of CH_2_Cl_2_ was added, and the mixture extracted with CH_2_Cl_2_ (3 × 10 mL). The combined organic layers were washed with brine, dried over anhydrous Na_2_SO_4_, filtered, and concentrated under reduced pressure. The residue was purified by flash column chromatography on silica gel to afford the desired *α*,*β*-unsaturated ketone **3**.

### Data availability

The X-ray crystallographic coordinates for structures reported in this study have been deposited at the Cambridge Crystallographic Date Center (CCDC), under deposition number CCDC 1438540 (for 2w). These data can be obtained free of charge from The Cambridge Crystallographic Data Centre via www. ccdc.cam.ac.uk/data_request/cif.

## Additional Information

**How to cite this article**: Huang, P.-Q. *et al.* Metal-Free C–H Alkyliminylation and Acylation of Alkenes with Secondary Amides. *Sci. Rep.* **6**, 28801; doi: 10.1038/srep28801 (2016).

## Supplementary Material

Supplementary Information

## Figures and Tables

**Figure 1:**
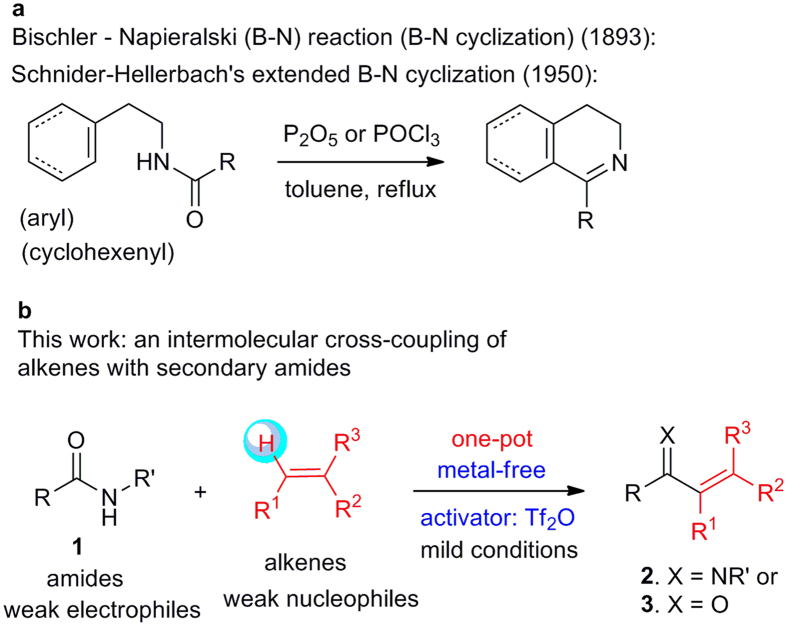
Methods for direct C–H functionalization of alkenes. (**a**) Classical Bischler–Napieralski cyclization (B–N cyclization) and Schnider–Hellerbach’s extended B–N cyclization. (**b**) Our metal-free Tf_2_O-mediated C–H functionalization of alkenes with secondary amides.
