# Supplementary material for: Metal-Free C–H Alkyliminylation and Acylation of Alkenes with Secondary Amides
Source: Sci Rep. 2016 Jun 29;6:28801. doi: 10.1038/srep28801 (PMC4926616; doi:10.1038/srep28801)
Supplement: Supplementary Information [file srep28801-s1.pdf]

Supplementary Information

Metal-Free C–H Alkyliminylation and Acylation of Alkenes with Secondary Amides

Pei-Qiang Huang,* Ying-Hong Huang, Hui Geng, and Jian-Liang Ye

Department of Chemistry and The Key Laboratory for Chemical Biology of Fujian Province, iChEM (Collaborative Innovation Center of Chemistry for Energy Materials), College of Chemistry and Chemical Engineering, Xiamen University, Xiamen, Fujian 361005 (P.R. China)

E-mail: pqhuang@xmu.edu.cn

Supplementary Figures

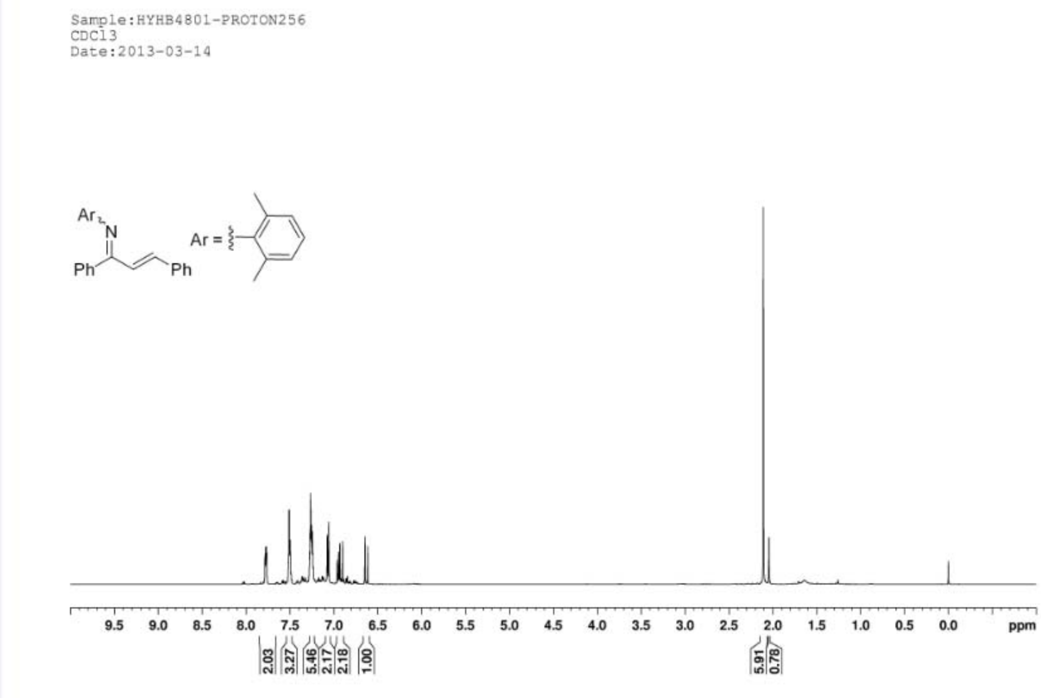

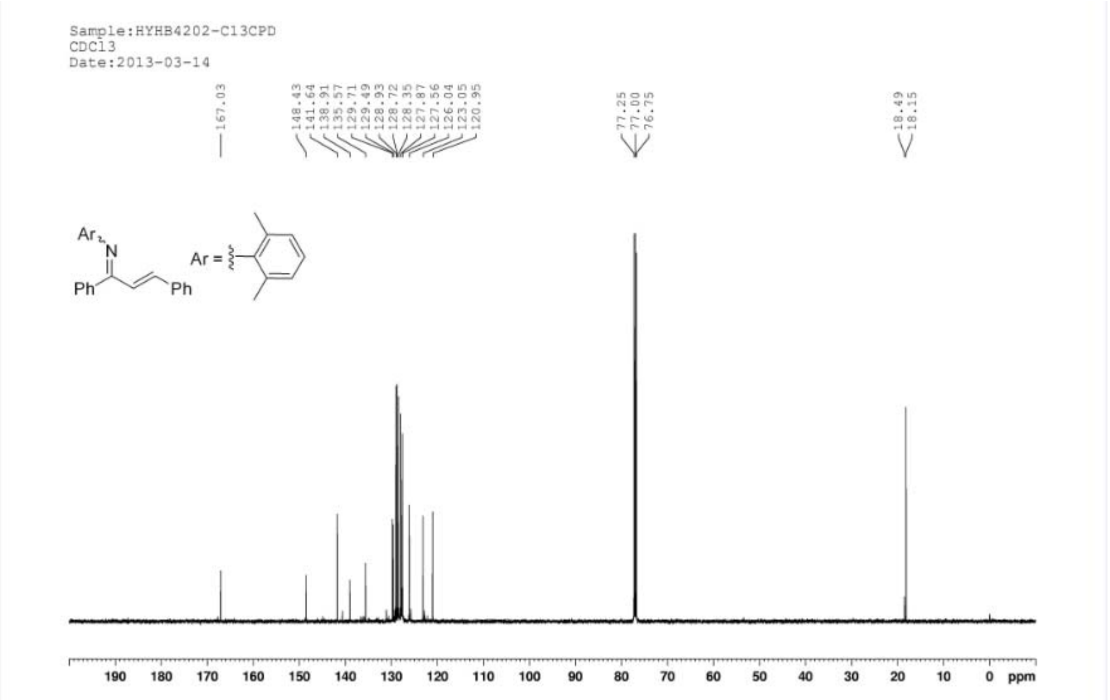

Supplementary Figure 1. ^{1}H NMR (500 MHz) and ^{13}C NMR (125 MHz) spectra of **2a** ($CDCl_3$)

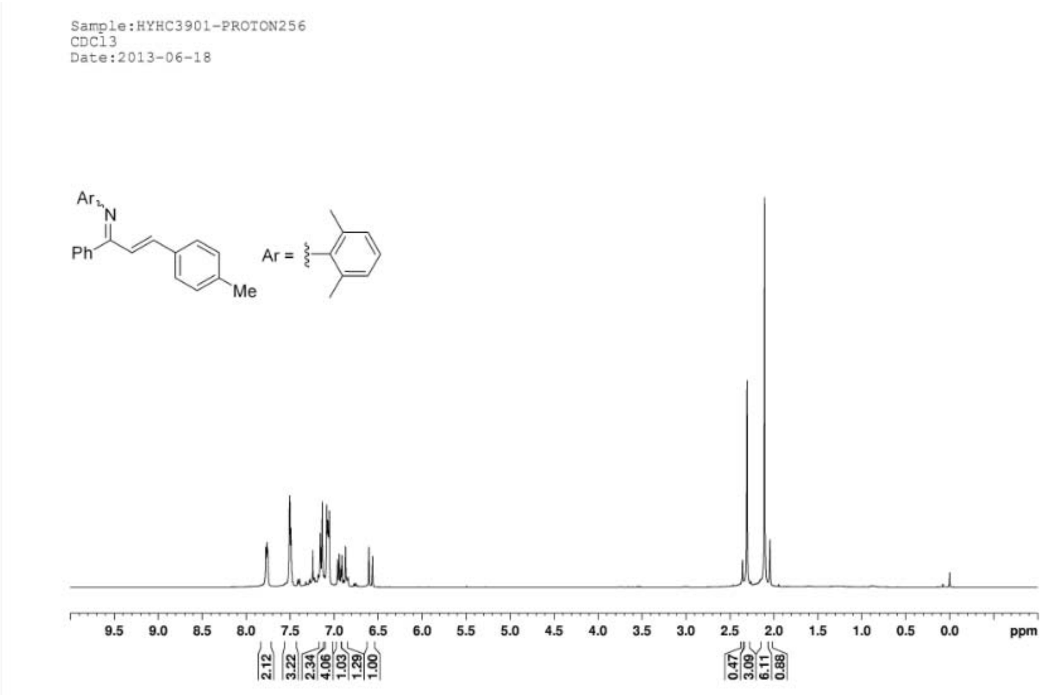

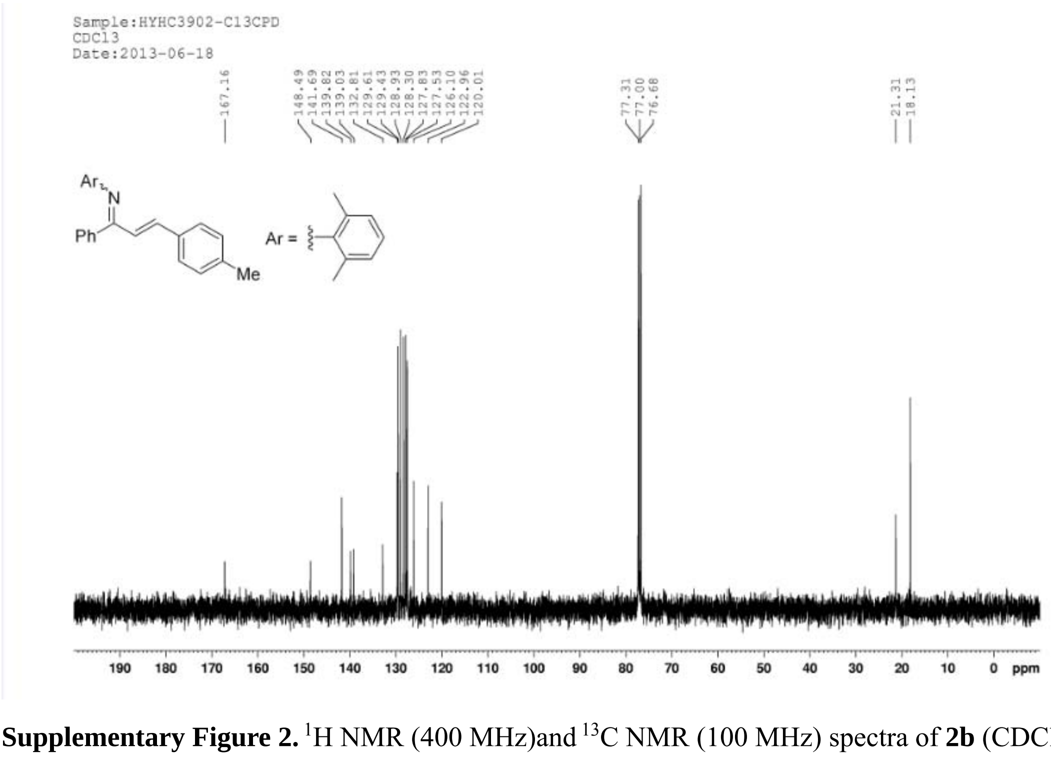

Supplementary Figure 2. ^{1}H NMR (400 MHz)and ^{13}C NMR (100 MHz) spectra of **2b** ($CDCl_3$)

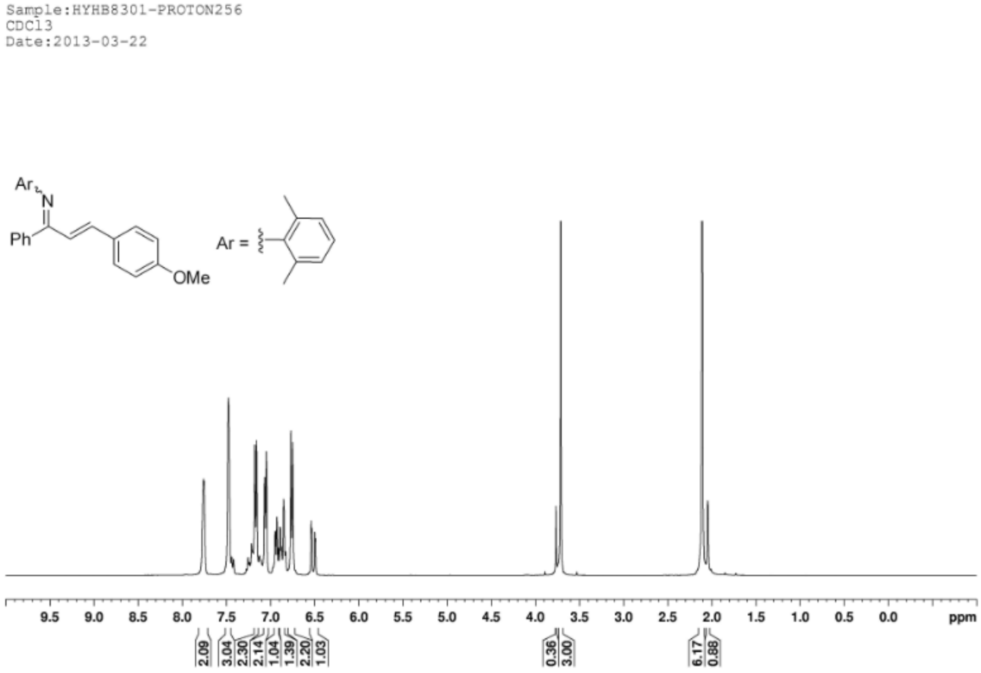

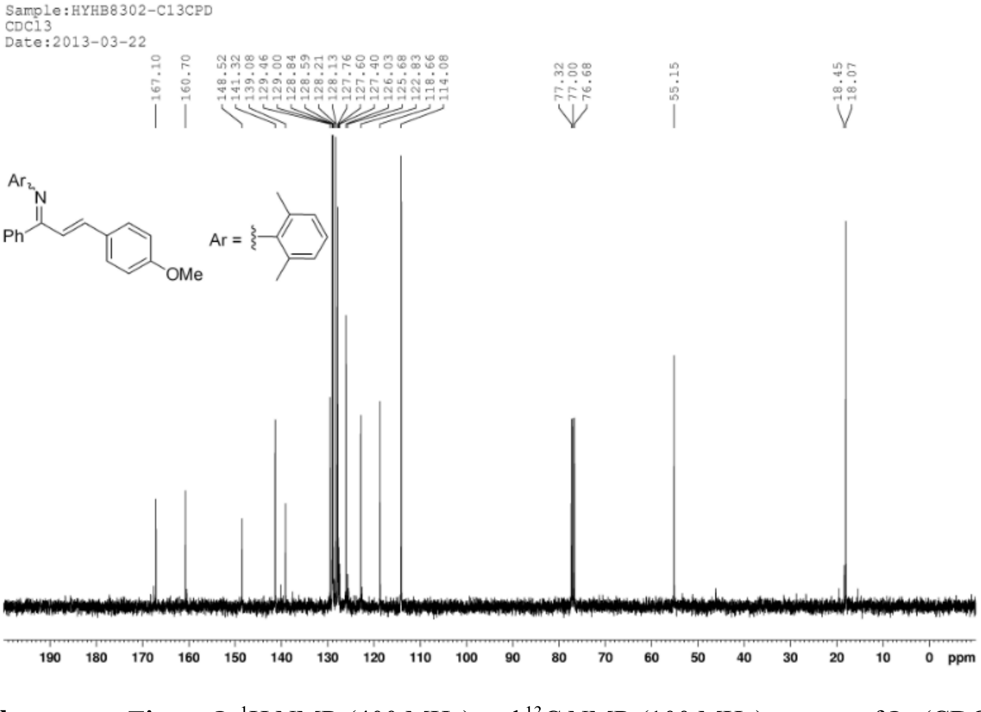

Supplementary Figure 3. ^{1}H NMR (400 MHz) and ^{13}C NMR (100 MHz) spectra of **2c** ($CDCl_3$)

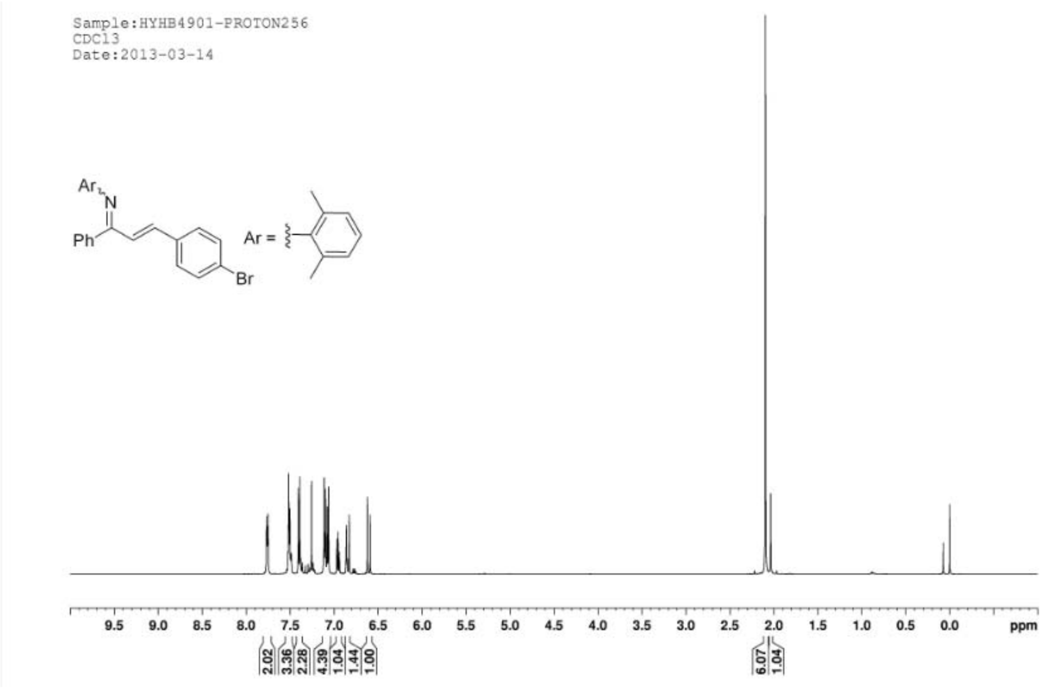

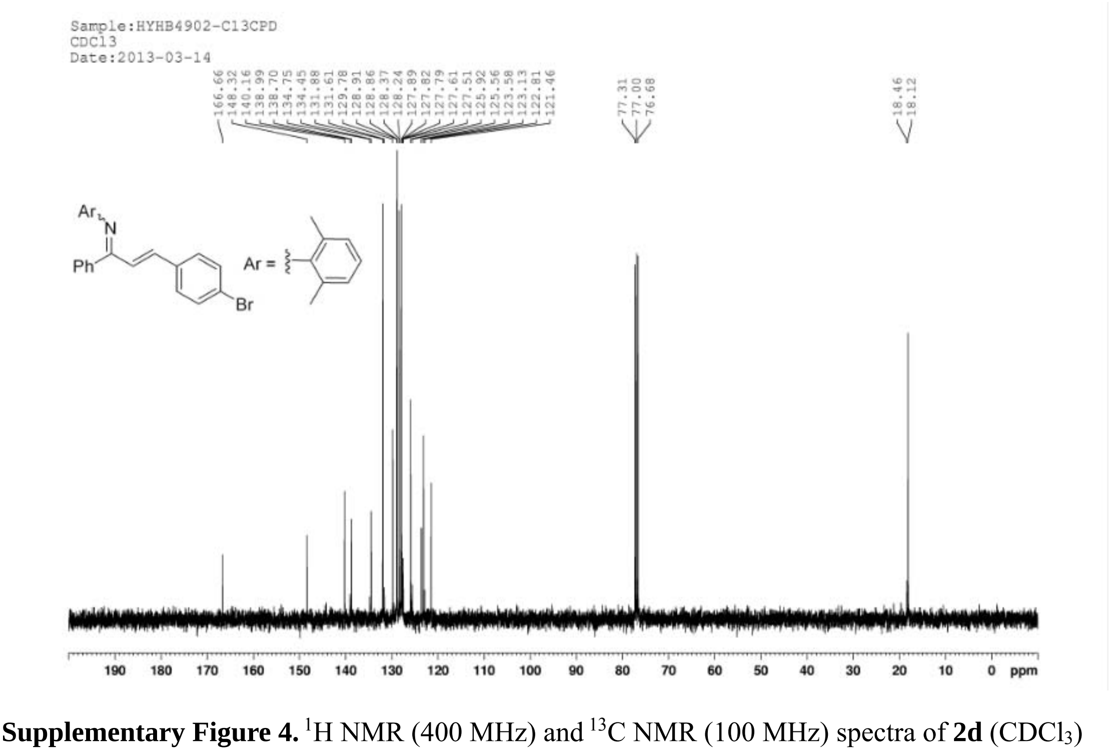

Supplementary Figure 4. ^{1}H NMR (400 MHz) and ^{13}C NMR (100 MHz) spectra of **2d** ($CDCl_3$)

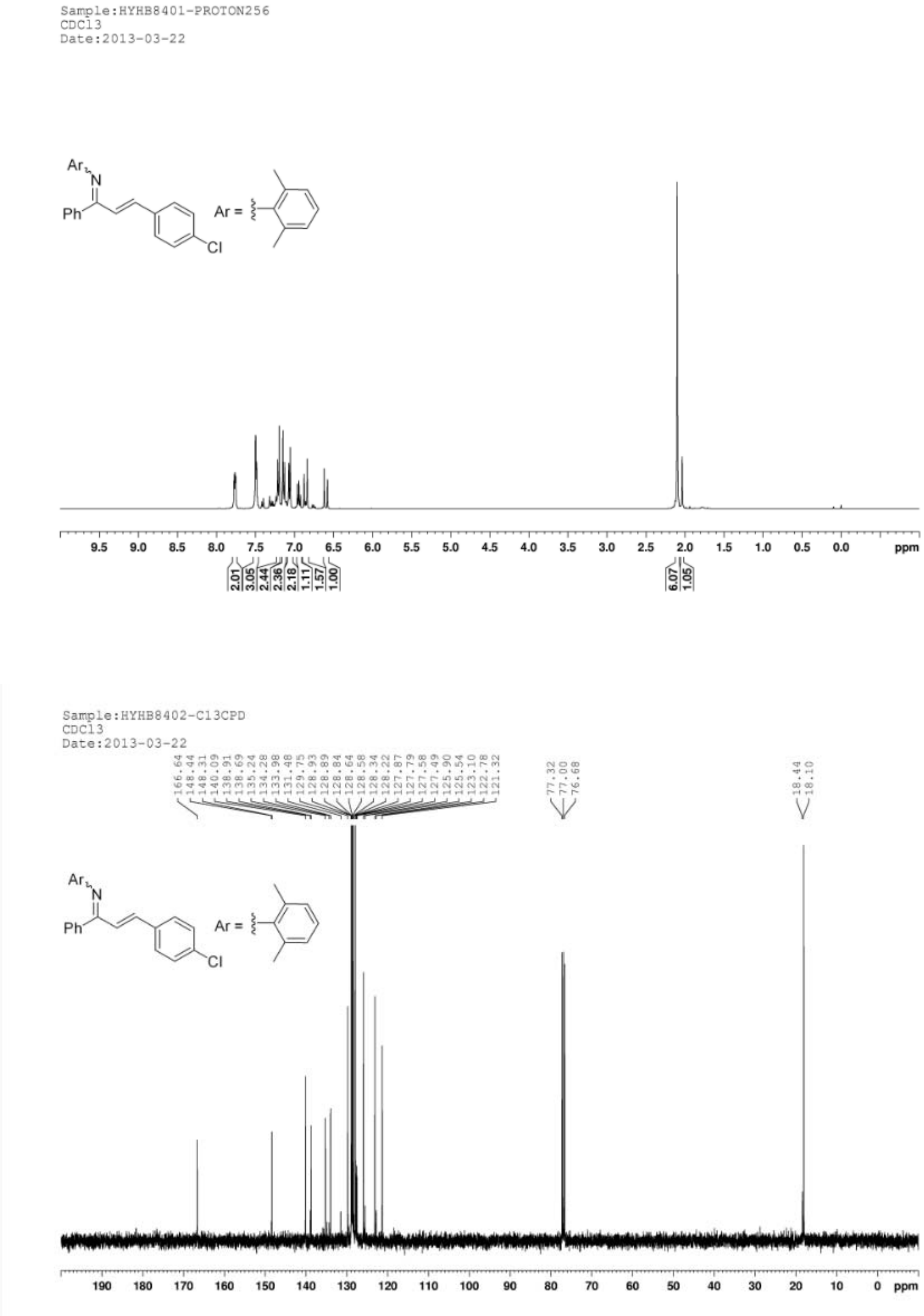

Supplementary Figure 5. ^{1}H NMR (500 MHz) and ^{13}C NMR (125 MHz) spectra of **2e** ($CDCl_3$)

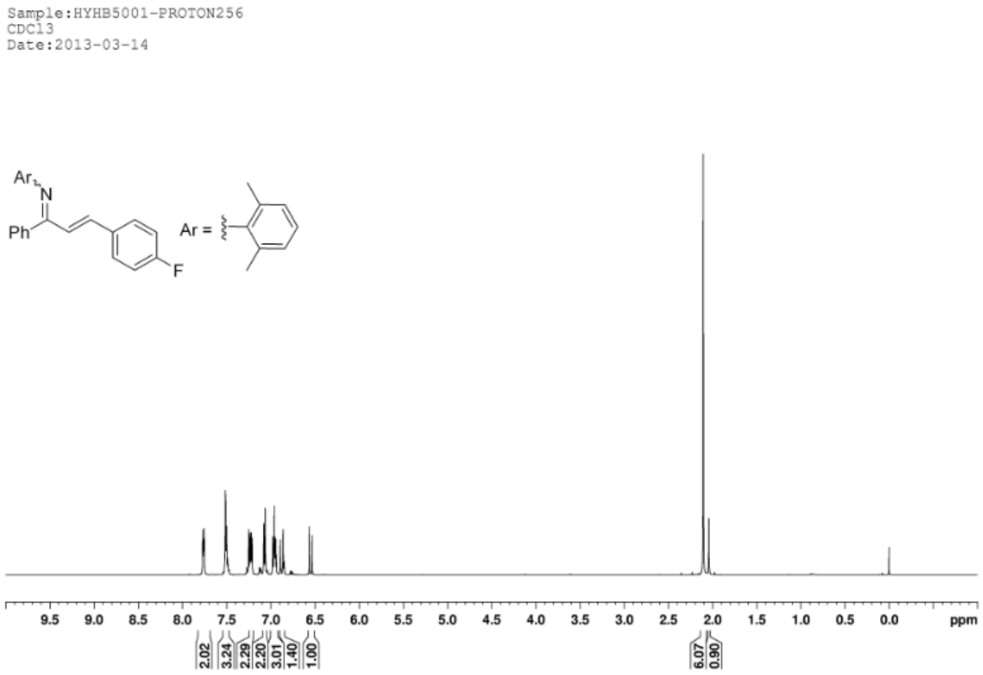

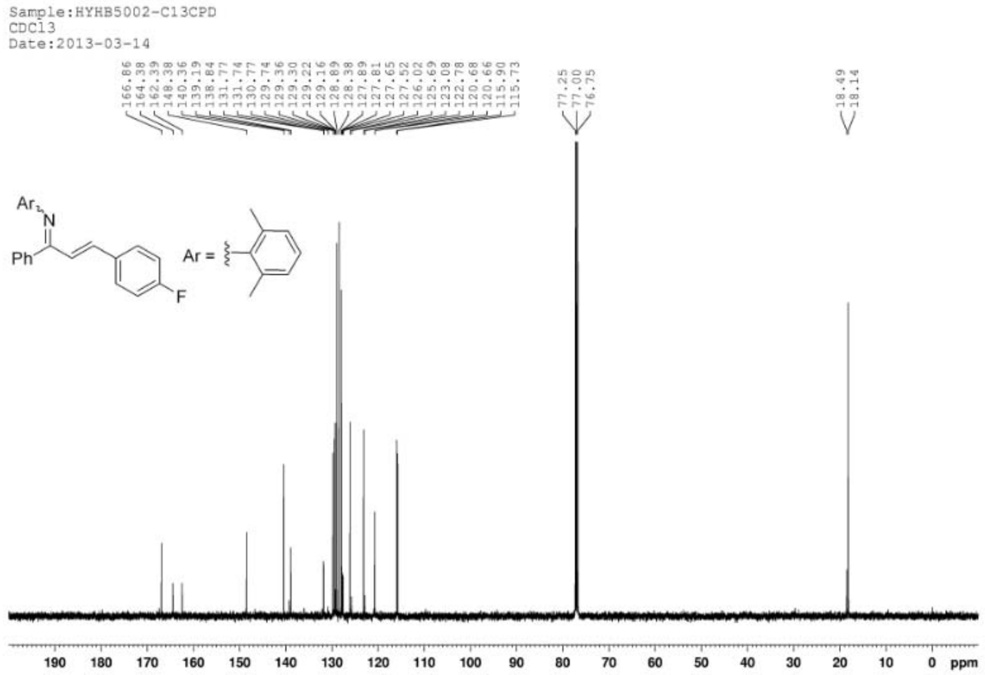

Supplementary Figure 6. ^{1}H NMR (500 MHz) and ^{13}C NMR (125 MHz) spectra of **2f** ($CDCl_3$)

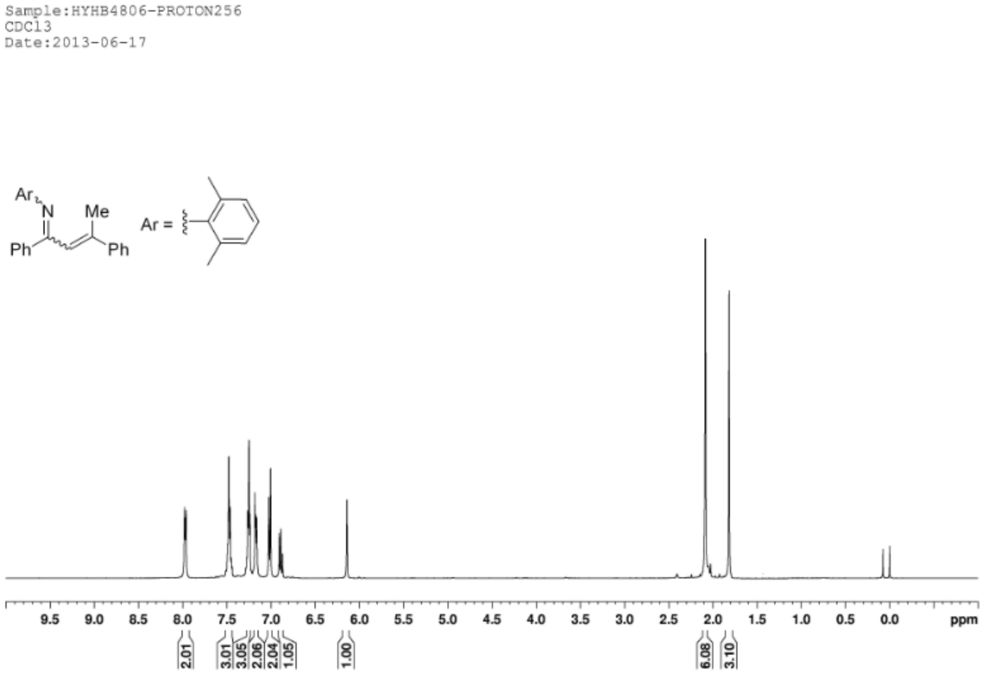

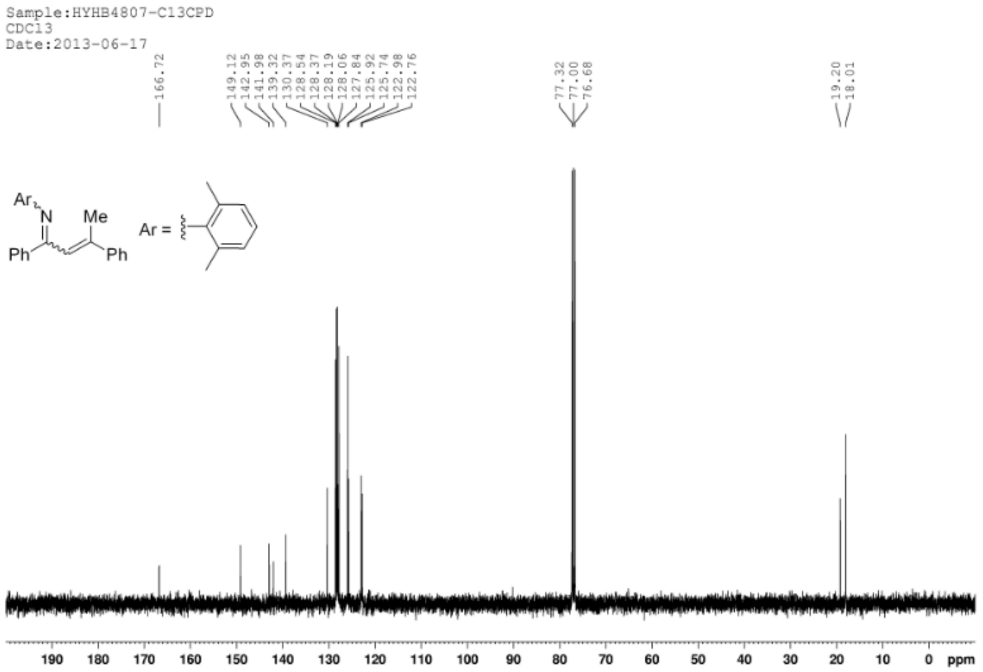

Supplementary Figure 7. ^{1}H NMR (400 MHz) and ^{13}C NMR (100 MHz) spectra of **2g** ($CDCl_3$)

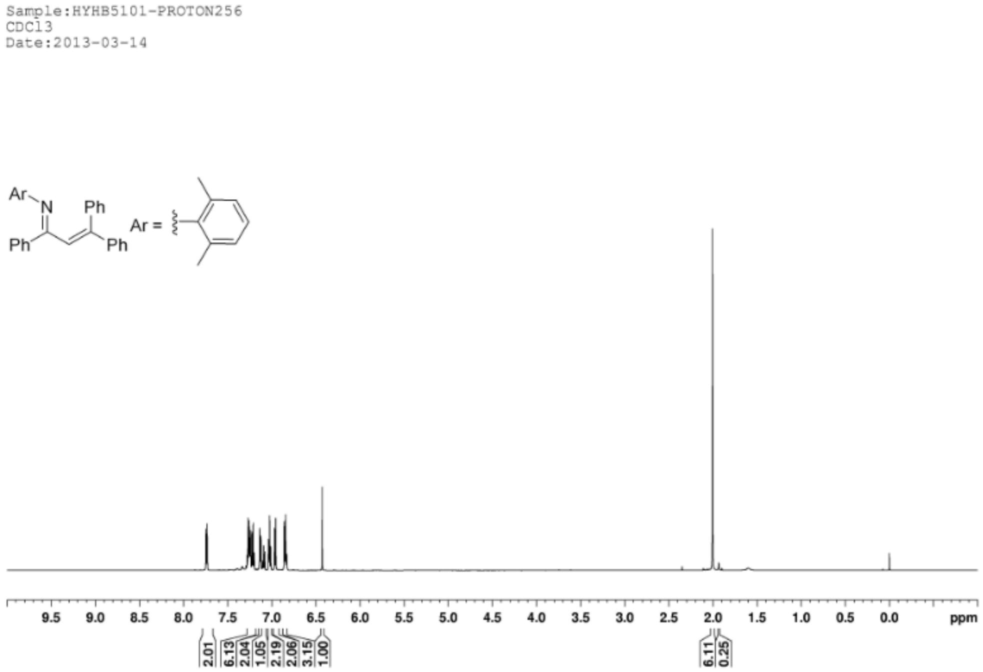

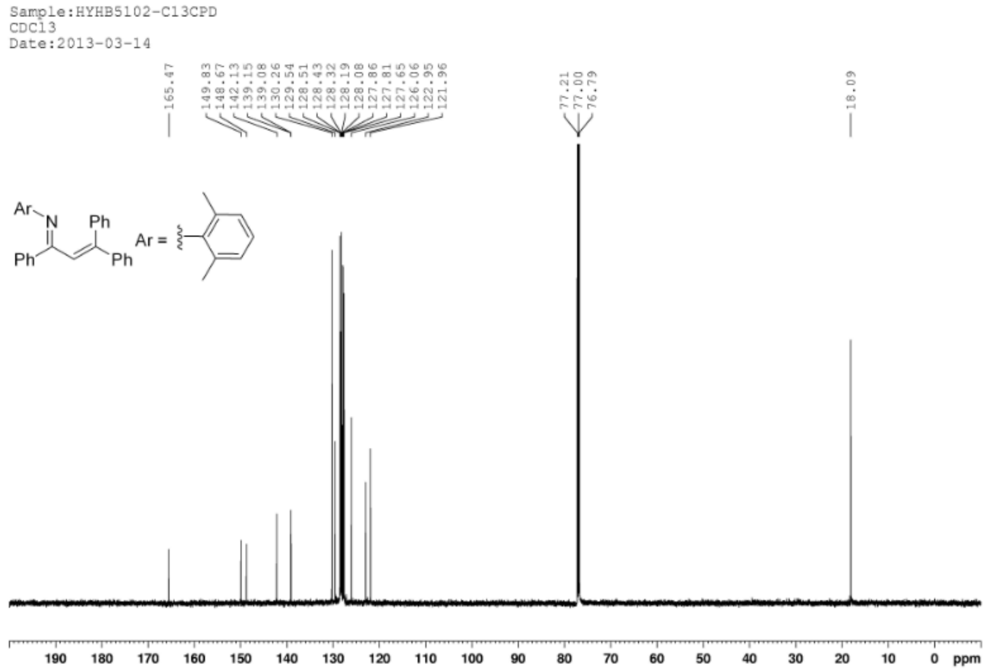

Supplementary Figure 8. ^{1}H NMR (500 MHz) and ^{13}C NMR (125 MHz) spectra of **2h** ($CDCl_3$)

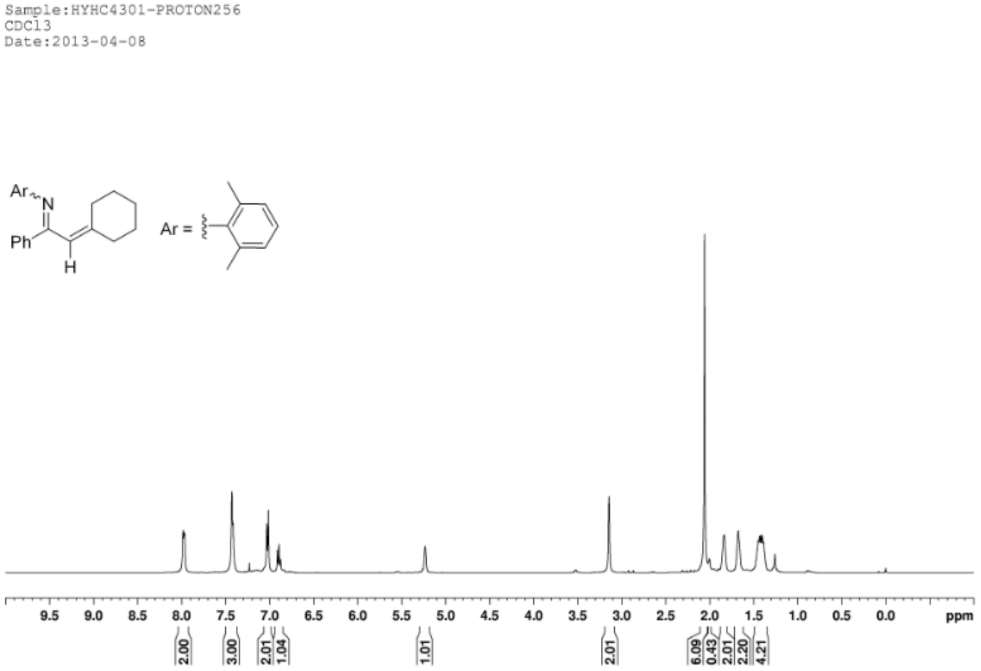

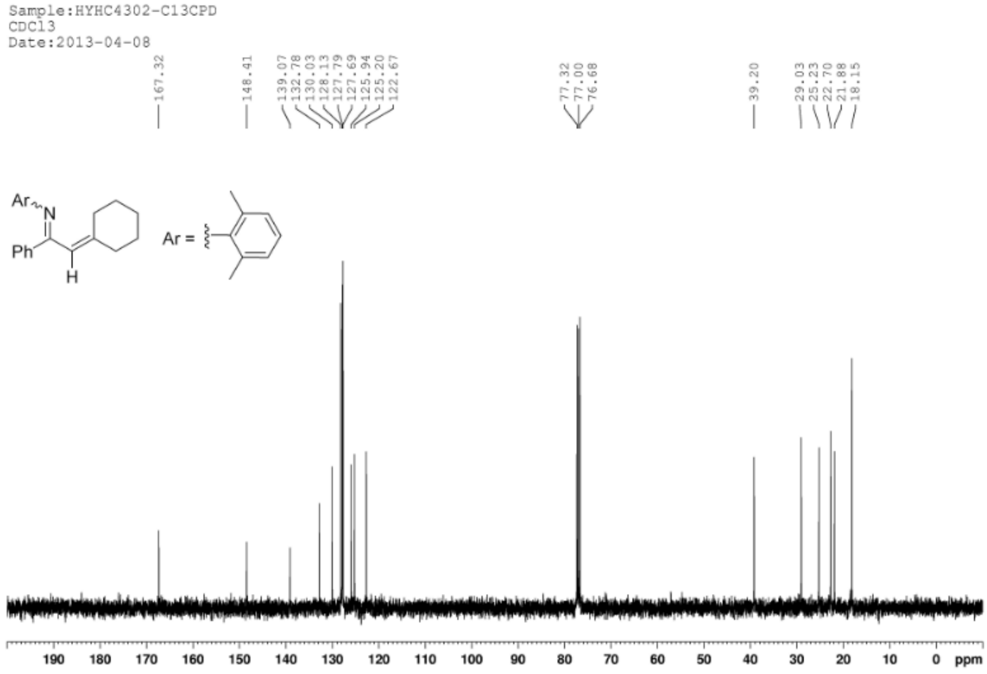

Supplementary Figure 9. ^{1}H NMR (400 MHz) and ^{13}C NMR (100 MHz) spectra of **2i** ($CDCl_3$)

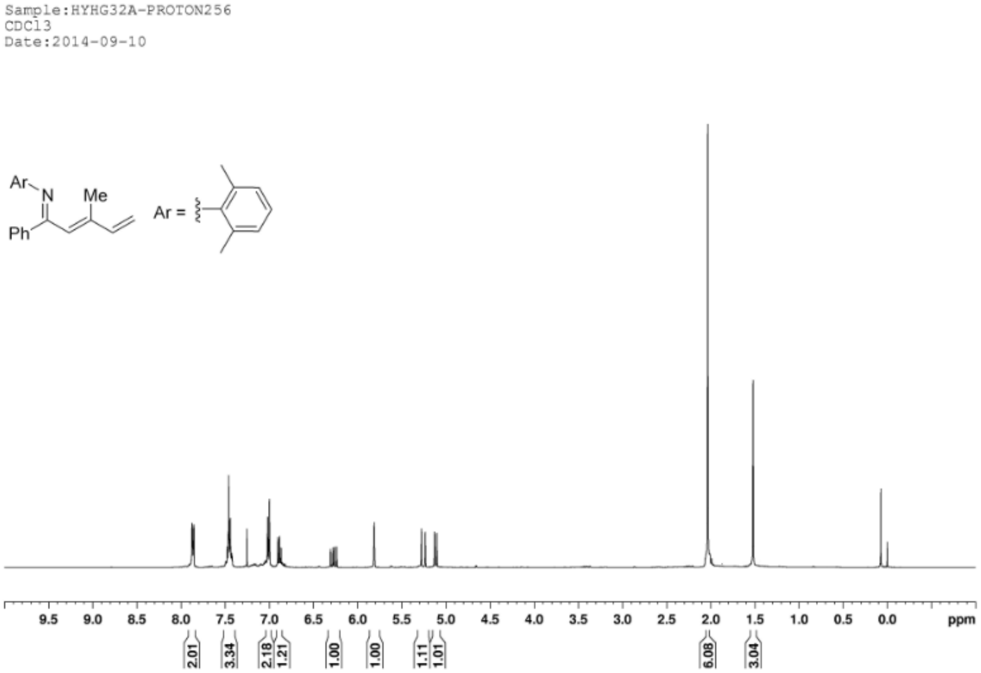

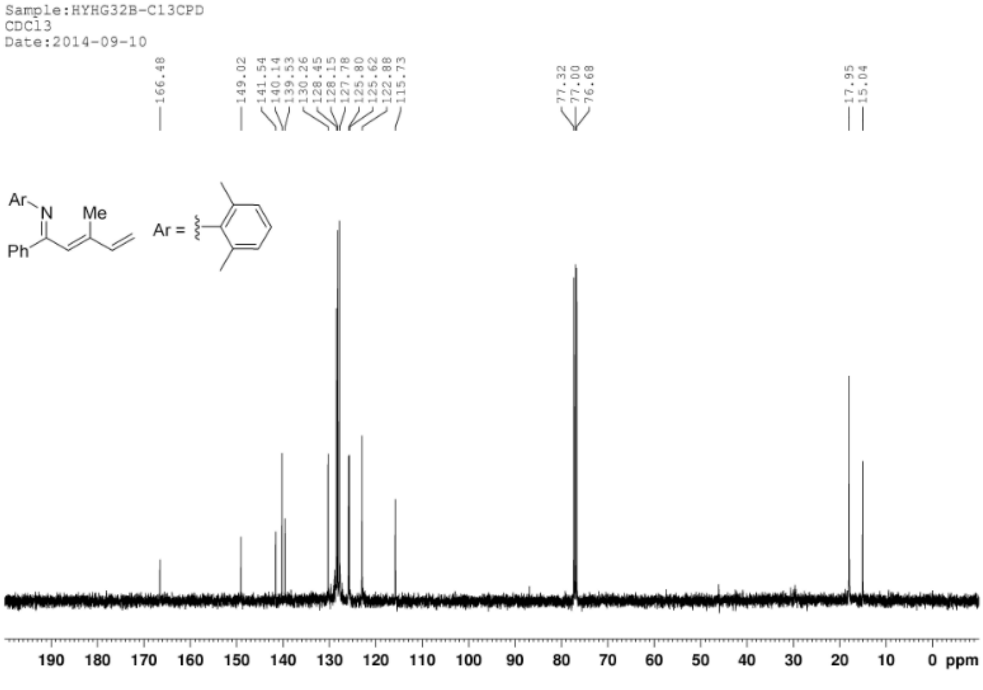

Supplementary Figure 10. ^{1}H NMR (400 MHz) and ^{13}C NMR (100 MHz) spectra of **2j** ($CDCl_3$)

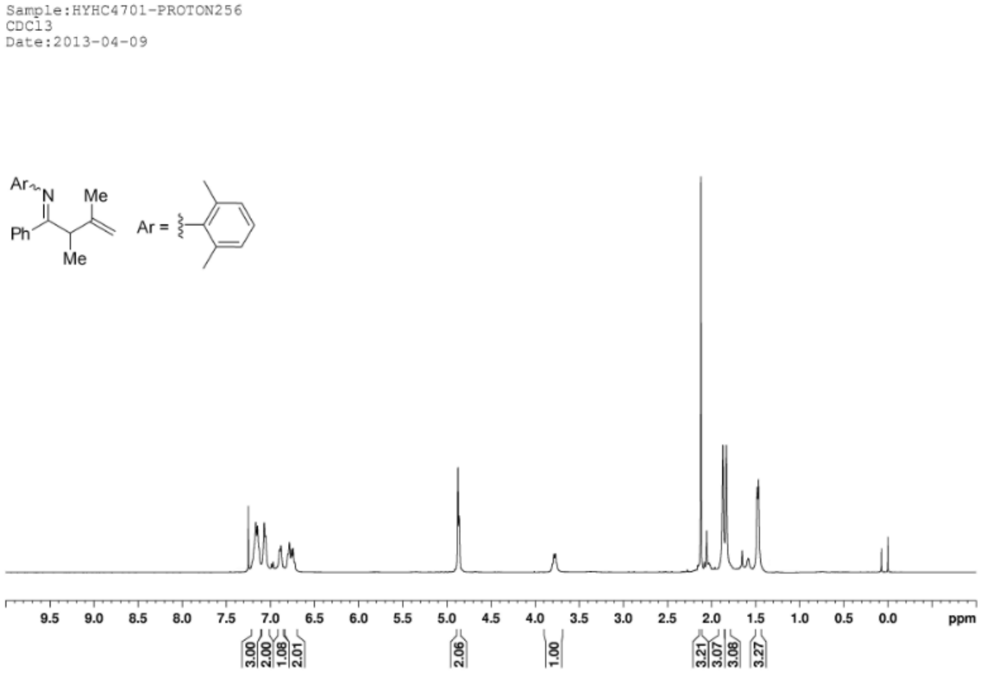

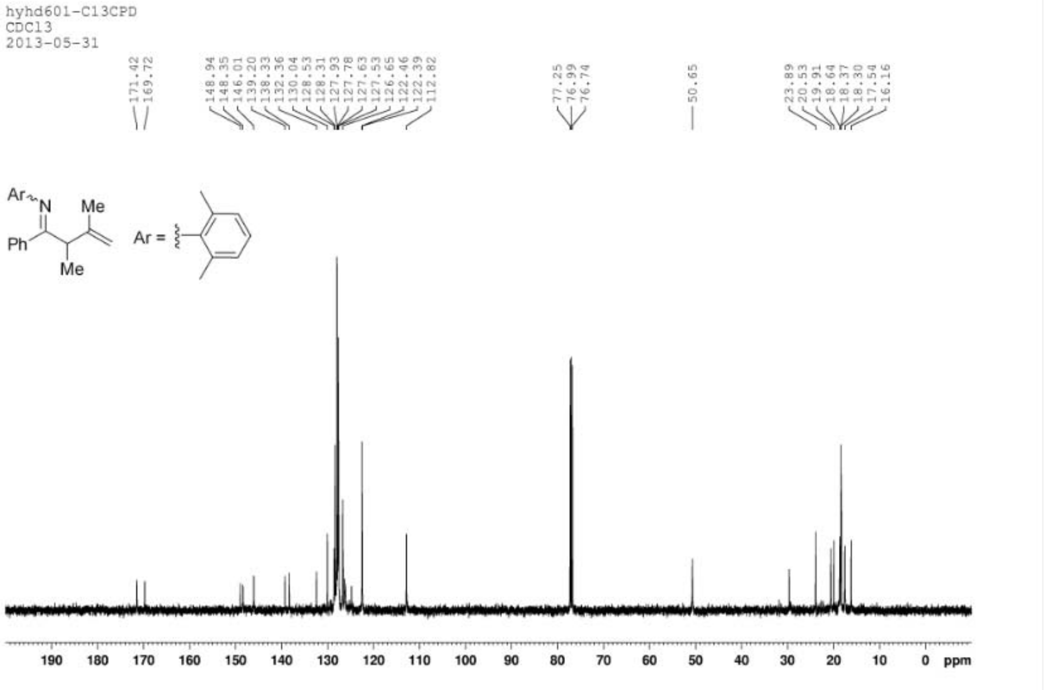

Supplementary Figure 11. ^{1}H NMR (400 MHz) and ^{13}C NMR (100 MHz) spectra of **2k** ($CDCl_3$)

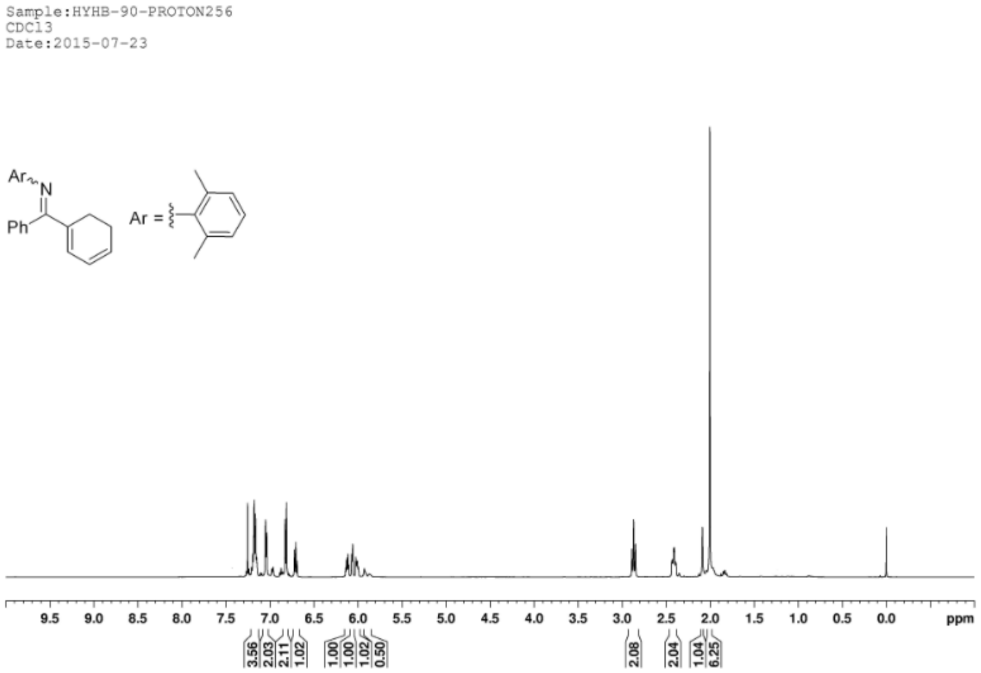

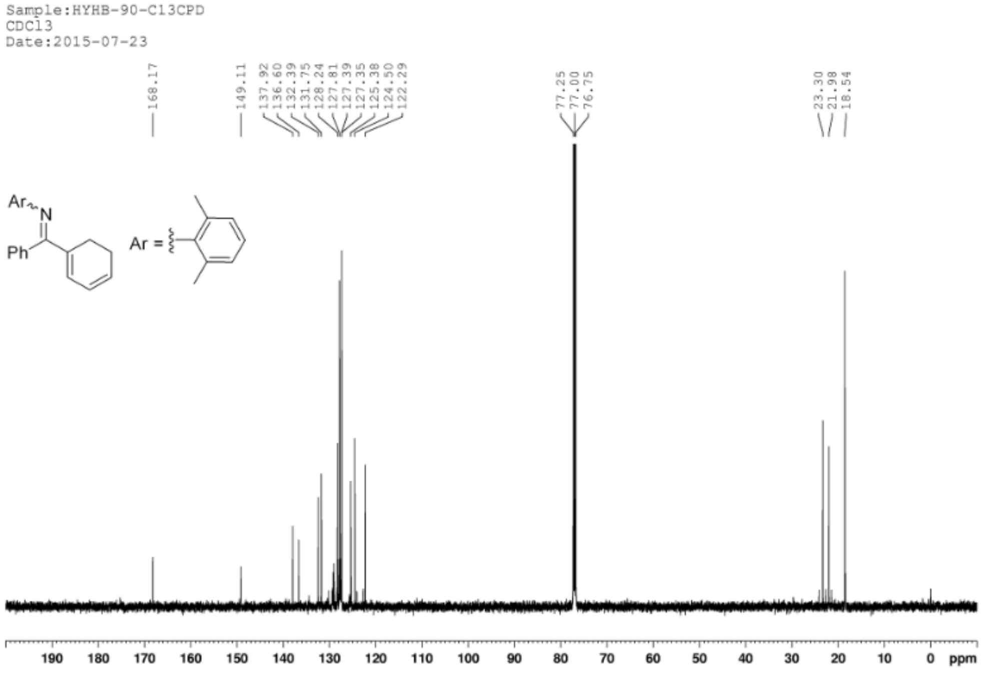

Supplementary Figure 12. ^{1}H NMR (400 MHz) and ^{13}C NMR (100 MHz) spectra of **2l** ($CDCl_3$)

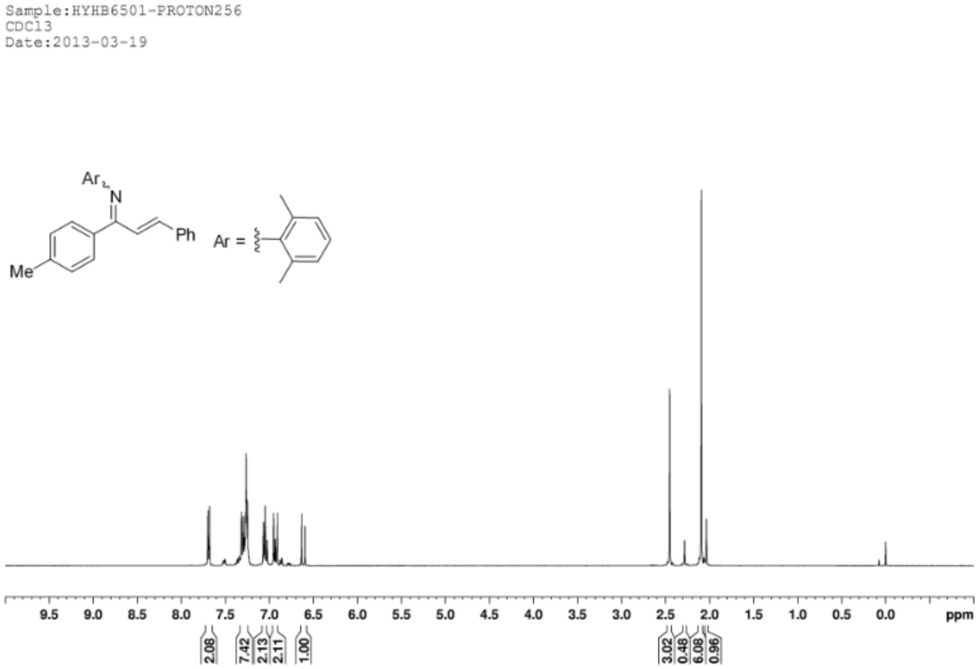

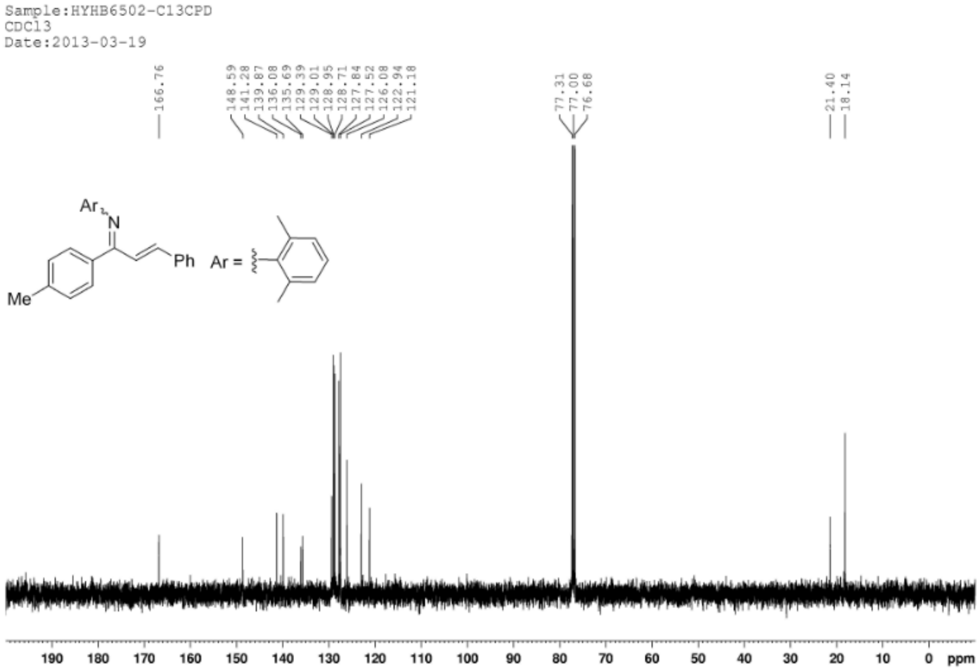

Supplementary Figure 13. ^{1}H NMR (400 MHz) and ^{13}C NMR (100 MHz) spectra of **2m** ($CDCl_3$)

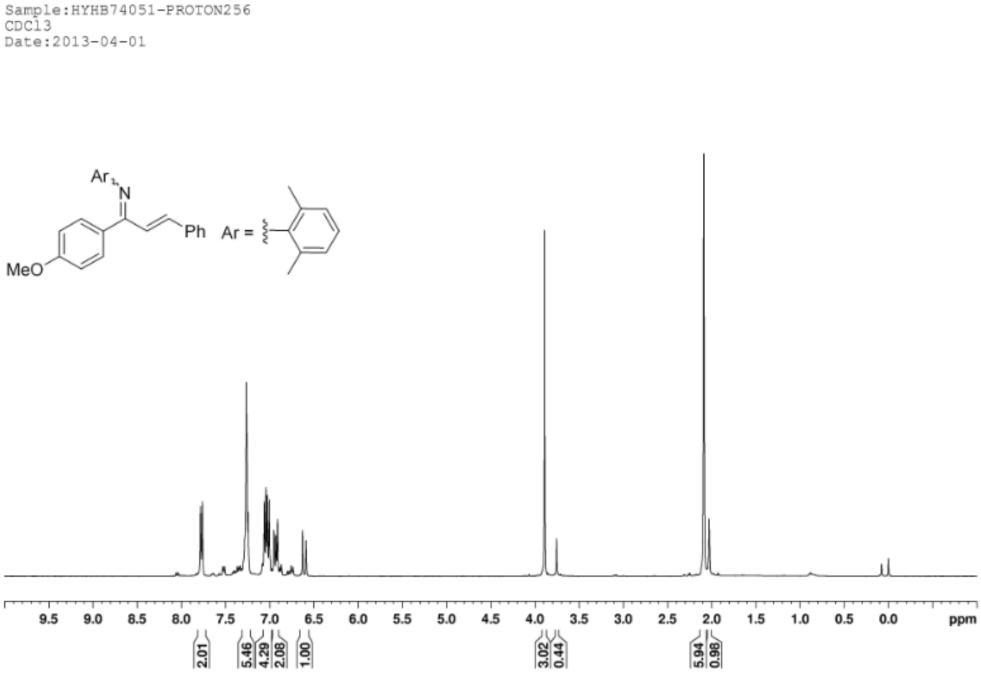

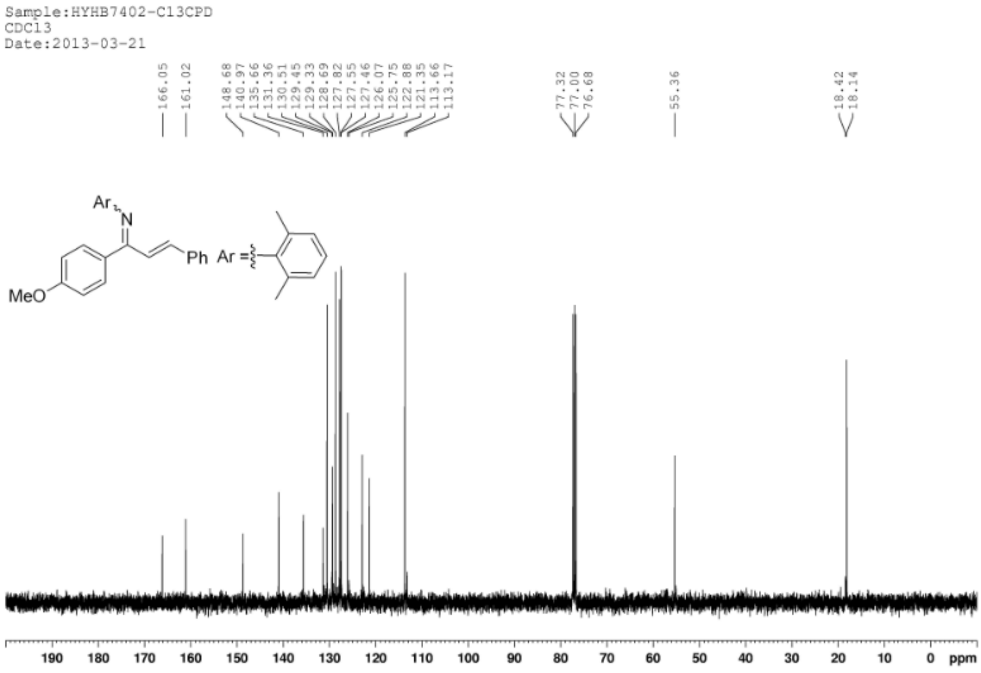

Supplementary Figure 14. ^{1}H NMR (400 MHz) and ^{13}C NMR (100 MHz) spectra of **2n** ($CDCl_3$)

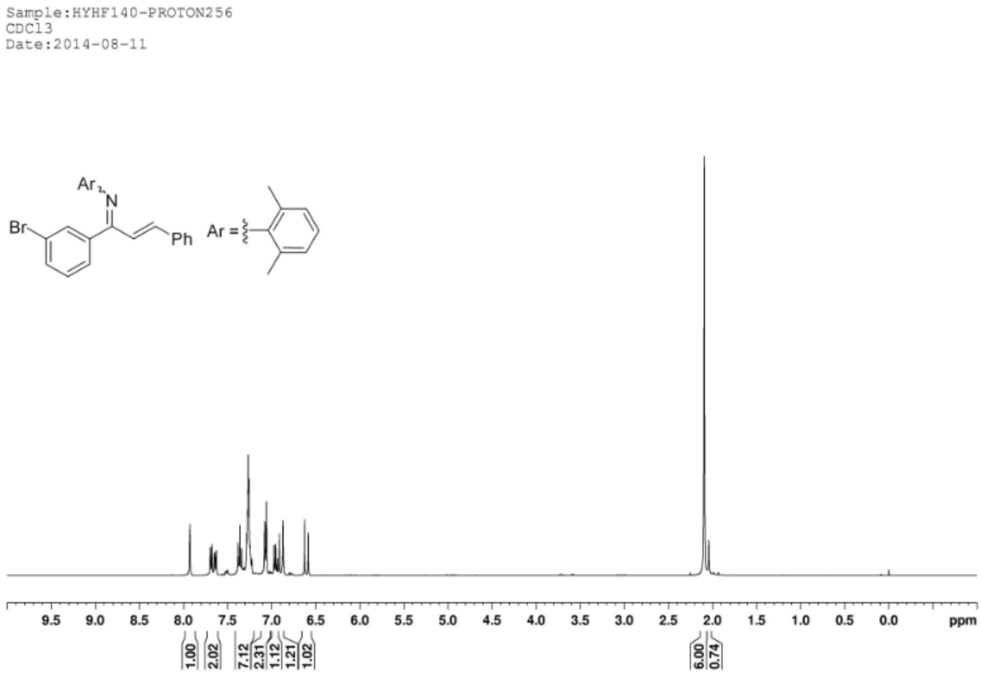

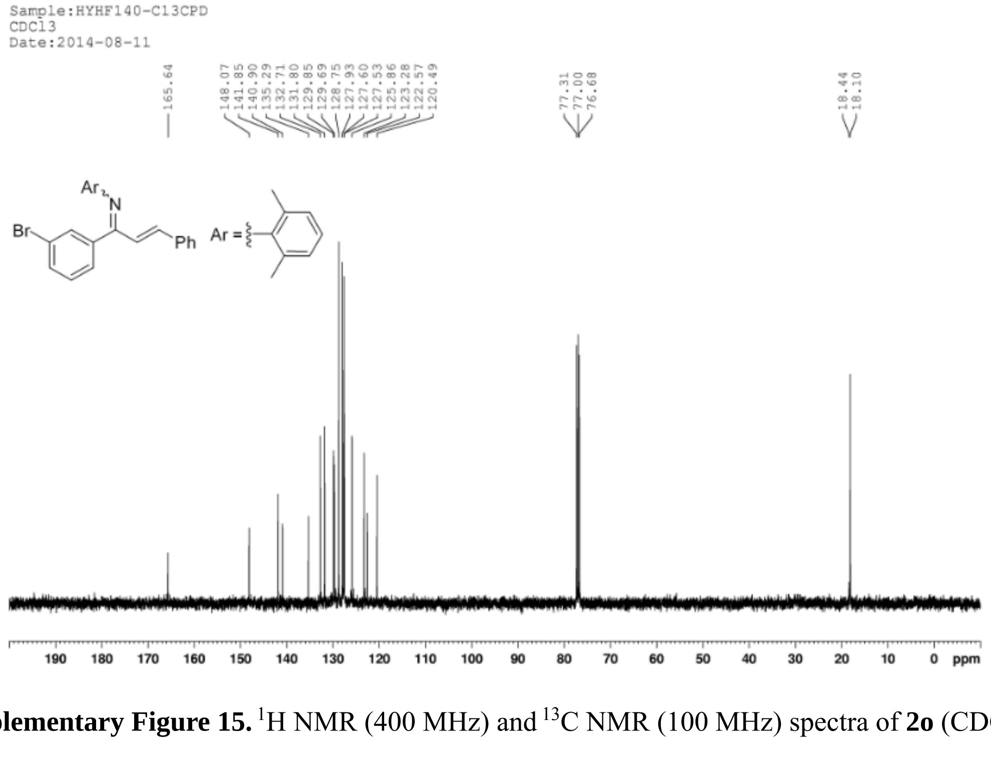

Supplementary Figure 15. ^{1}H NMR (400 MHz) and ^{13}C NMR (100 MHz) spectra of **2o** ($CDCl_3$)

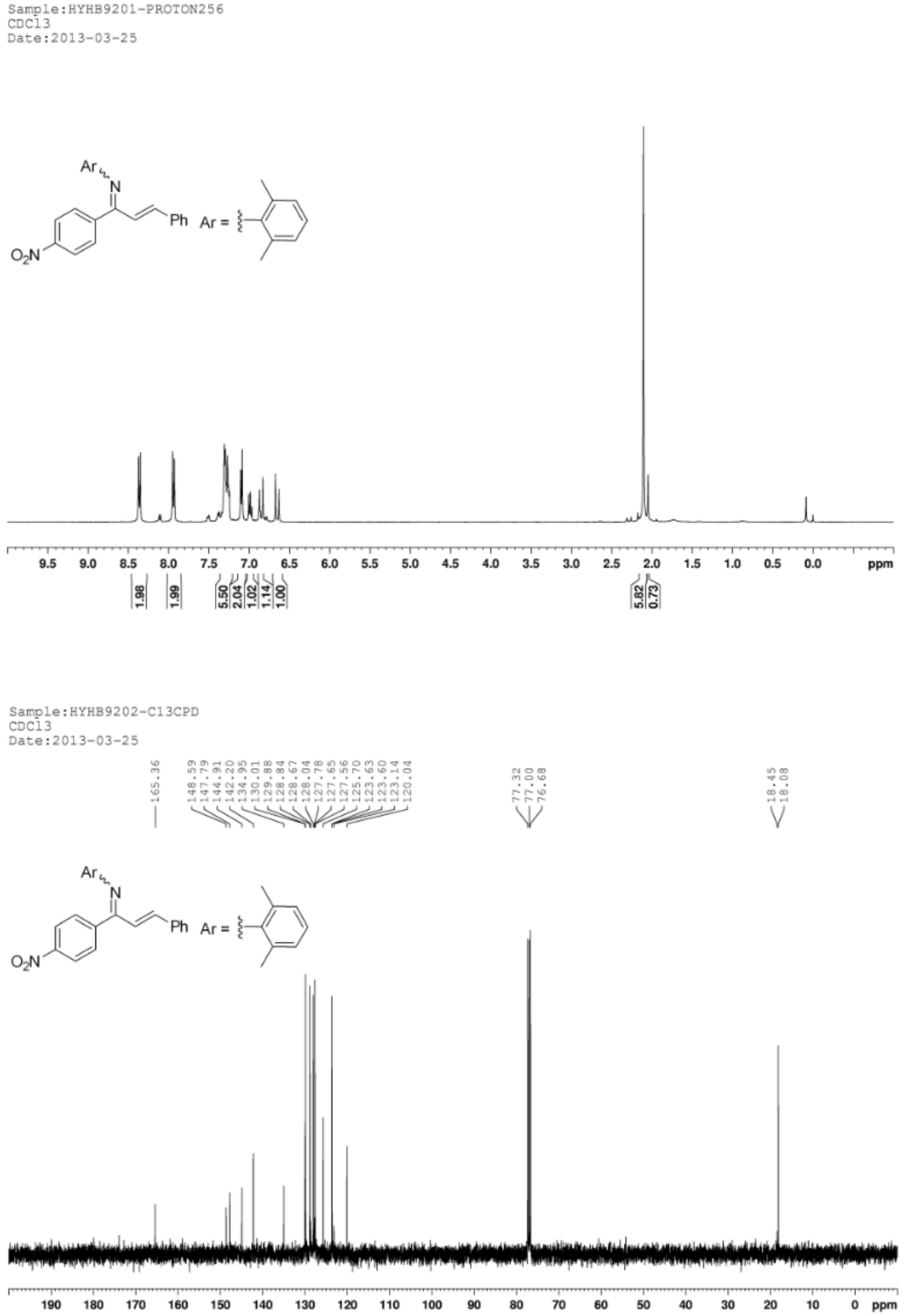

Supplementary Figure 16. ^{1}H NMR (400 MHz) and ^{13}C NMR (100 MHz) spectra of **2p** ($CDCl_3$)

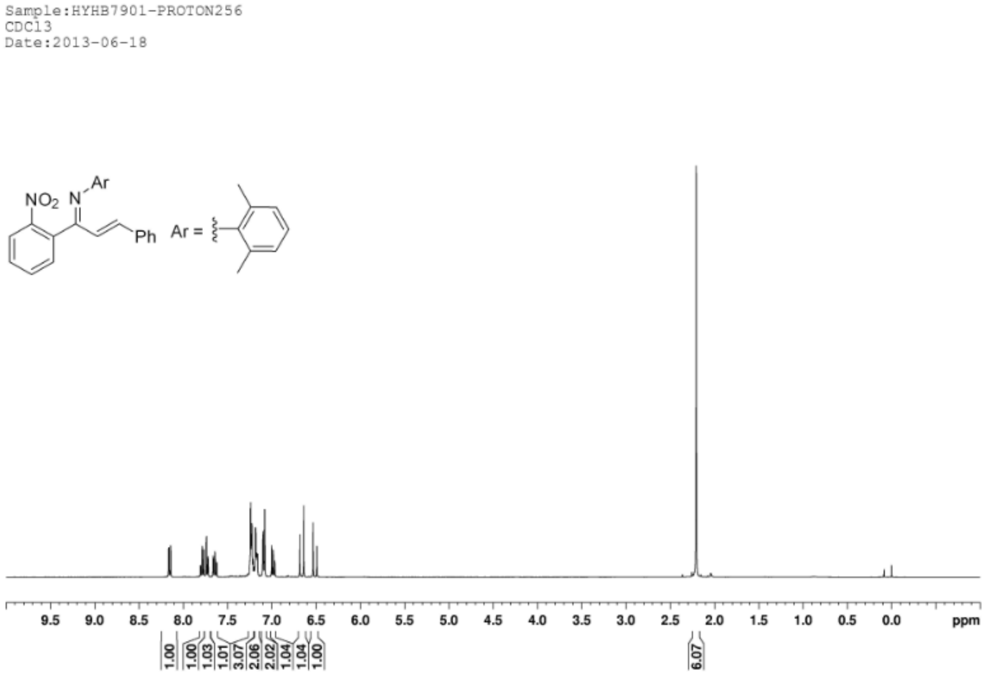

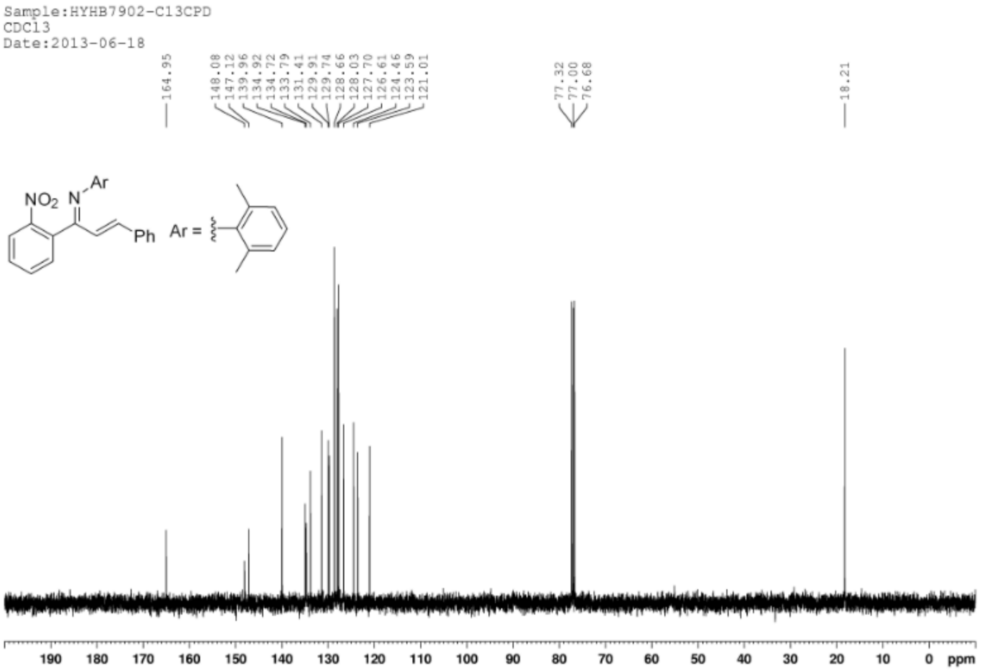

Supplementary Figure 17. ^{1}H NMR (400 MHz) and ^{13}C NMR (100 MHz) spectra of **2q** ($CDCl_3$)

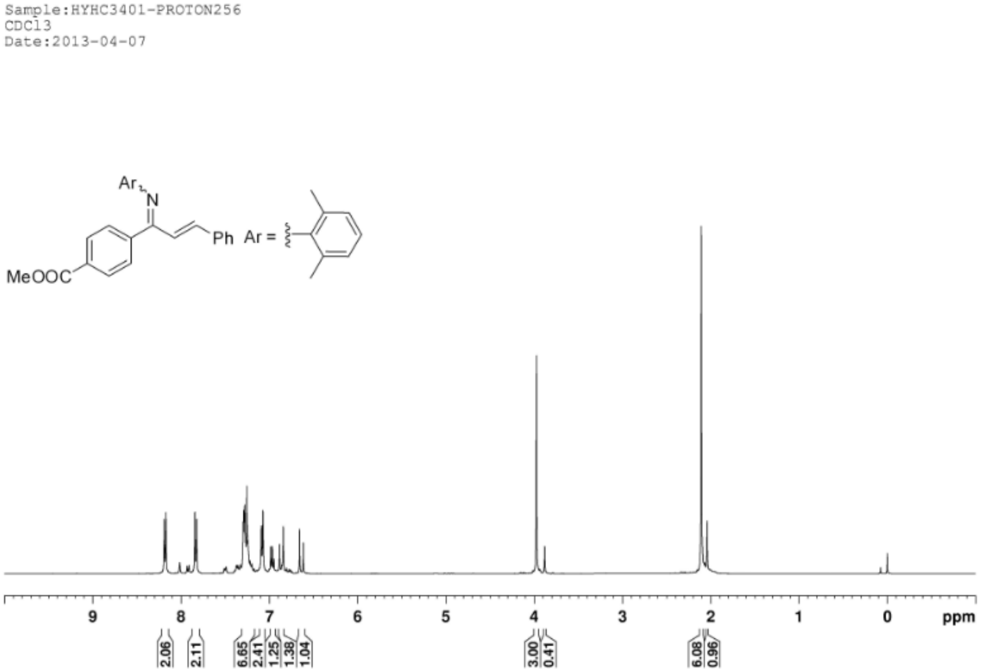

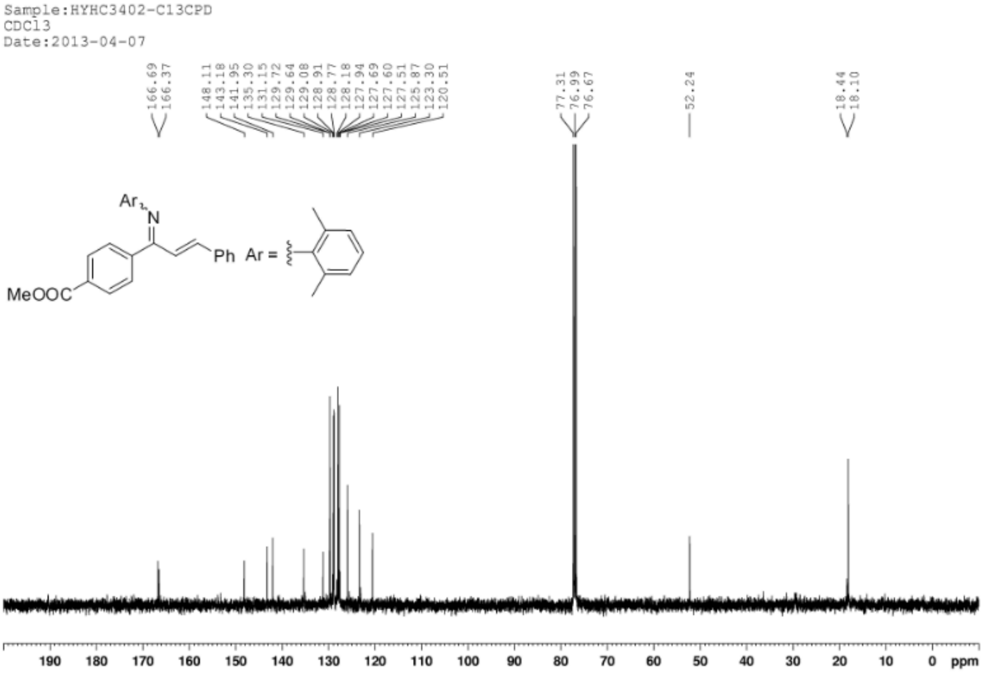

Supplementary Figure 18. ^{1}H NMR (400 MHz) and ^{13}C NMR (100 MHz) spectra of **2r** ($CDCl_3$)

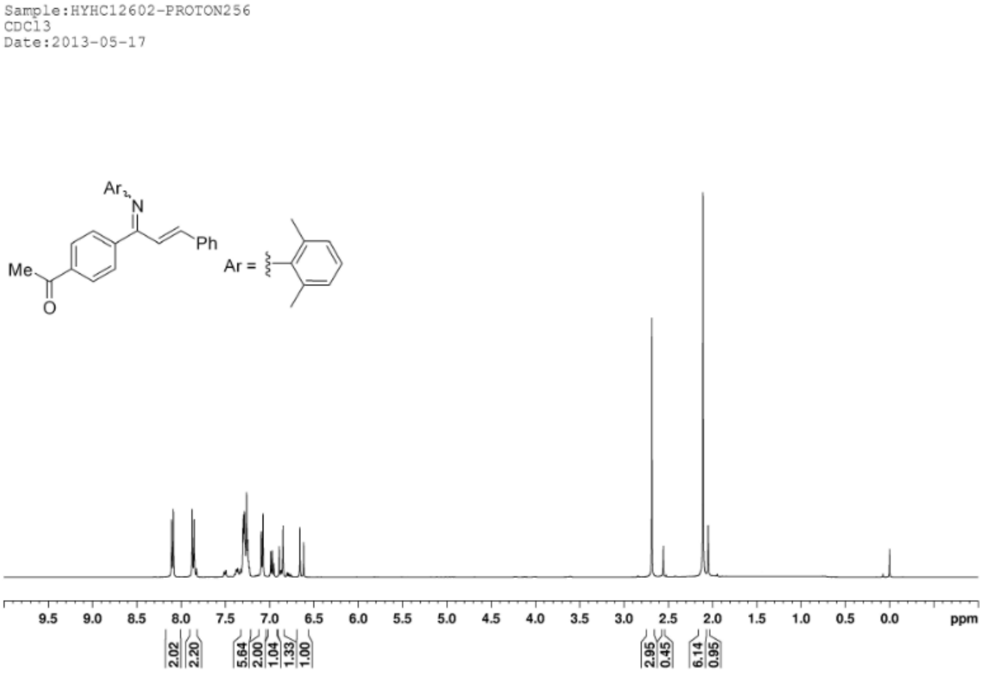

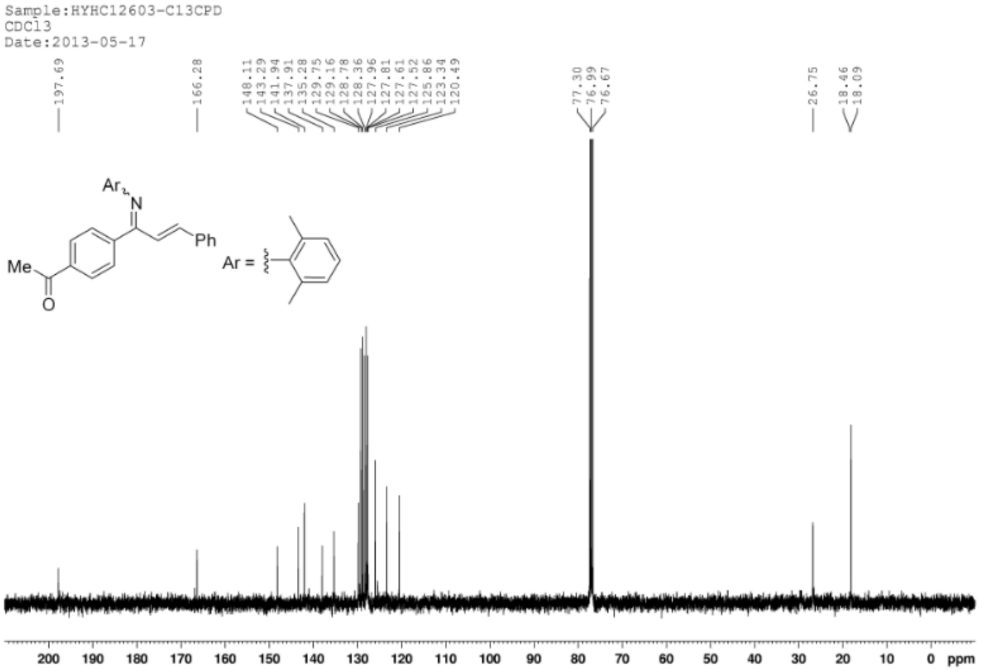

Supplementary Figure 19. ^{1}H NMR (400 MHz) and ^{13}C NMR (100 MHz) spectra of **2s** ($CDCl_3$)

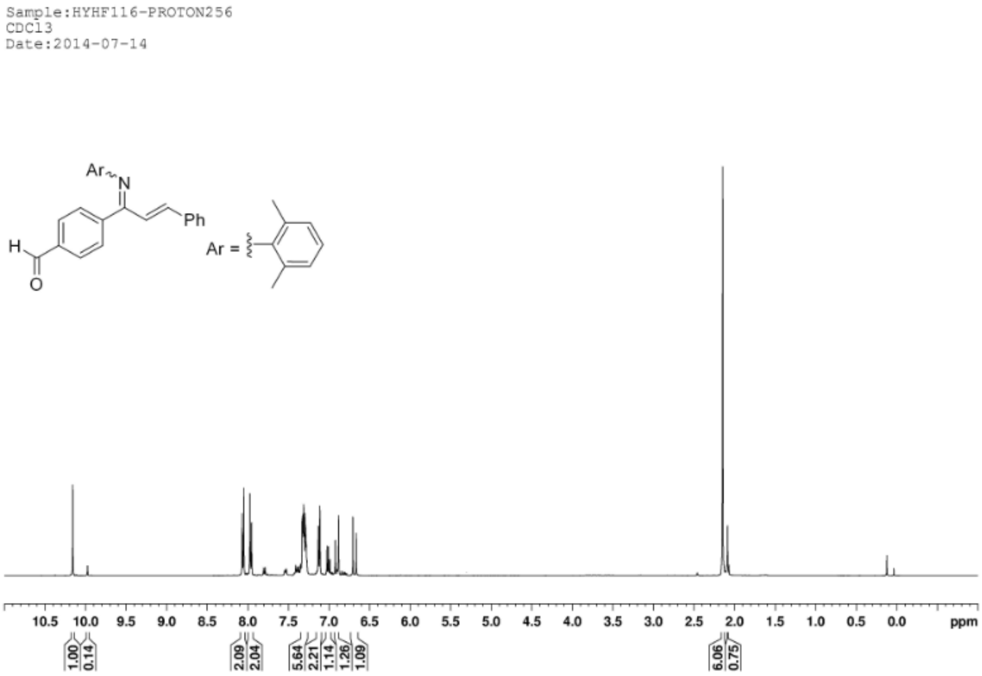

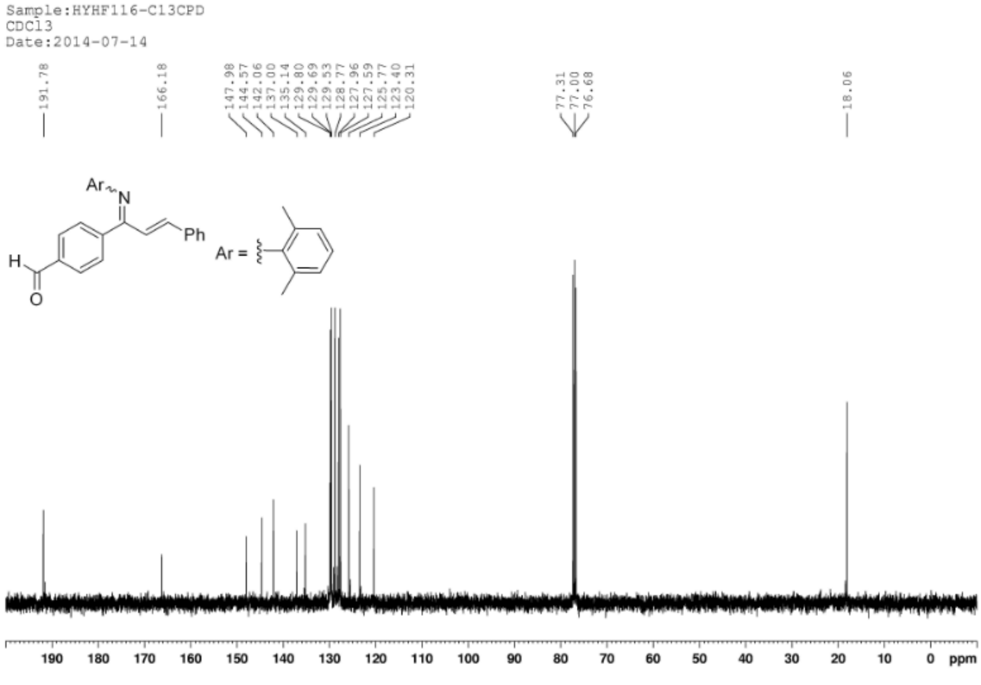

Supplementary Figure 20. ^{1}H NMR (400 MHz) and ^{13}C NMR (100 MHz) spectra of **2t** ($CDCl_3$)

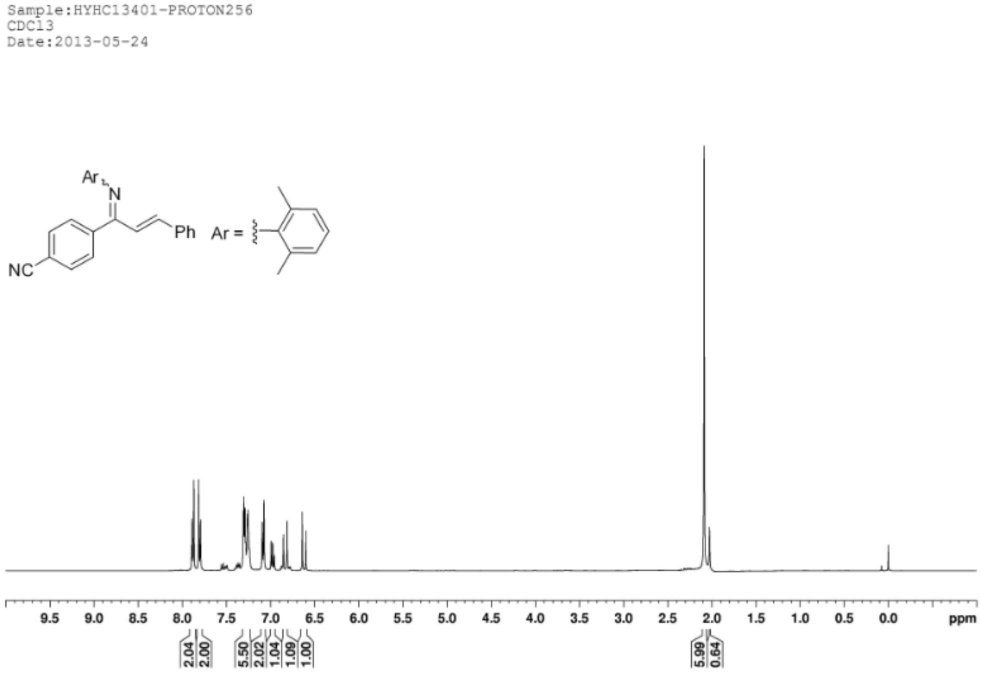

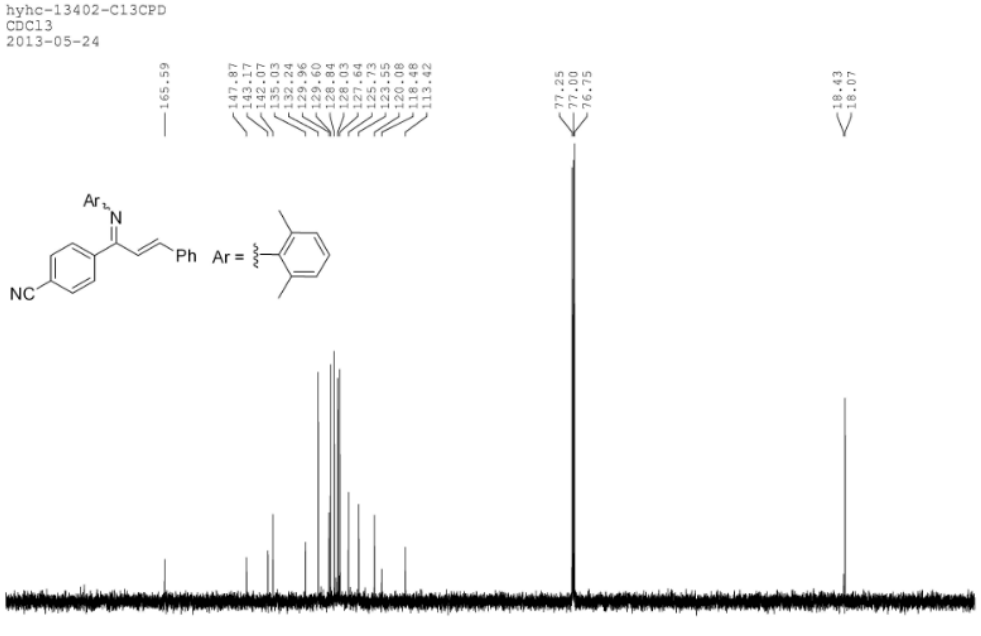

Supplementary Figure 21. ^{1}H NMR (400 MHz) and ^{13}C NMR (100 MHz) spectra of **2u** ($CDCl_3$)

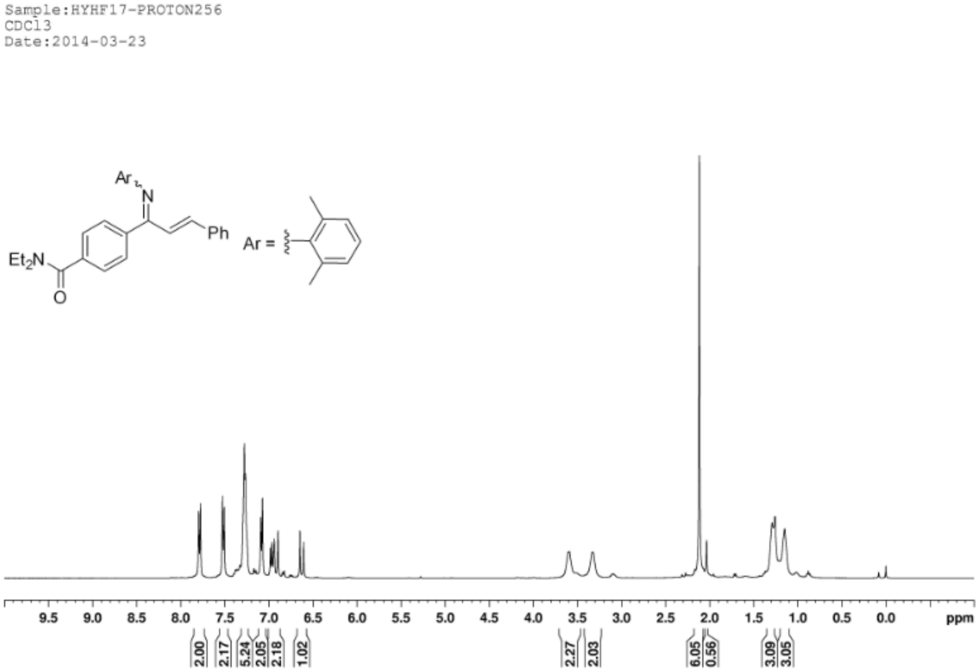

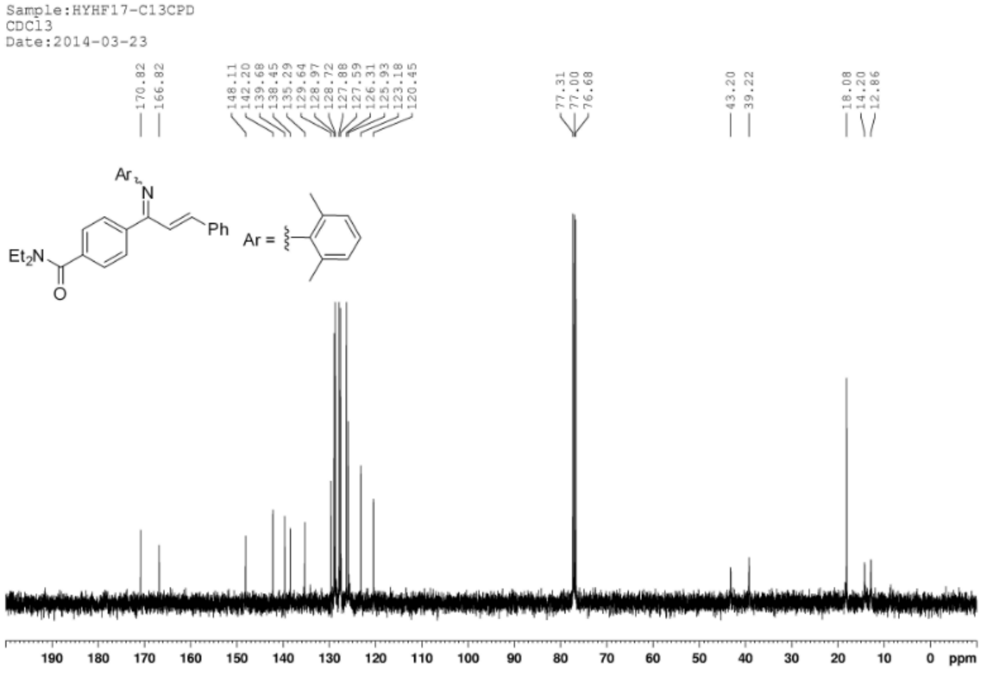

Supplementary Figure 22. ^{1}H NMR (400 MHz) and ^{13}C NMR (100 MHz) spectra of **2v** ($CDCl_3$)

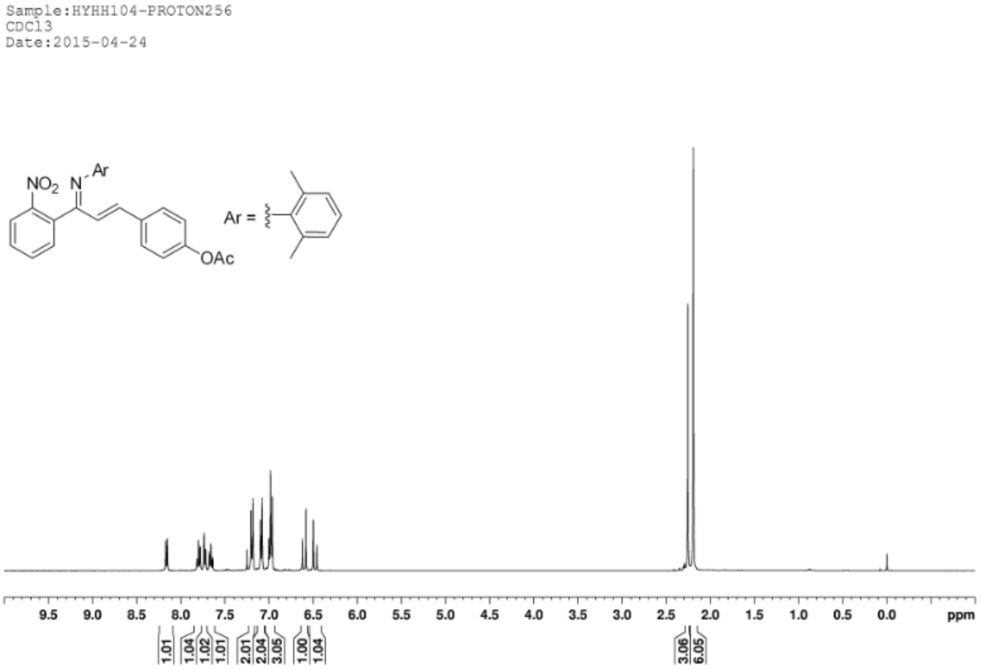

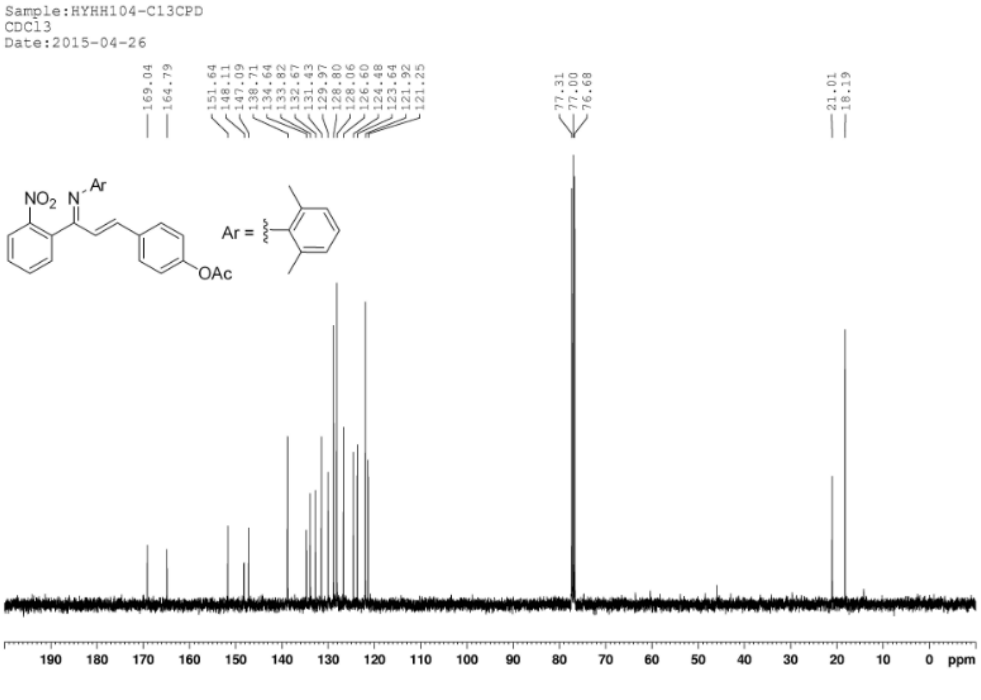

Supplementary Figure 23. ^{1}H NMR (500 MHz) and ^{13}C NMR (125 MHz) spectra of **2w** ($CDCl_3$)

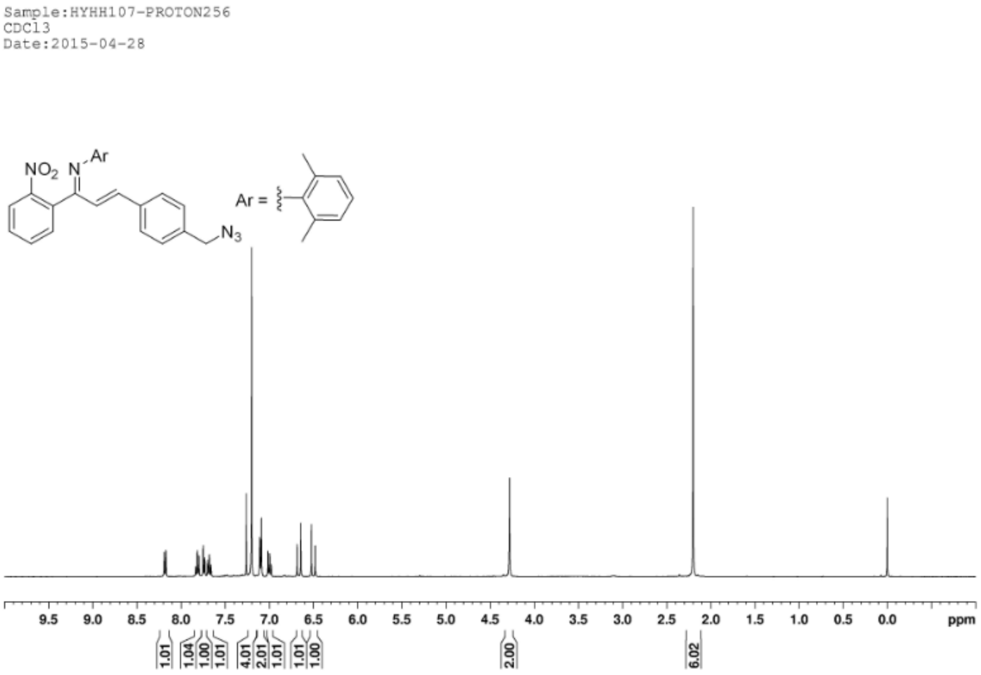

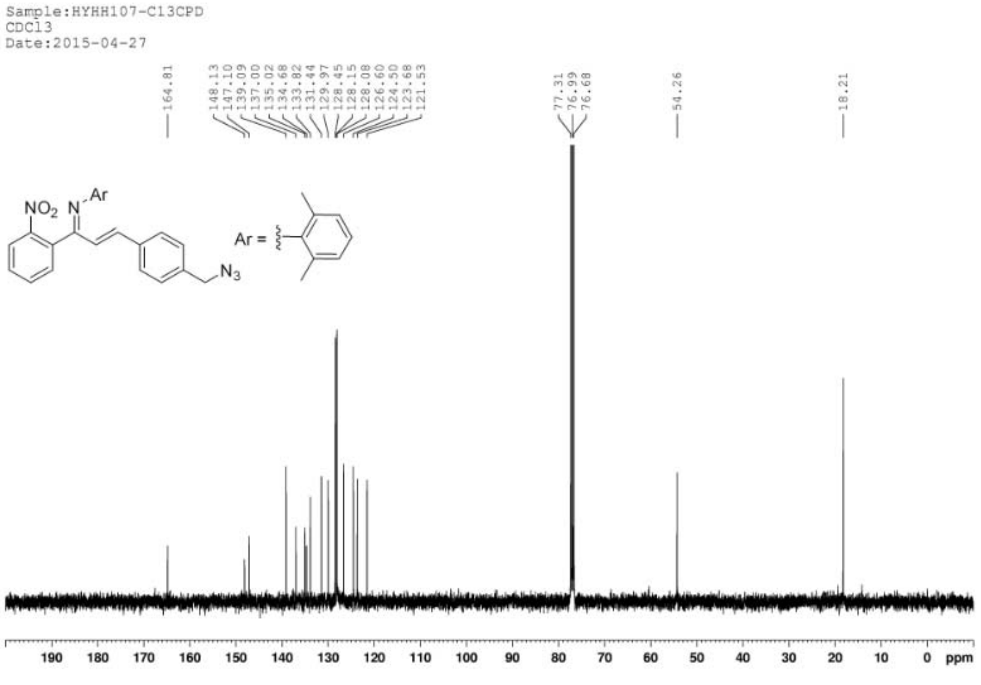

Supplementary Figure 24. ^{1}H NMR (500 MHz) and ^{13}C NMR (125 MHz) spectra of **2x** ($CDCl_3$)

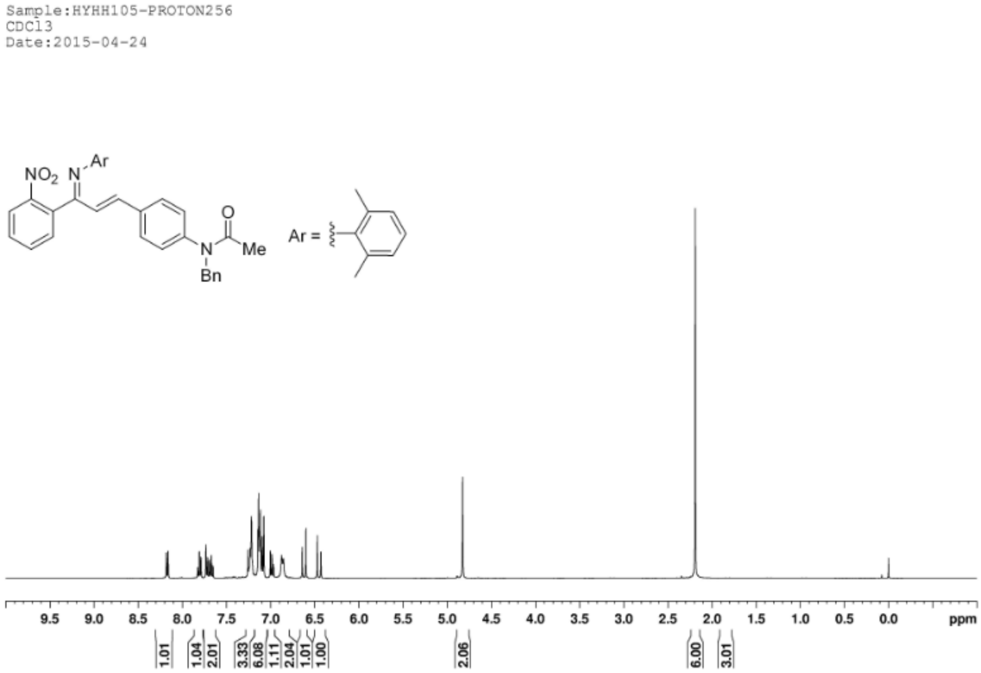

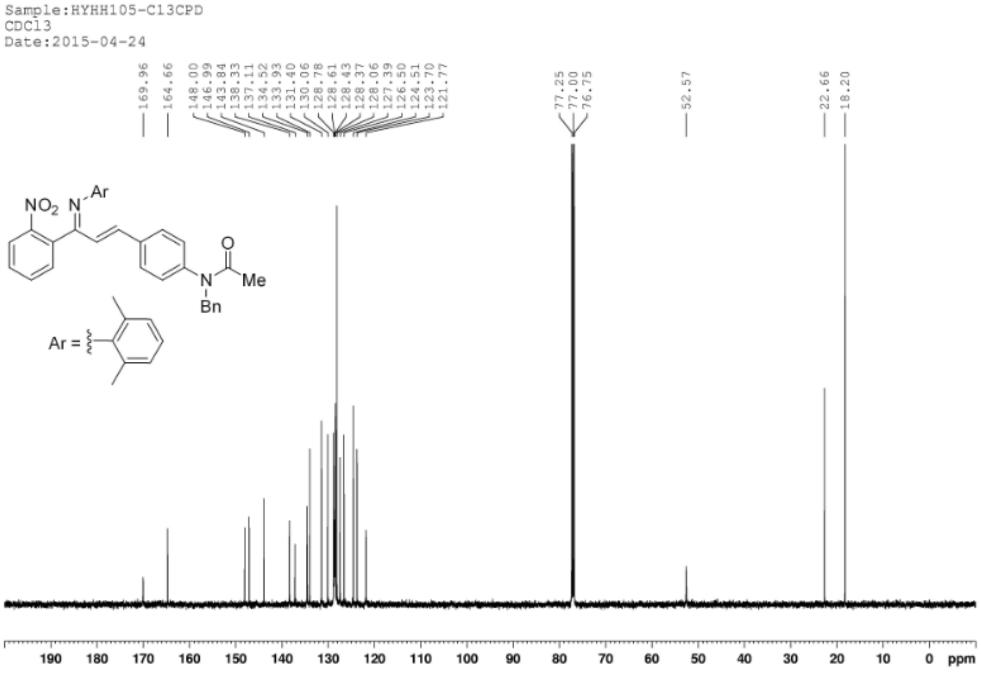

Supplementary Figure 25. ^{1}H NMR (500 MHz) and ^{13}C NMR (100 MHz) spectra of **2y** ($CDCl_3$)

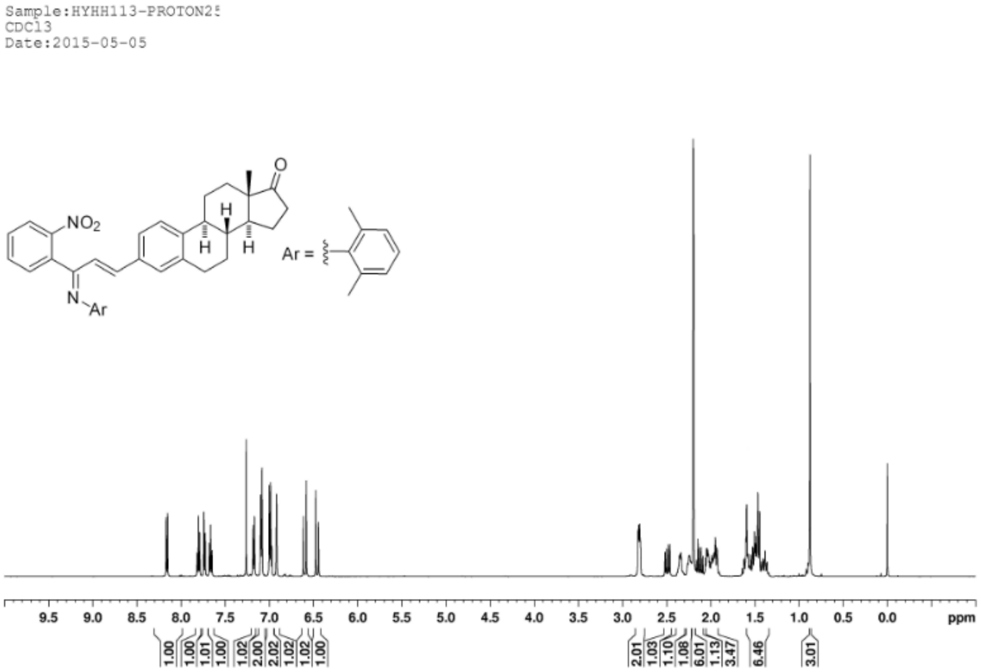

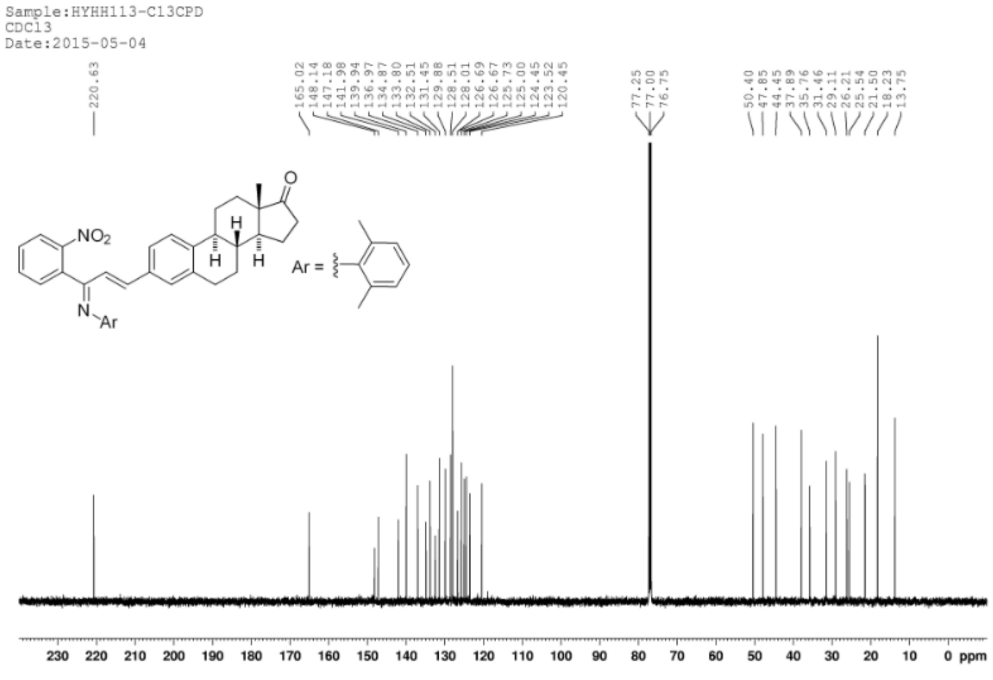

Supplementary Figure 26. ^{1}H NMR (500 MHz) and ^{13}C NMR (125 MHz) spectra of **2z** ($CDCl_3$)

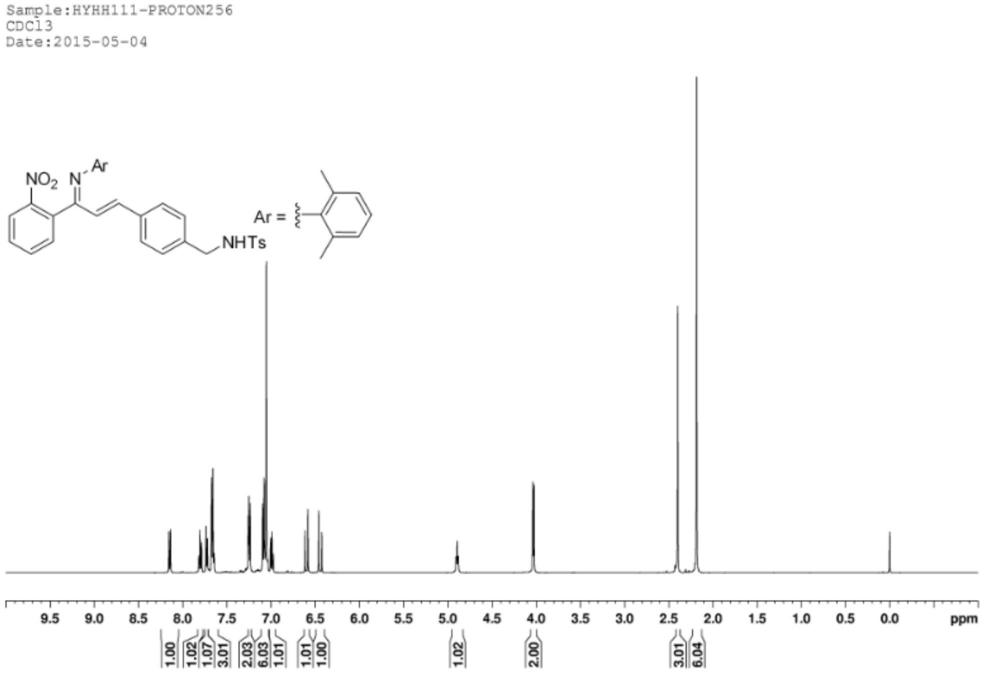

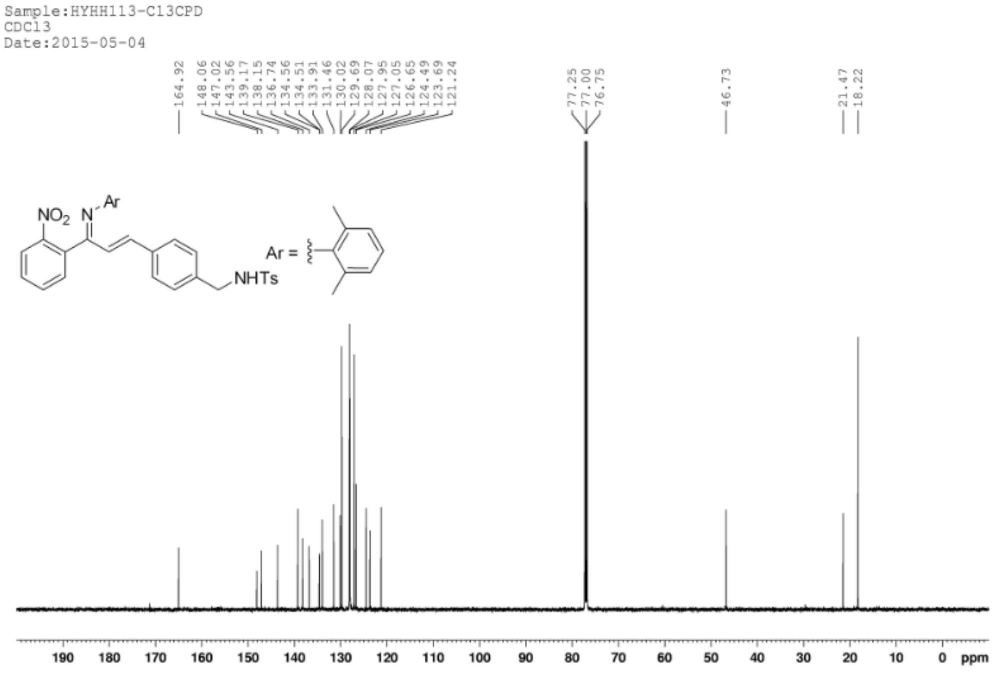

Supplementary Figure 27. ^{1}H NMR (500 MHz) and ^{13}C NMR (125 MHz) spectra of **2aa** ($CDCl_3$)

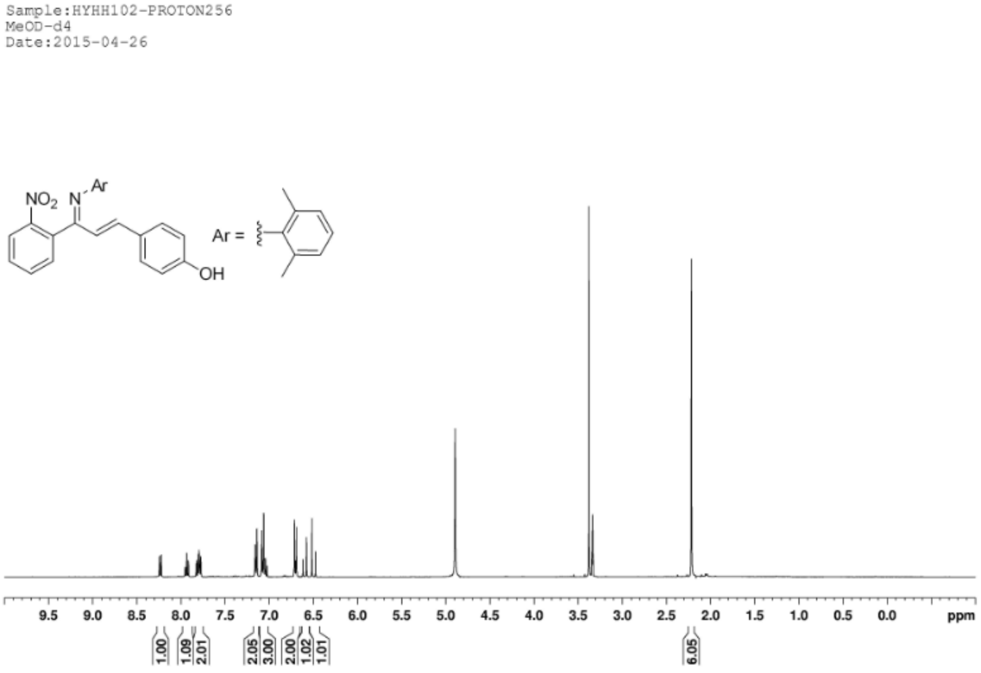

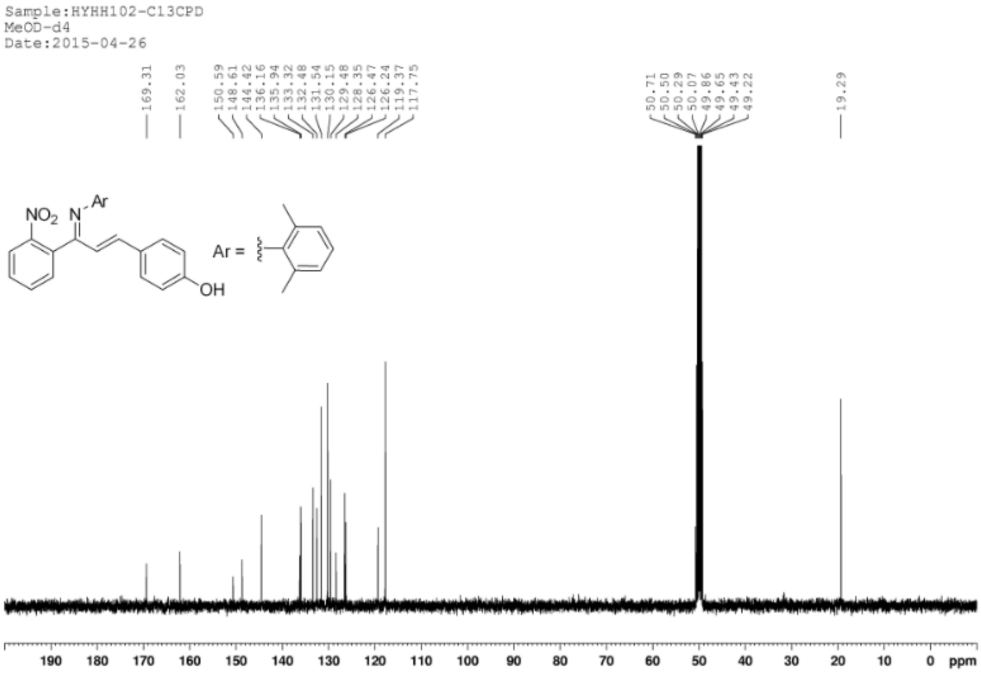

Supplementary Figure 28. ^{1}H NMR (500 MHz) and ^{13}C NMR (125 MHz) spectra of **2ab** (MD_3OD)

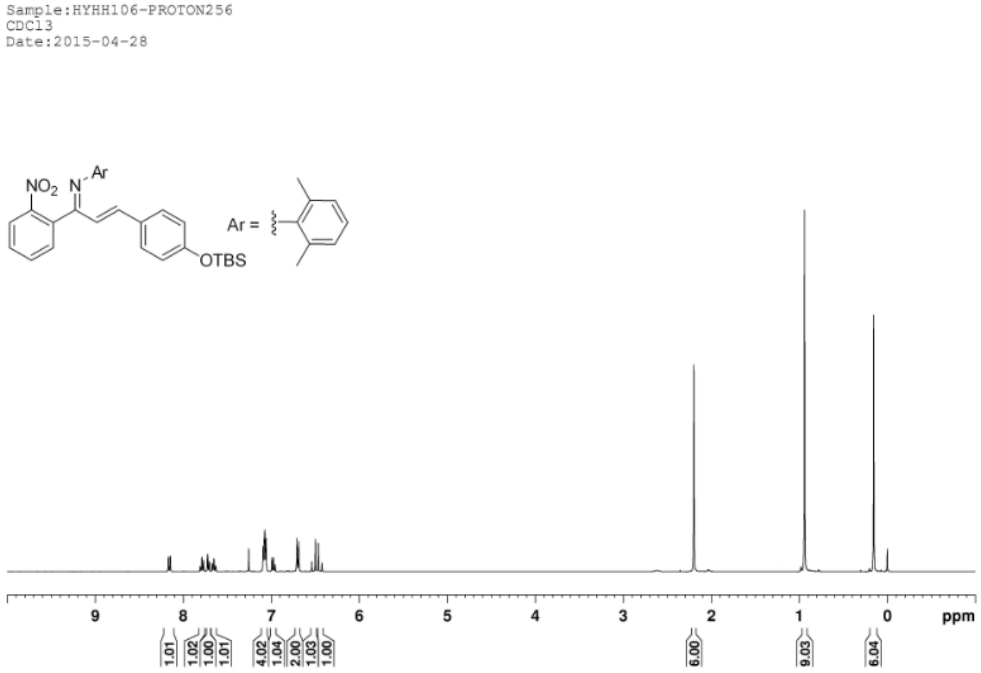

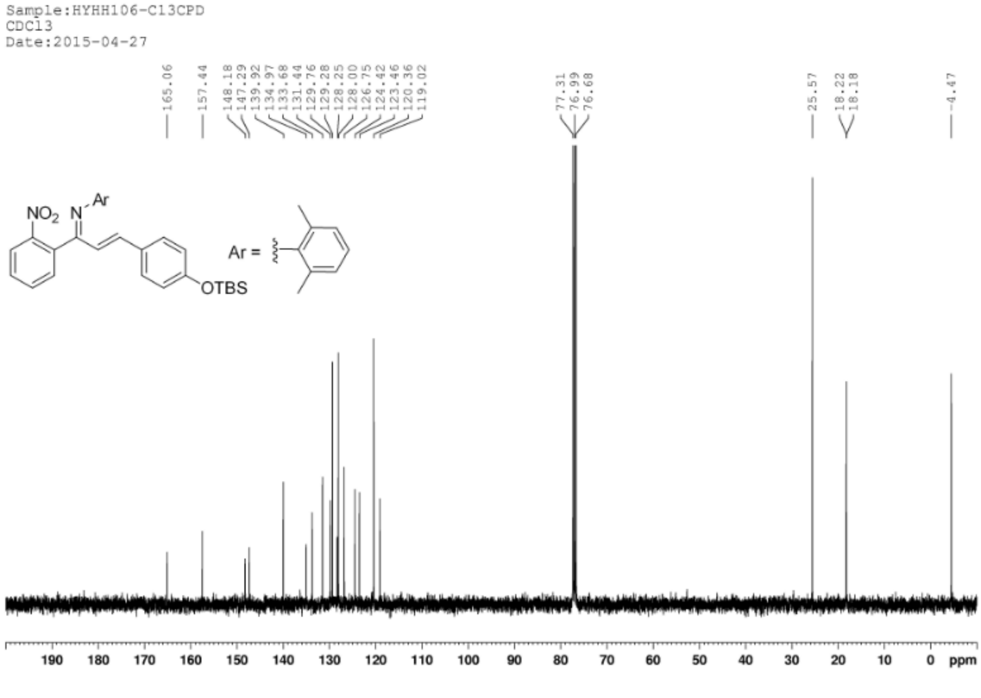

Supplementary Figure 29. ^{1}H NMR (500 MHz) and ^{13}C NMR (125 MHz) spectra of **2ac** ($CDCl_3$)

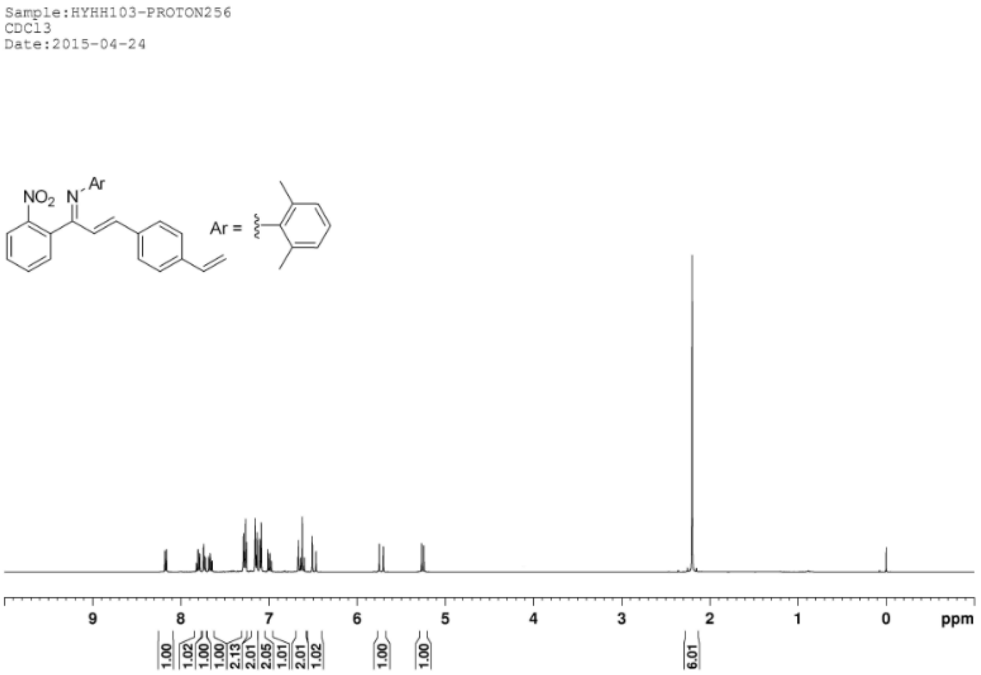

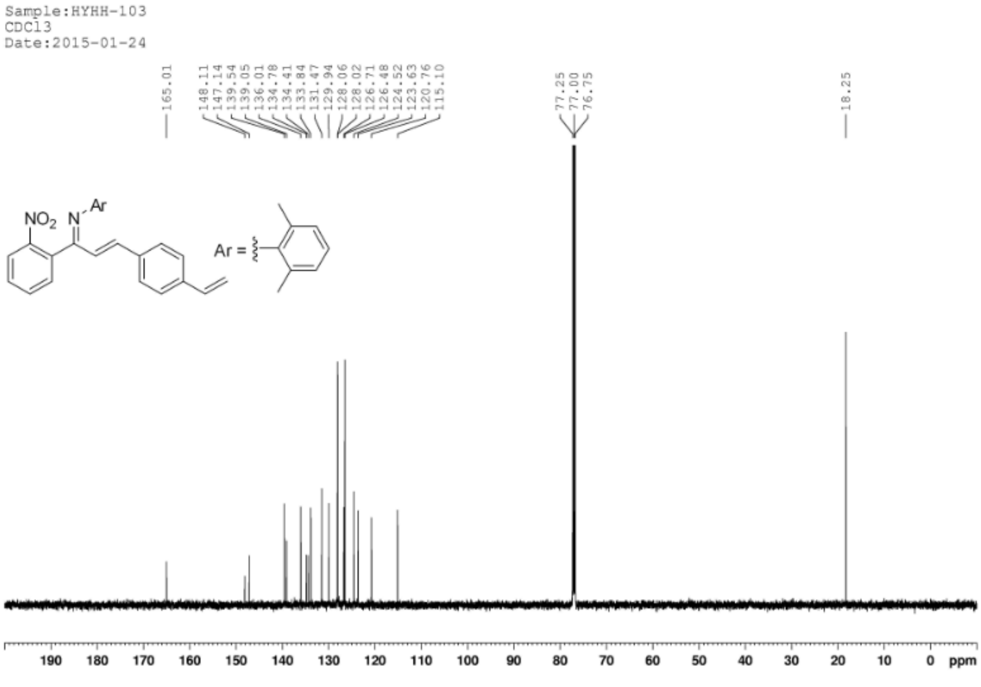

Supplementary Figure 30. ^{1}H NMR (500 MHz) and ^{13}C NMR (125 MHz) spectra of **2ad** ($CDCl_3$)

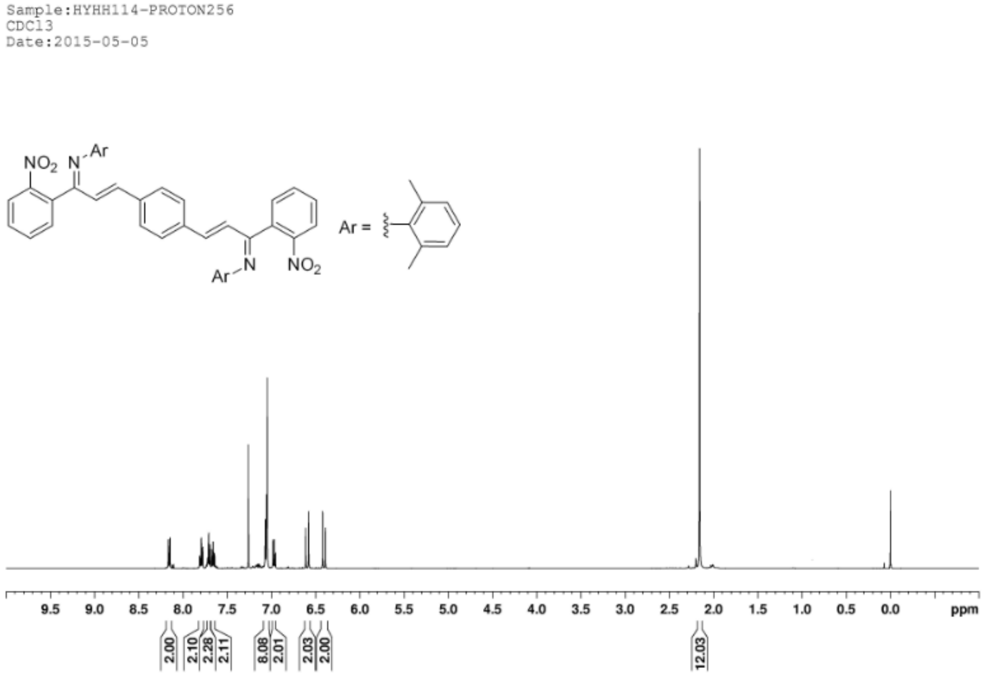

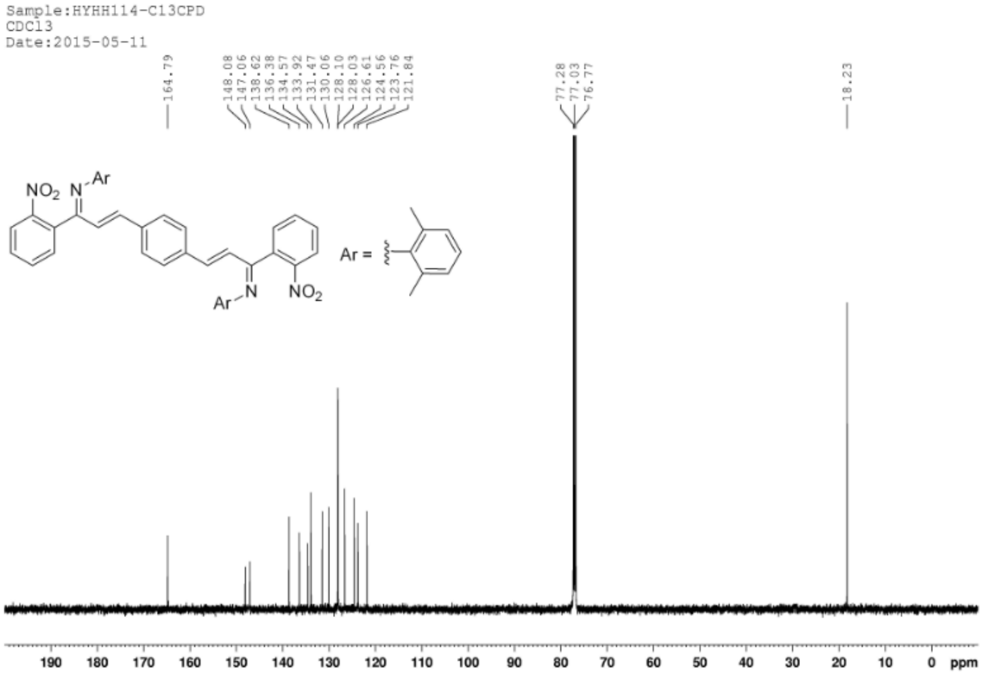

Supplementary Figure 31. ^{1}H NMR (500 MHz) and ^{13}C NMR (125 MHz) spectra of **2ae** ($CDCl_3$)

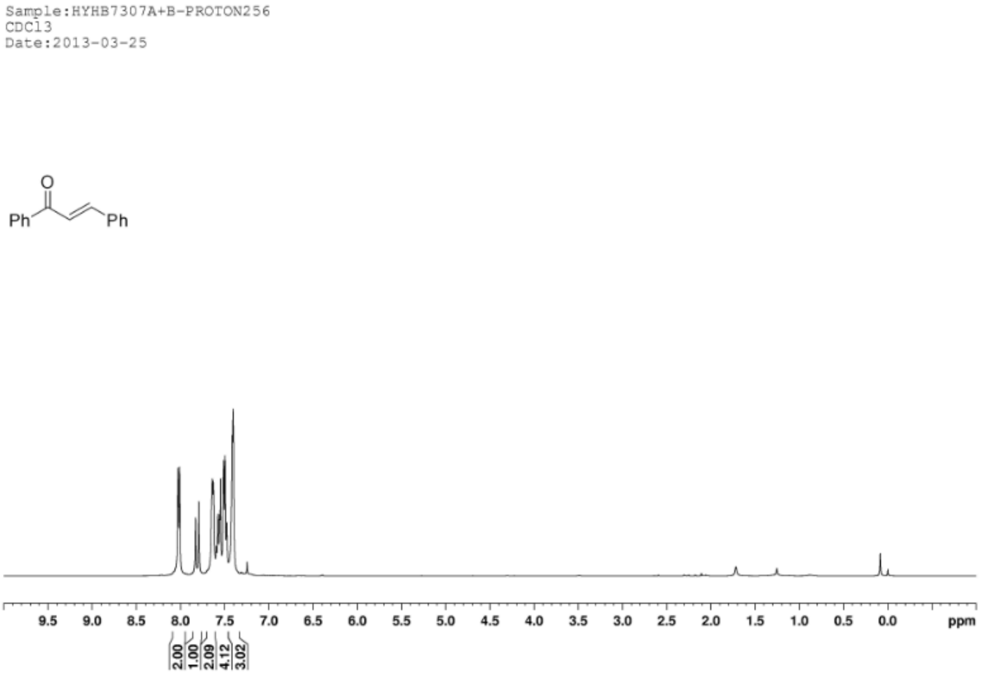

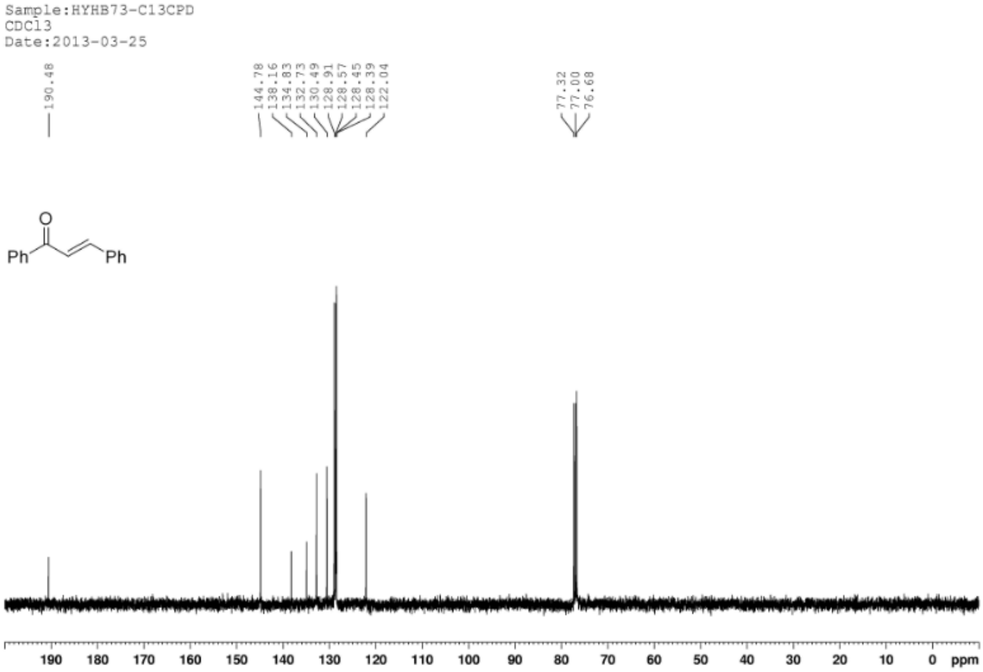

Supplementary Figure 32. ^{1}H NMR (400 MHz) and ^{13}C NMR (100 MHz) spectra of **3a** ($CDCl_3$)

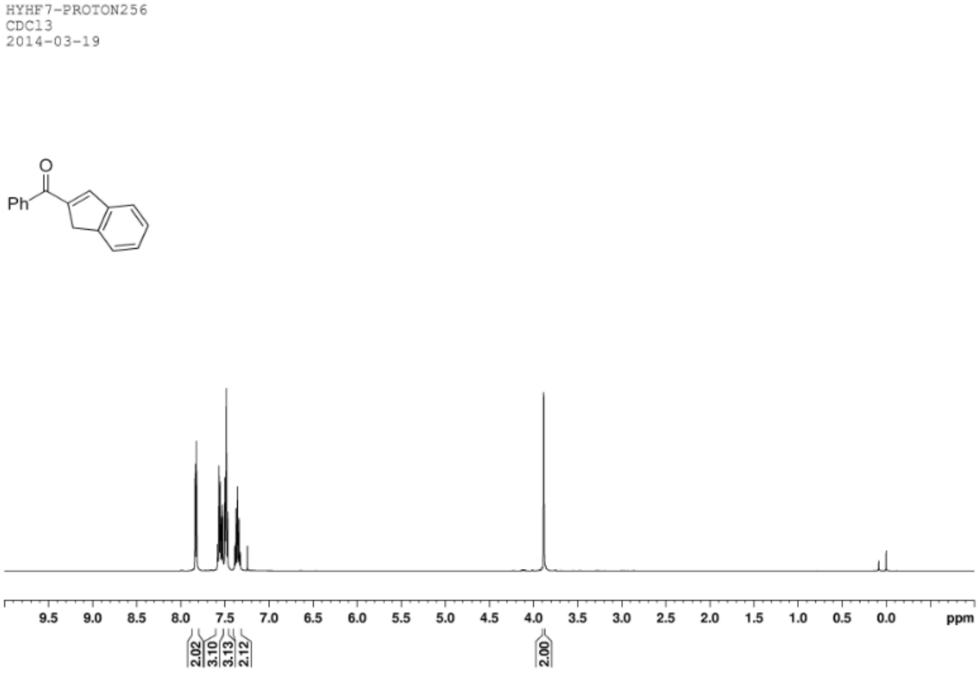

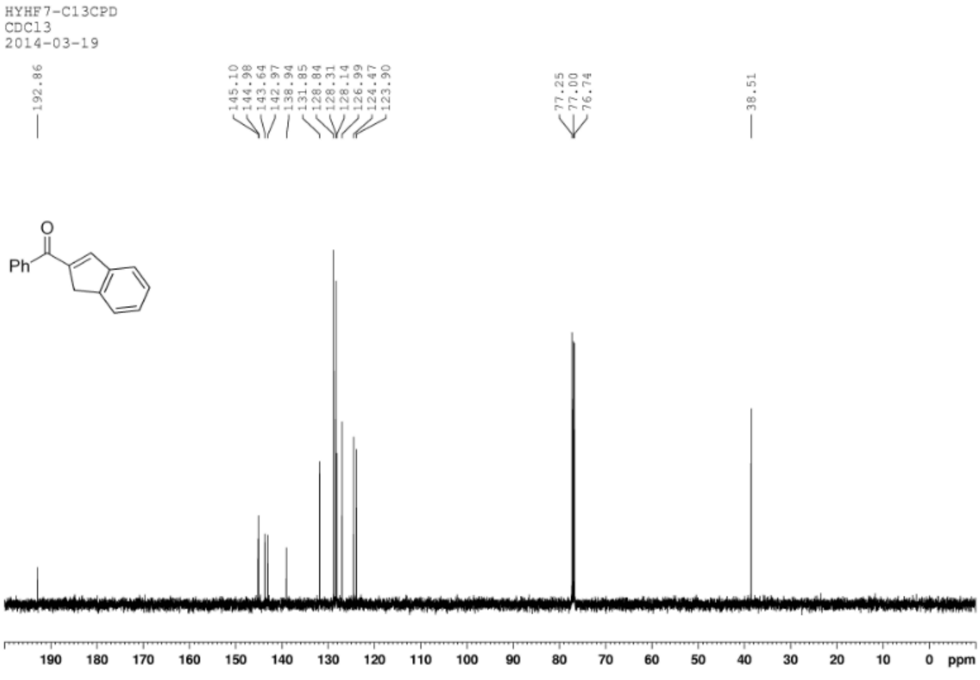

Supplementary Figure 33. ^{1}H NMR (400 MHz) and ^{13}C NMR (100 MHz) spectra of **3b** ($CDCl_3$)

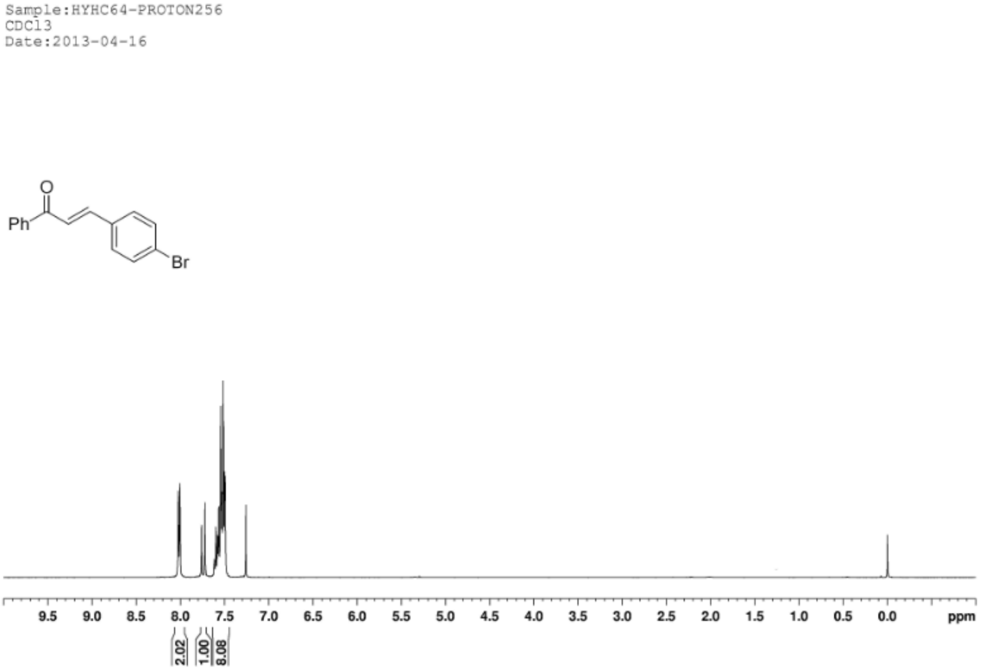

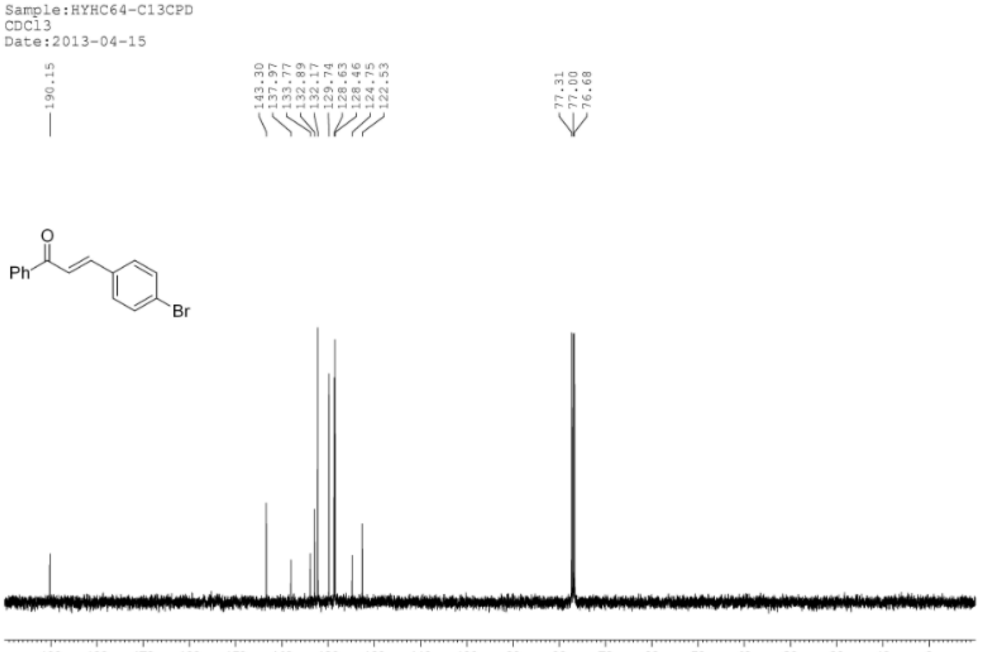

Supplementary Figure 34. ^{1}H NMR (400 MHz) and ^{13}C NMR (100 MHz) spectra of **3c** ($CDCl_3$)

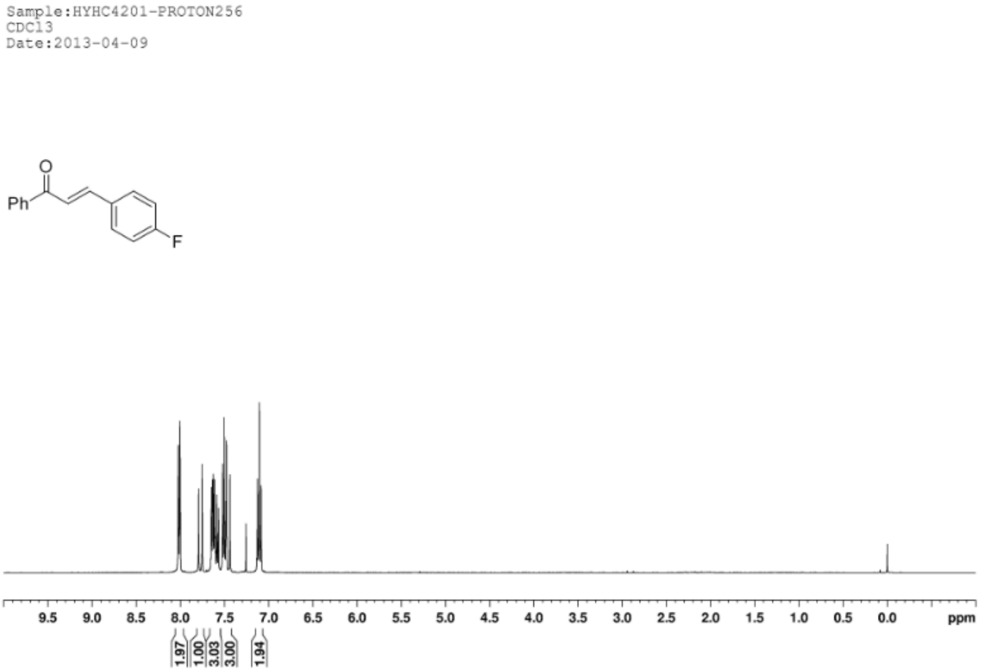

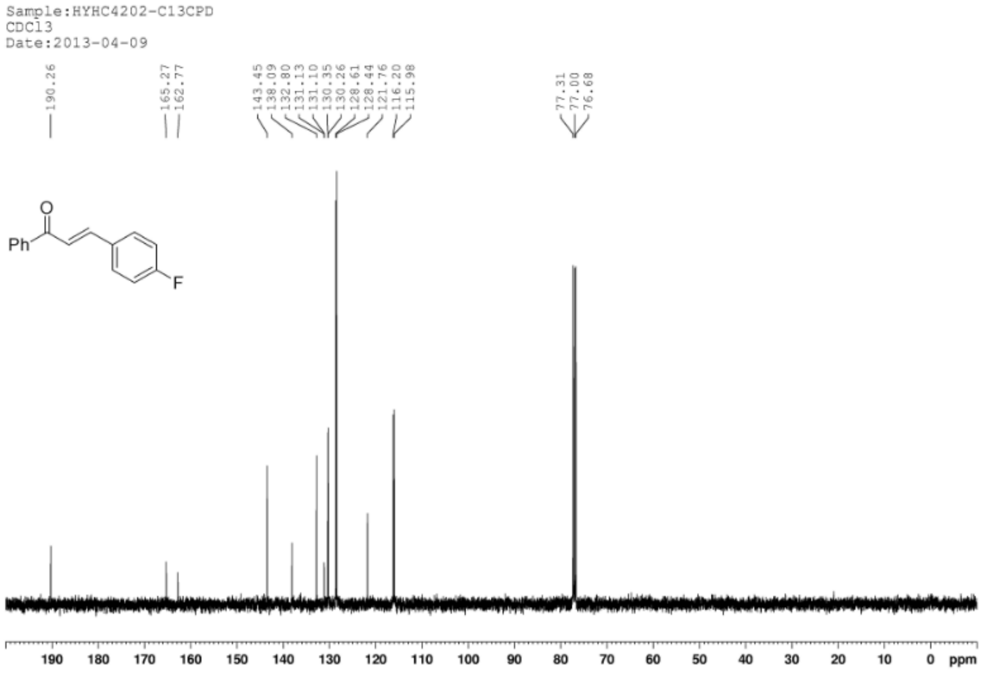

Supplementary Figure 35. ^{1}H NMR (400 MHz) and ^{13}C NMR (100 MHz) spectra of **3d** ($CDCl_3$)

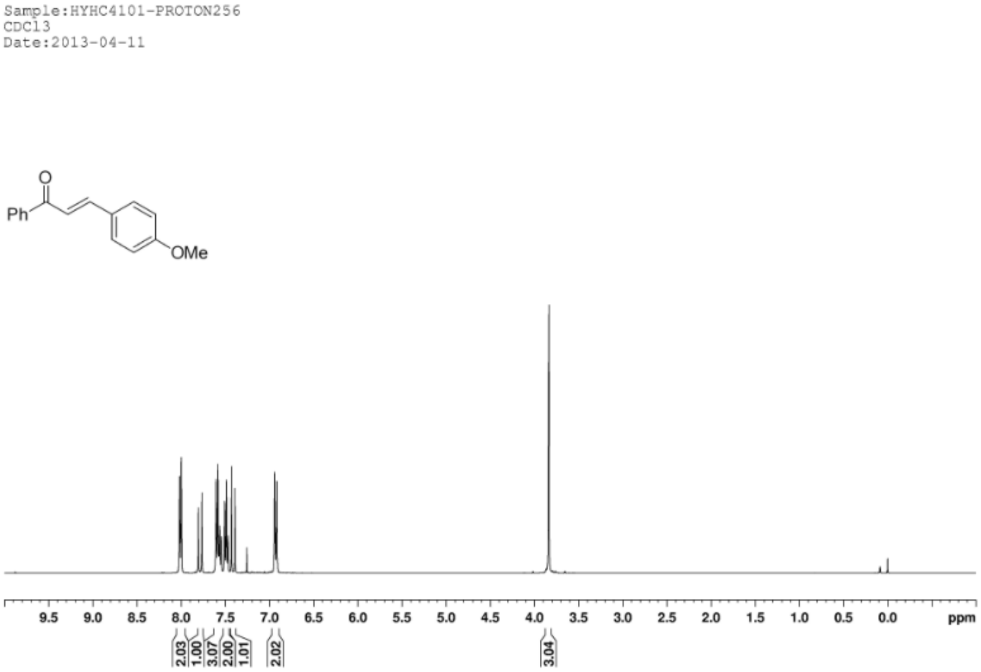

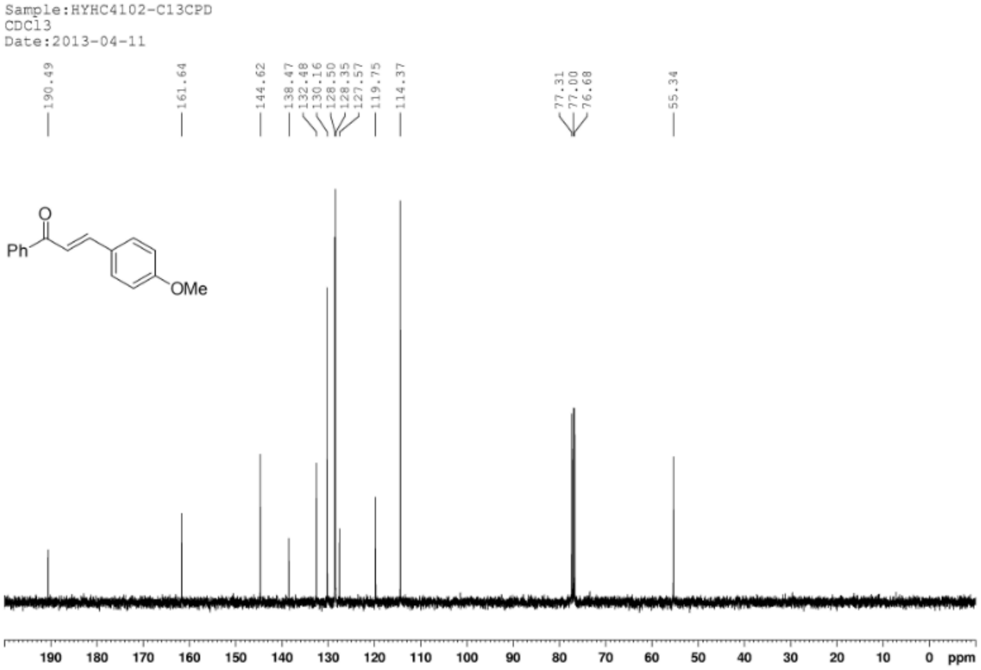

Supplementary Figure 36. ^{1}H NMR (400 MHz) and ^{13}C NMR (100 MHz) spectra of **3e** ($CDCl_3$)

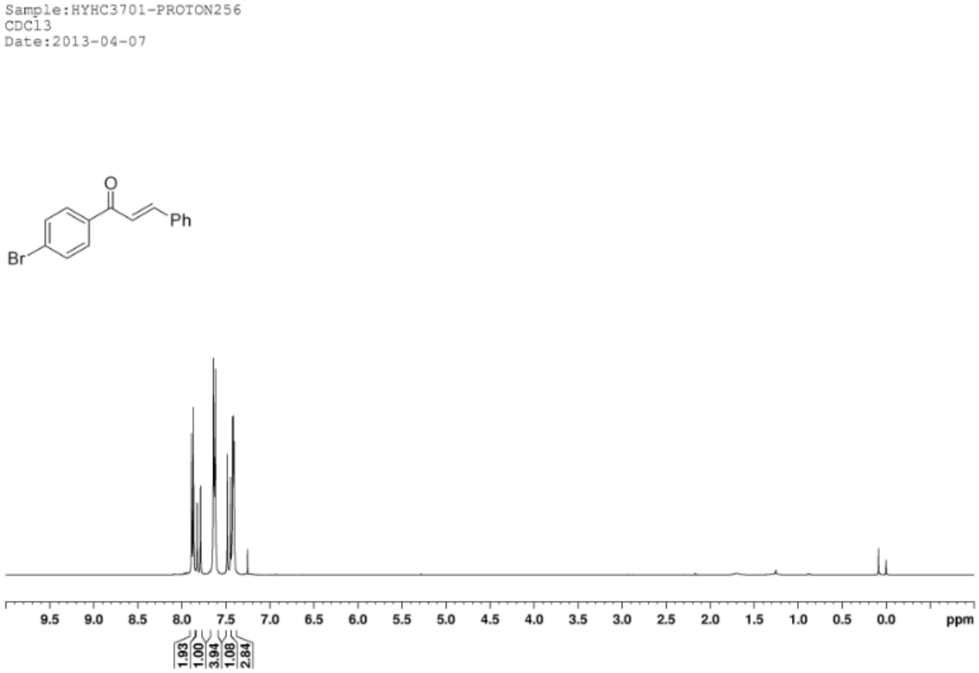

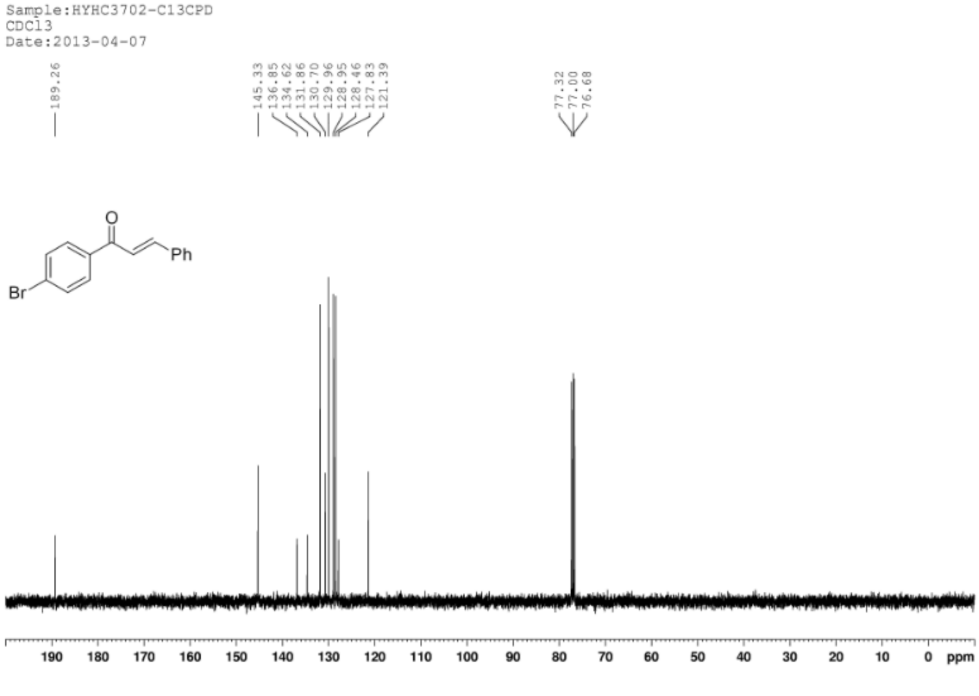

Supplementary Figure 37. ^{1}H NMR (400 MHz) and ^{13}C NMR (100 MHz) spectra of **3f** ($CDCl_3$)

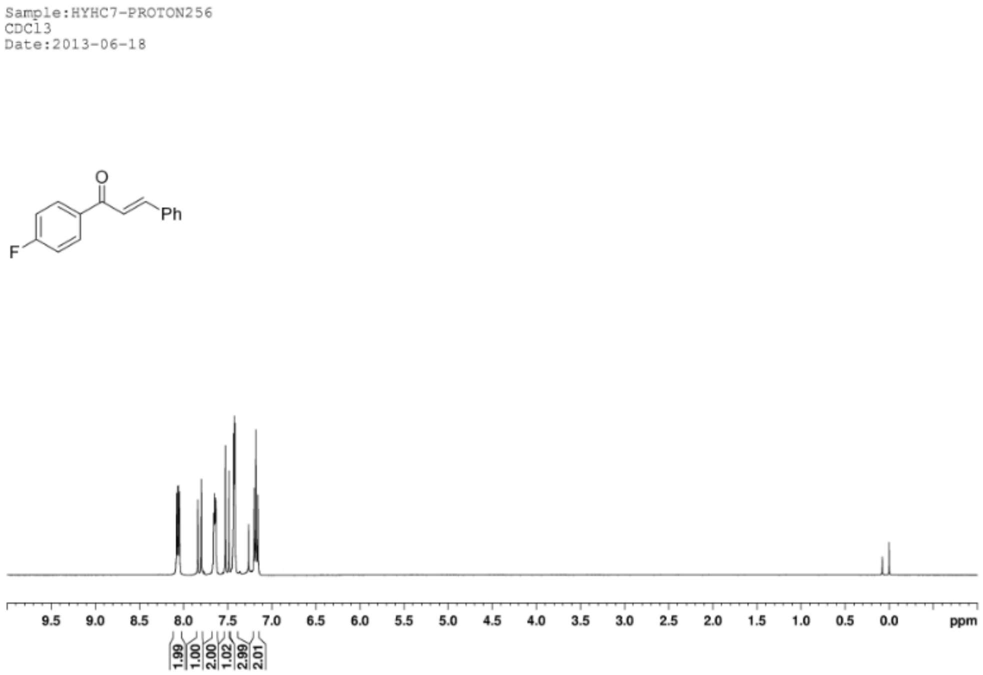

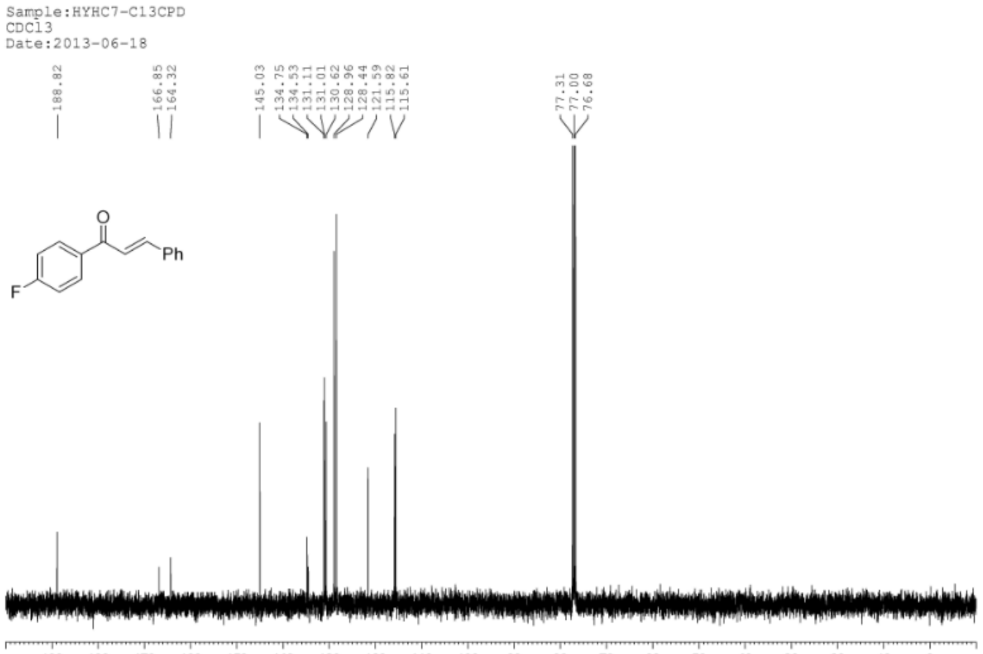

Supplementary Figure 38. ^{1}H NMR (400 MHz) and ^{13}C NMR (100 MHz) spectra of **3g** ($CDCl_3$)

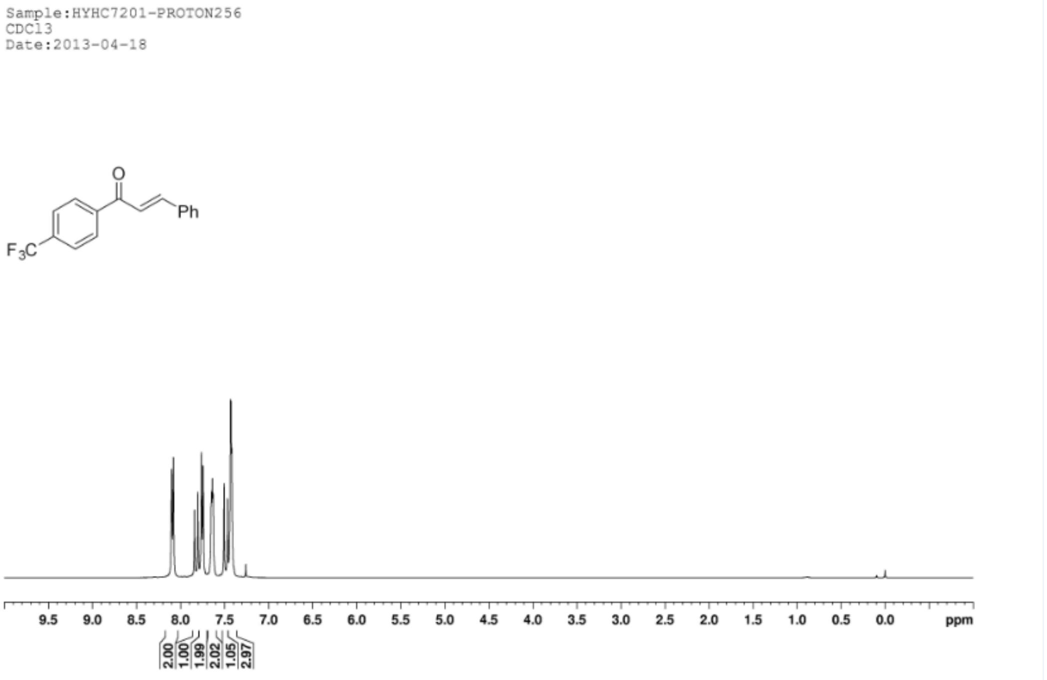

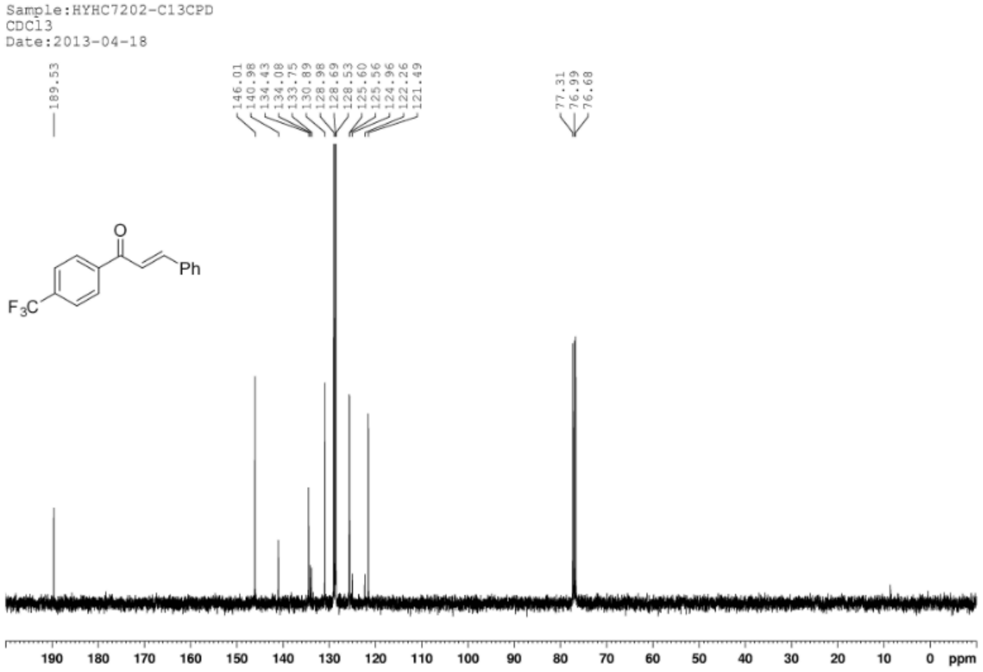

Supplementary Figure 39. ^{1}H NMR (400 MHz) and ^{13}C NMR (100 MHz) spectra of **3h** ($CDCl_3$)

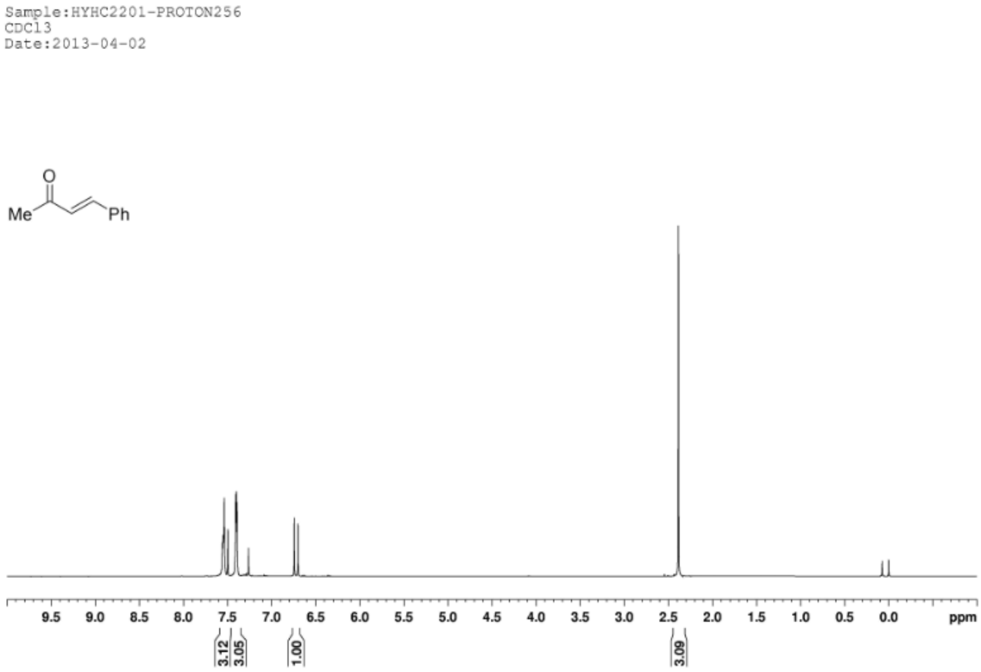

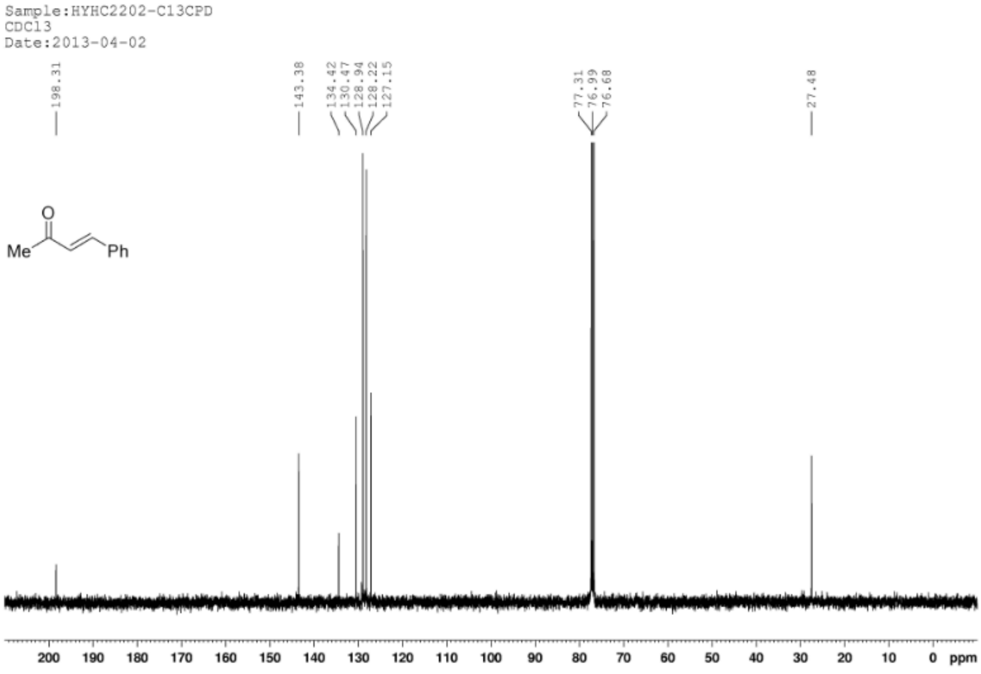

Supplementary Figure 40. ^{1}H NMR (400 MHz) and ^{13}C NMR (100 MHz) spectra of **3i** ($CDCl_3$)

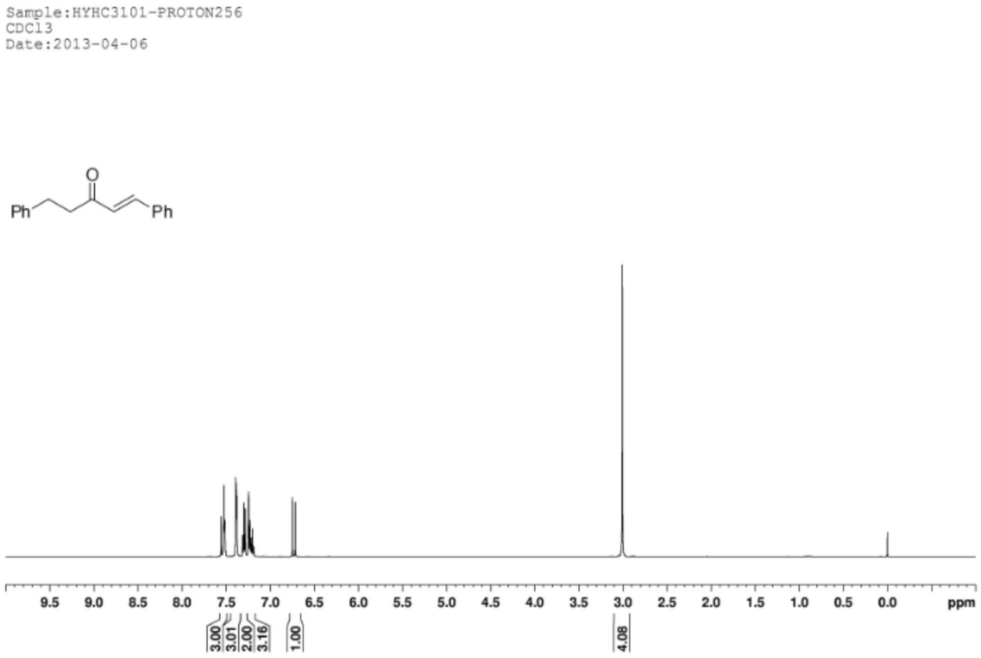

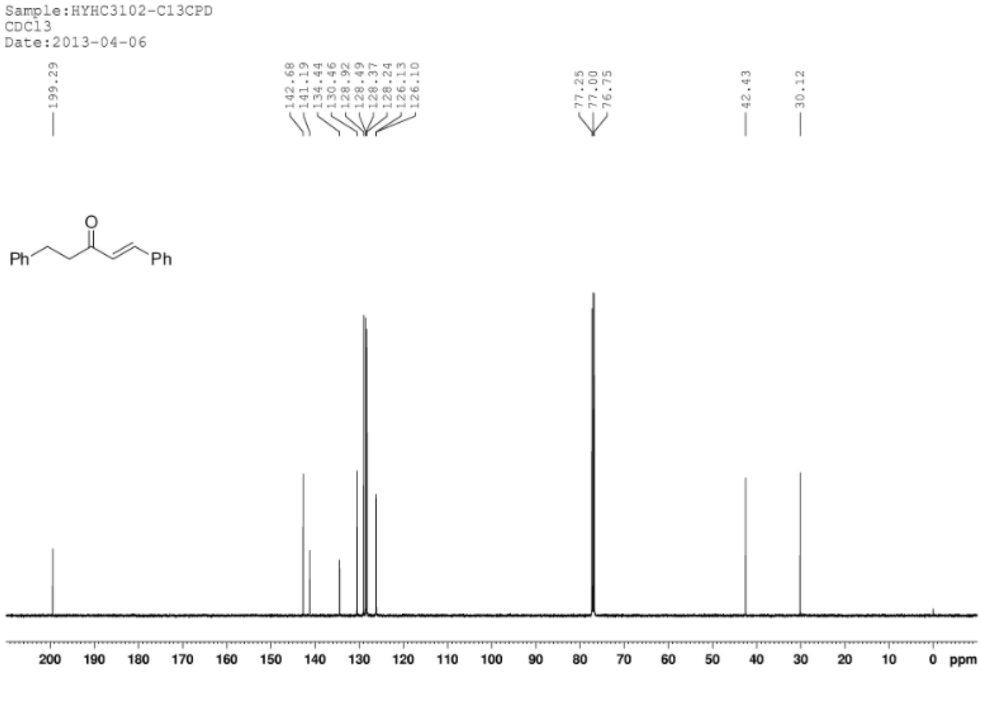

Supplementary Figure 41. ^{1}H NMR (500 MHz) and ^{13}C NMR (125 MHz) spectra of **3j** ($CDCl_3$)

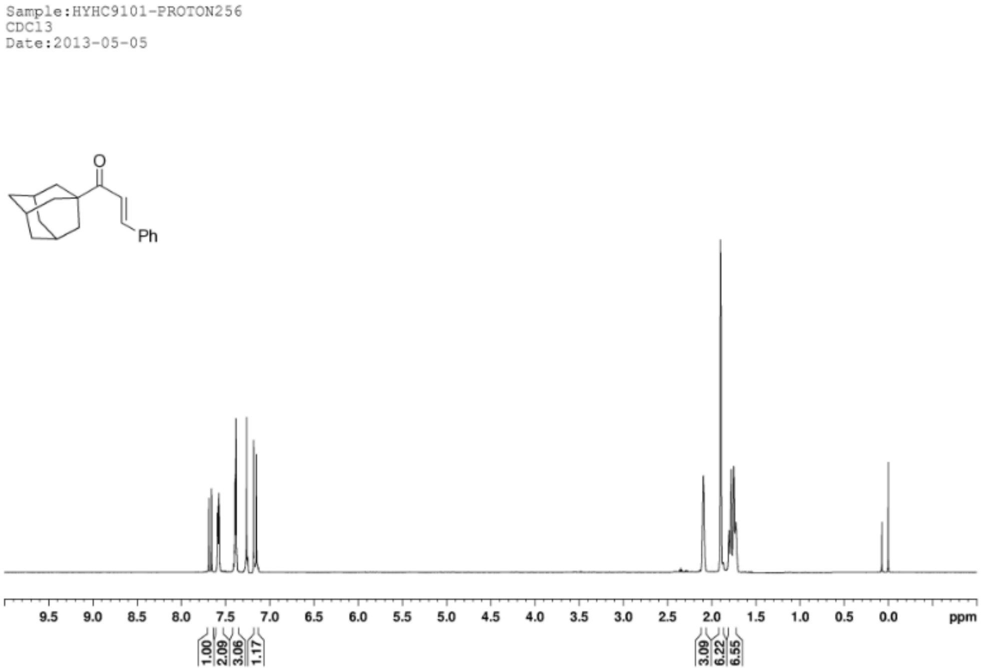

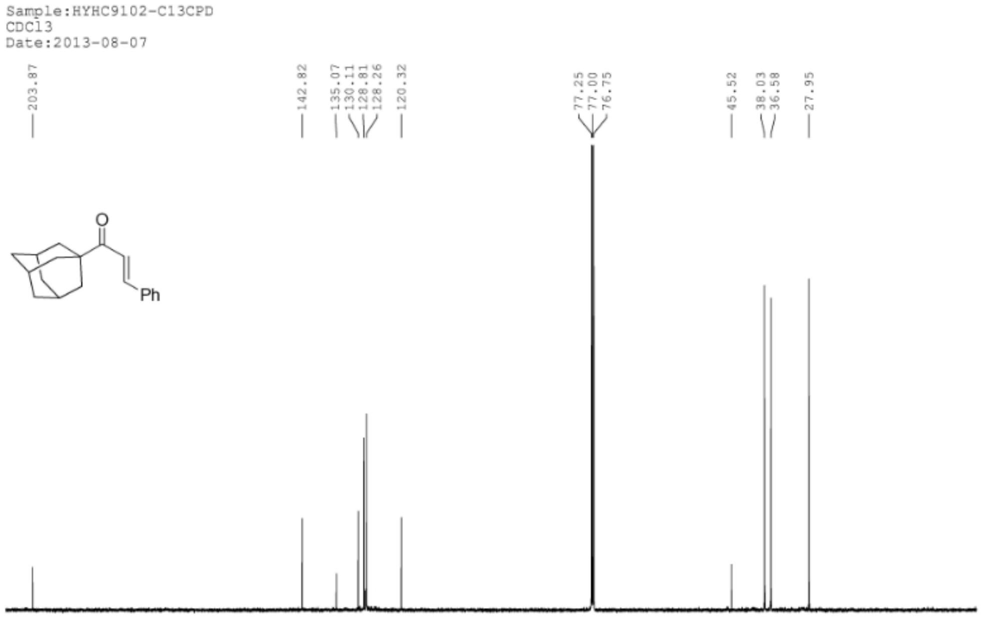

Supplementary Figure 42. ^{1}H NMR (500 MHz) and ^{13}C NMR (125 MHz) spectra of **3k** ($CDCl_3$)

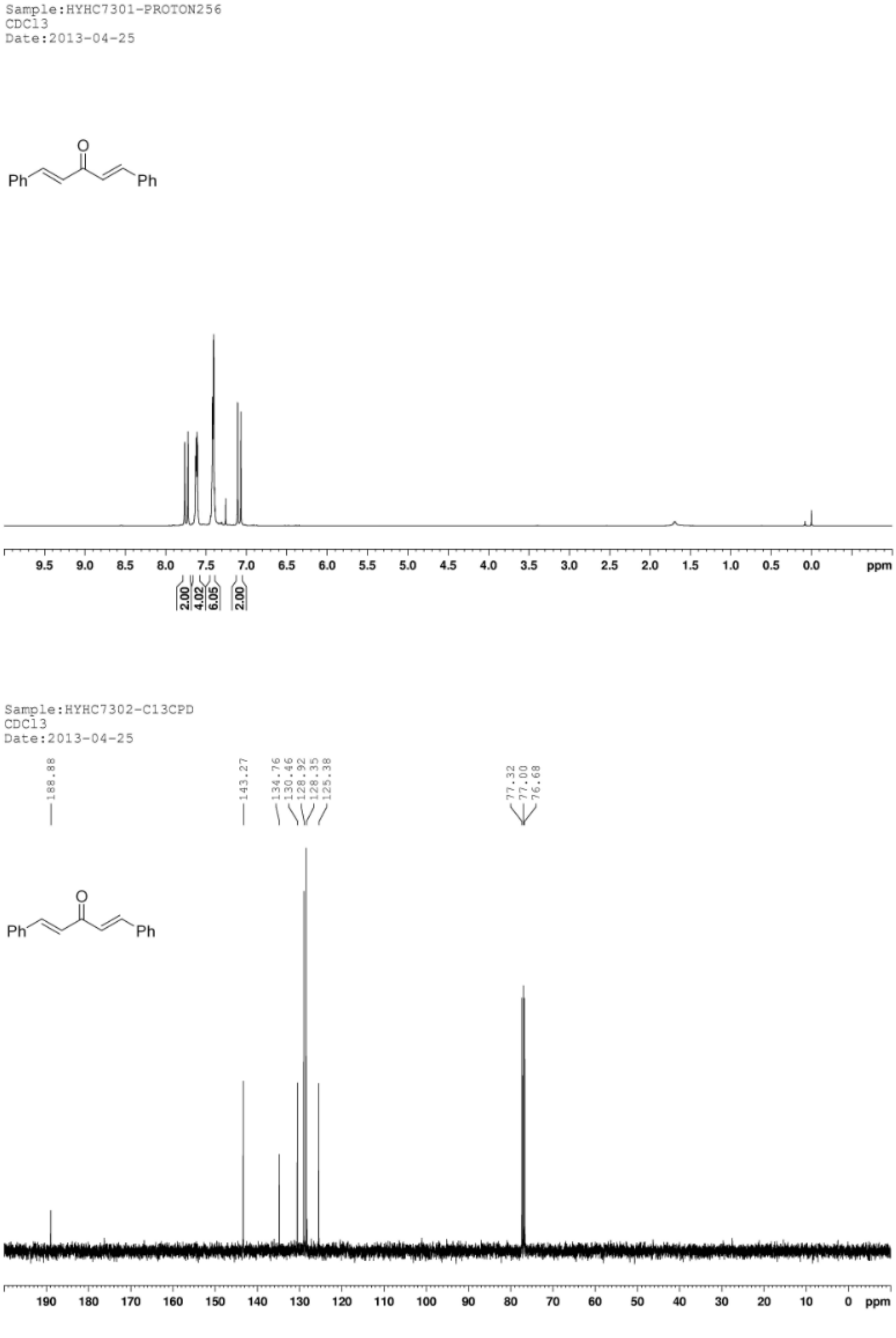

Supplementary Figure 43. ^{1}H NMR (400 MHz) and ^{13}C NMR (100 MHz) spectra of **3l** ($CDCl_3$)

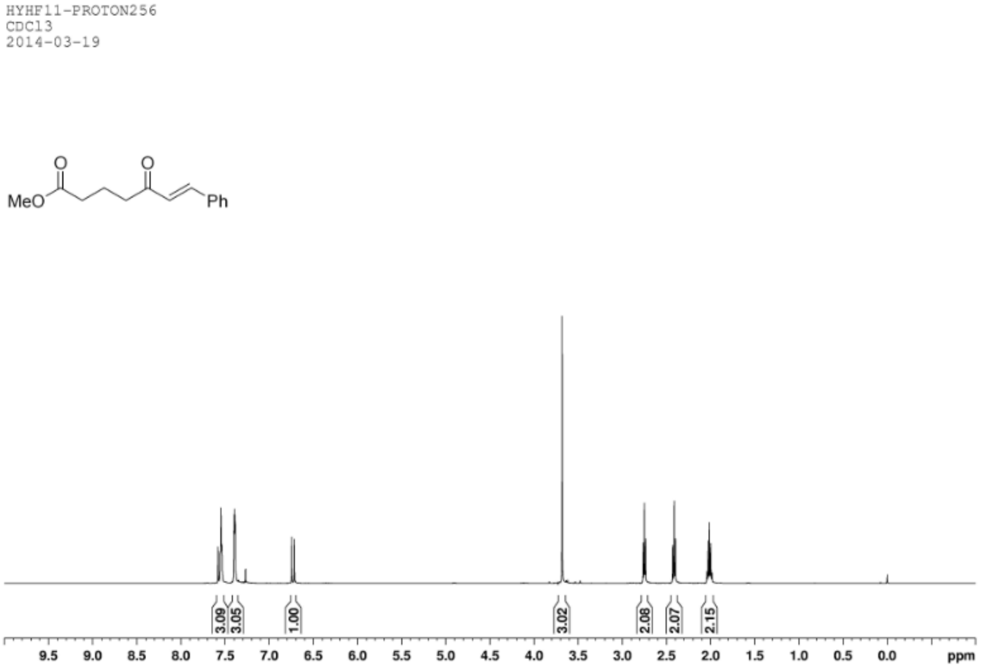

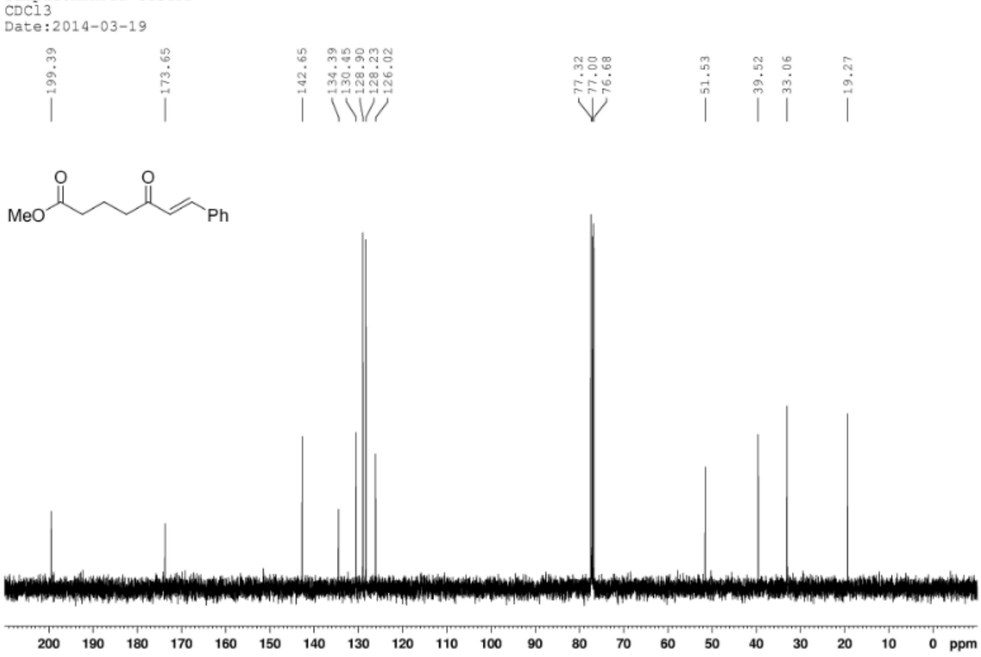

Supplementary Figure 44. ^{1}H NMR (400 MHz) and ^{13}C NMR (100 MHz) spectra of **3m** ($CDCl_3$)

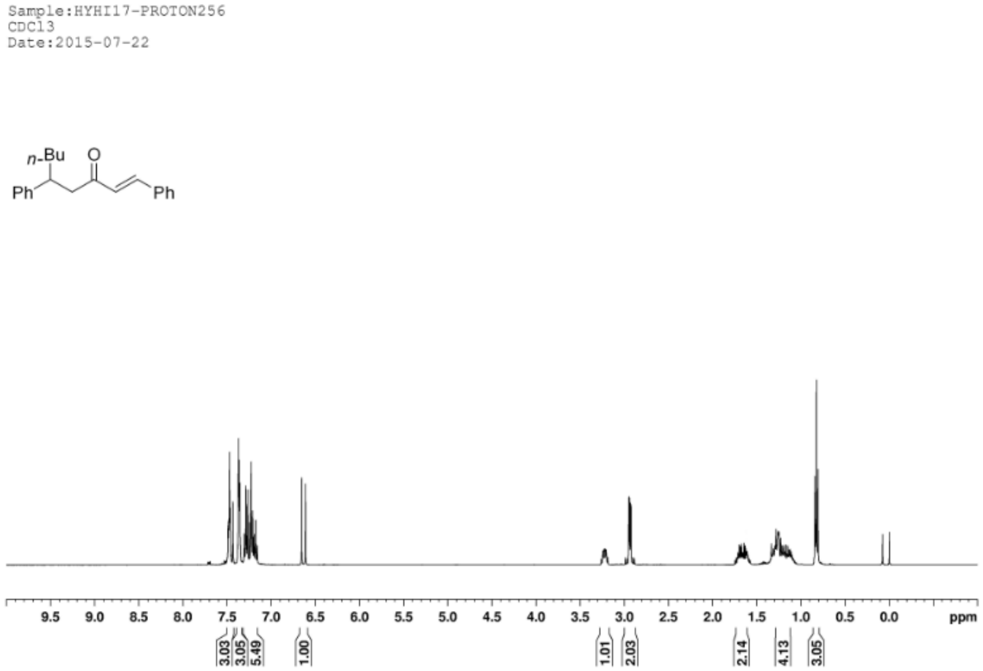

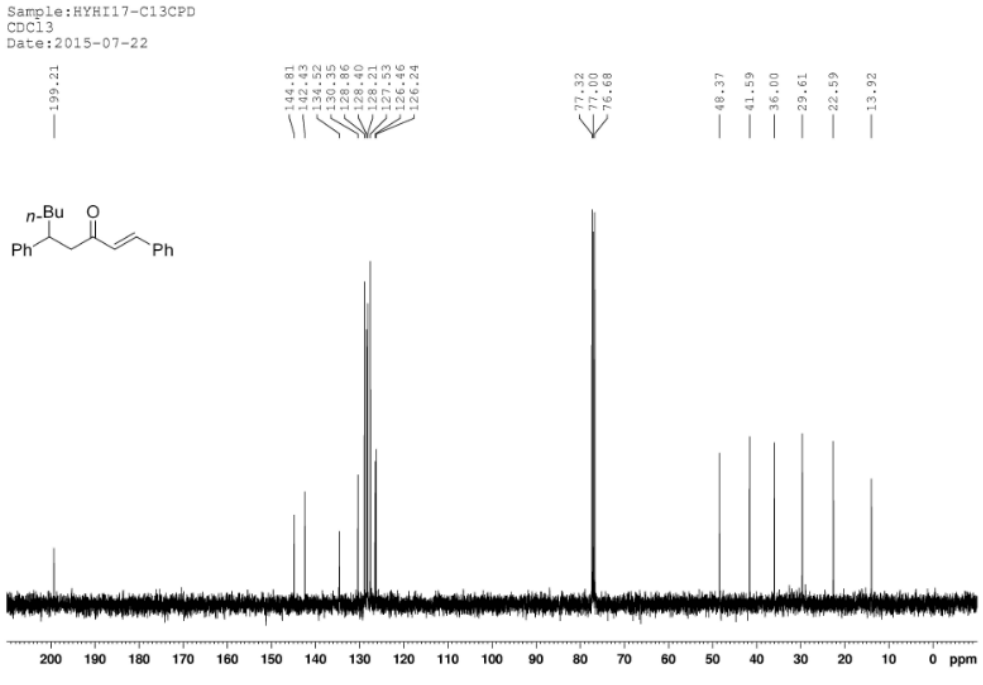

Supplementary Figure 45. ^{1}H NMR (400 MHz) and ^{13}C NMR (100 MHz) spectra of **3n** ($CDCl_3$)

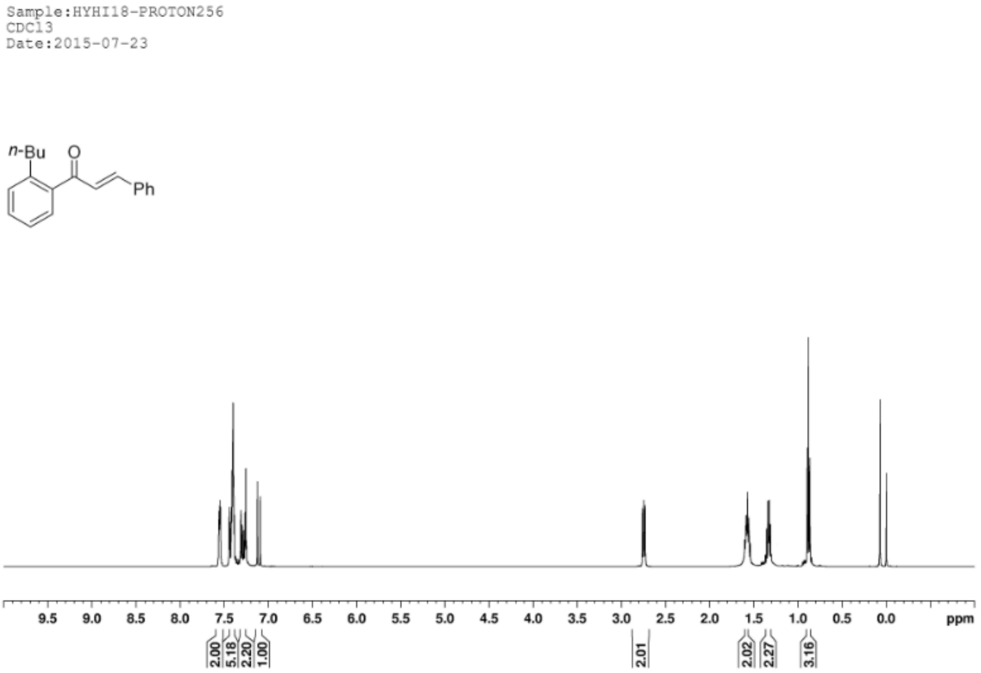

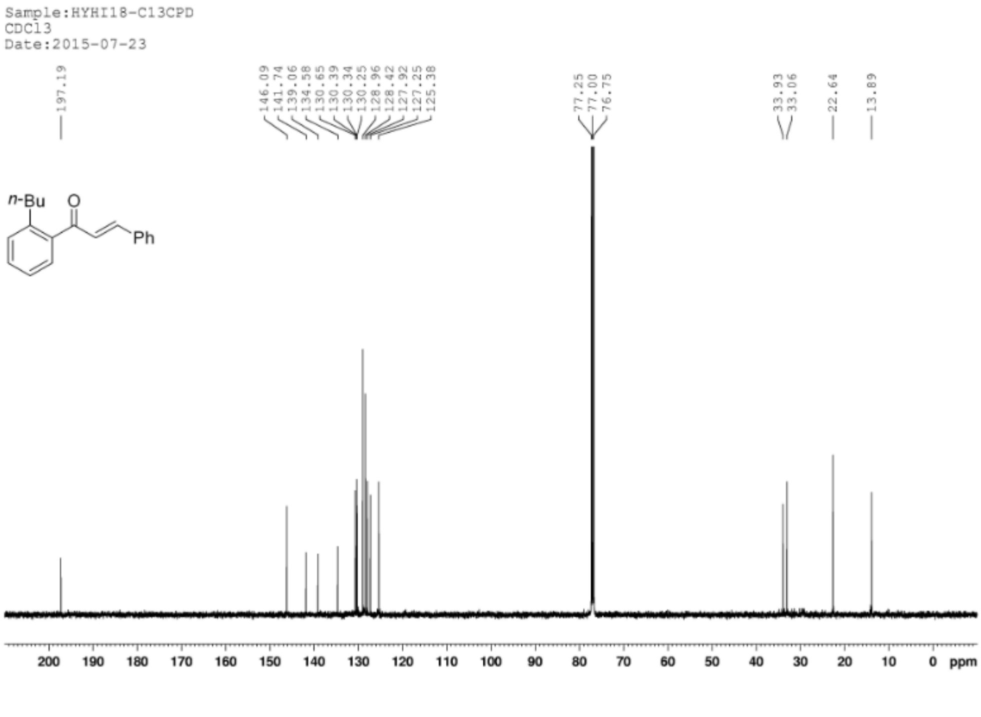

Supplementary Figure 46. ^{1}H NMR (500 MHz) and ^{13}C NMR (125 MHz) spectra of **3o** ($CDCl_3$)

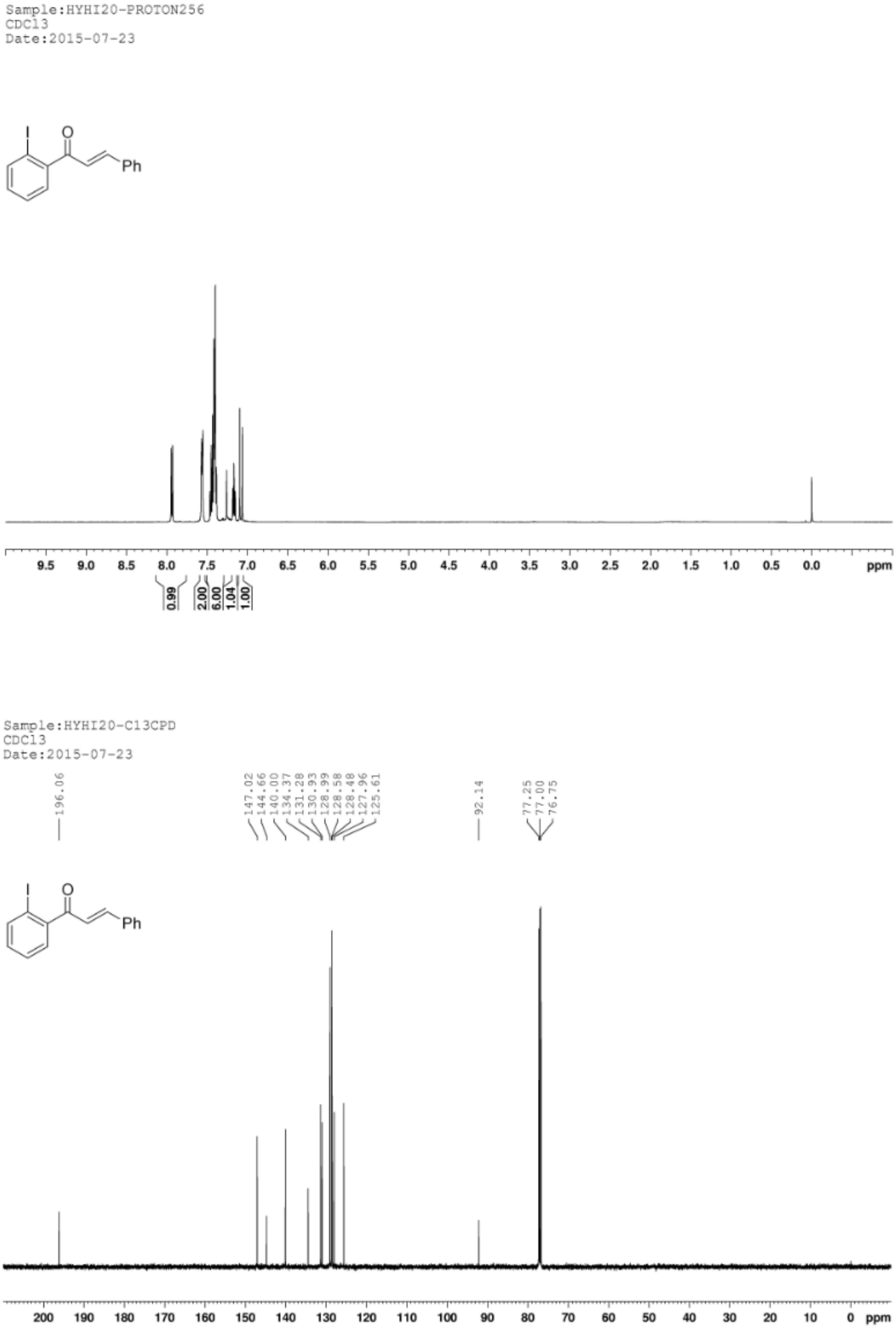

Supplementary Figure 47. ^{1}H NMR (500 MHz) and ^{13}C NMR (125 MHz) spectra of **3p** ($CDCl_3$)

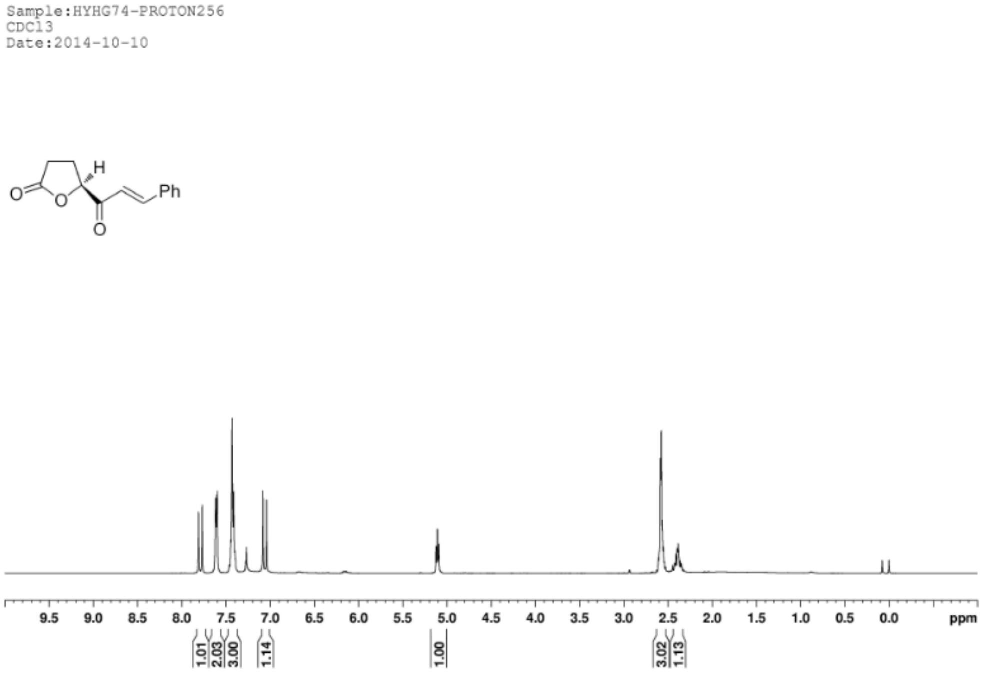

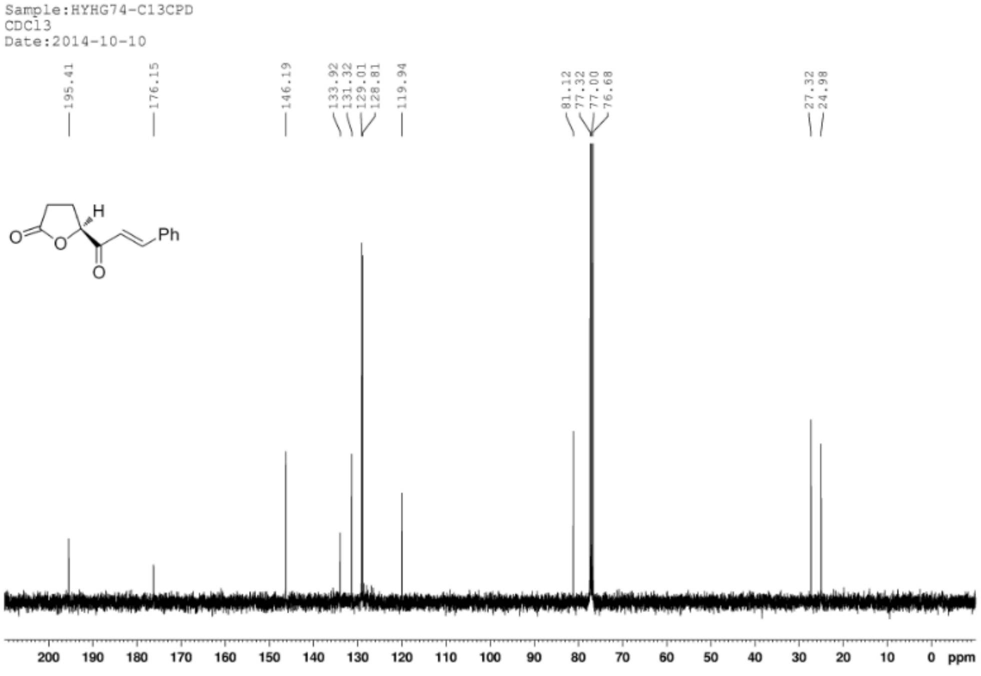

Supplementary Figure 48. ^{1}H NMR (400 MHz) and ^{13}C NMR (100 MHz) spectra of **3q** ($CDCl_3$)

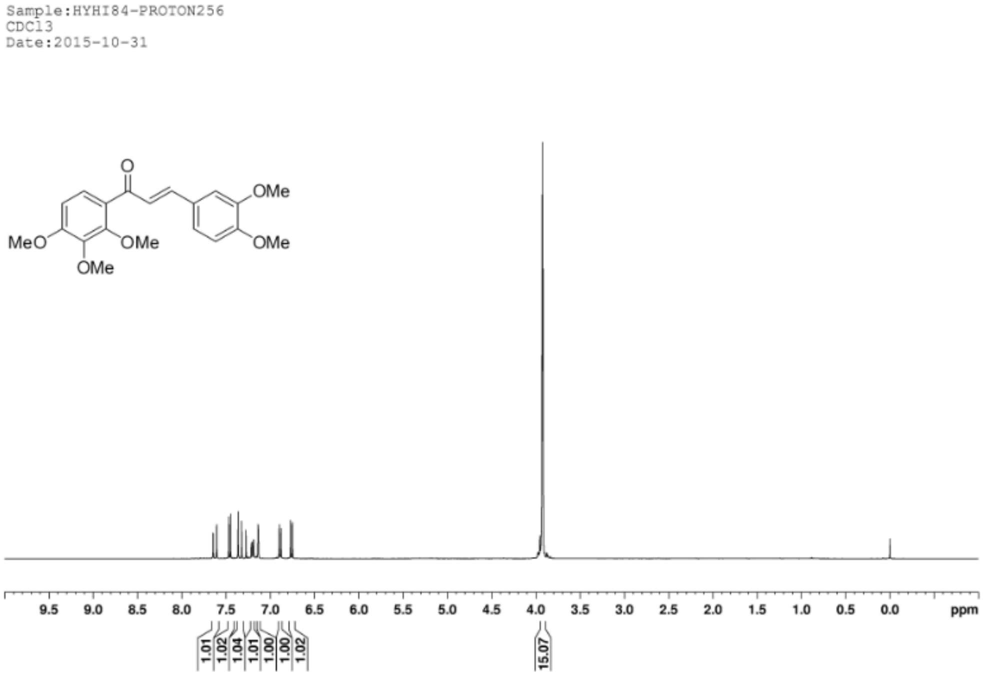

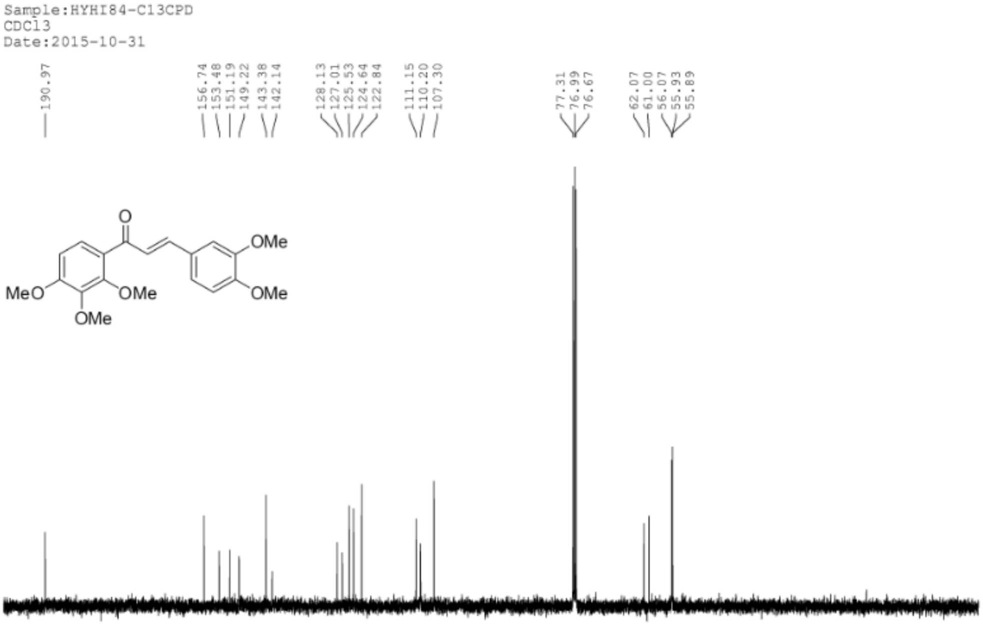

Supplementary Figure 49. ^{1}H NMR (400 MHz) and ^{13}C NMR (100 MHz) spectra of **3r** ($CDCl_3$)

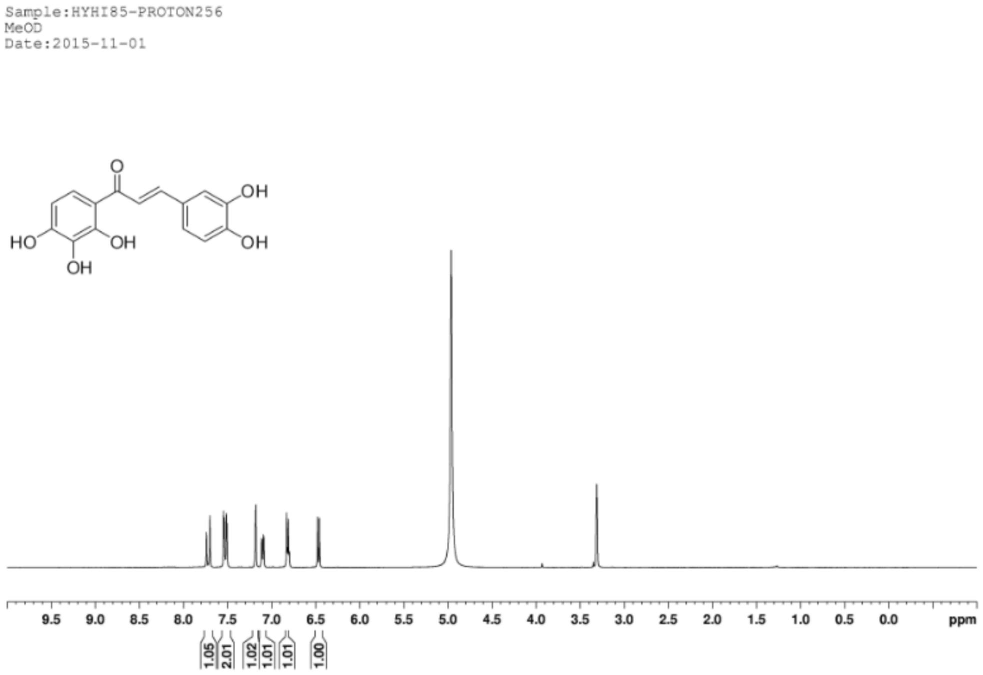

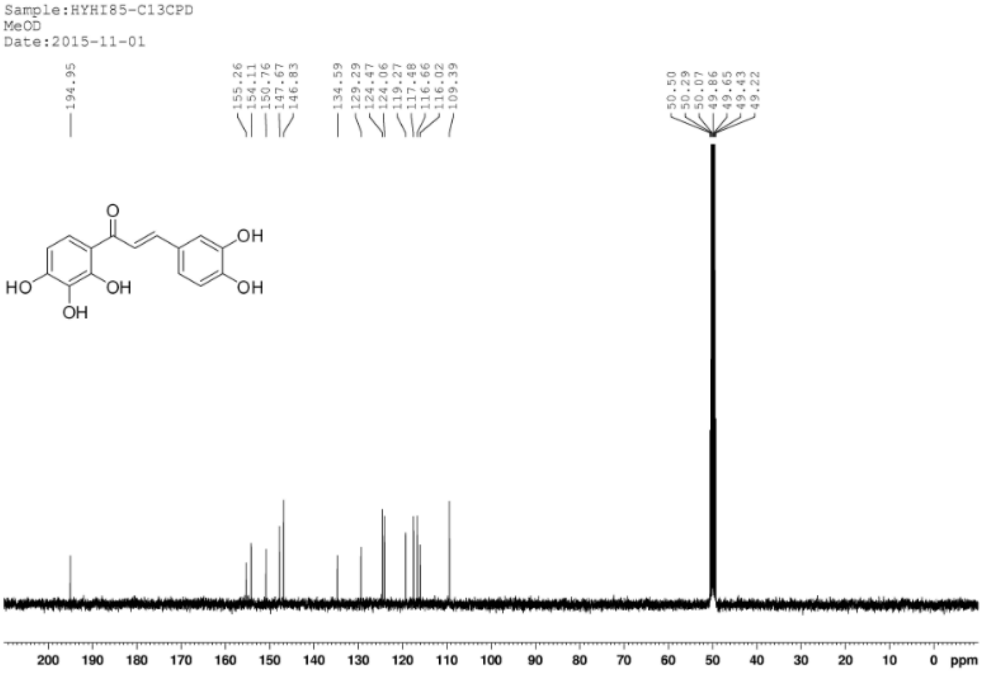

Supplementary Figure 50. ^{1}H NMR (500 MHz) and ^{13}C NMR (125 MHz) spectra of **4** (CD_3OD)

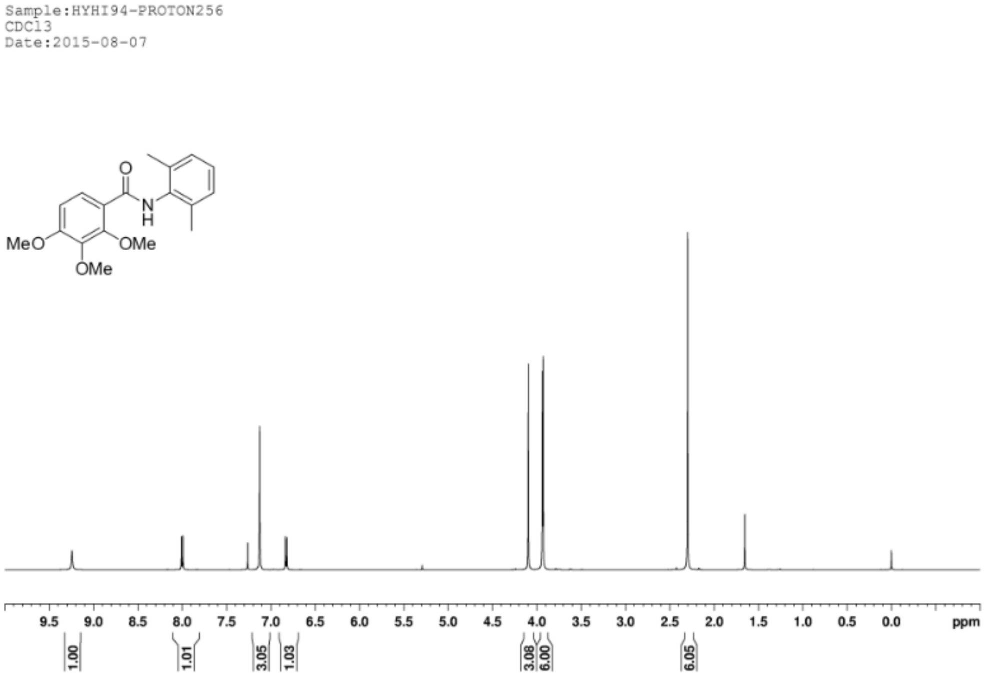

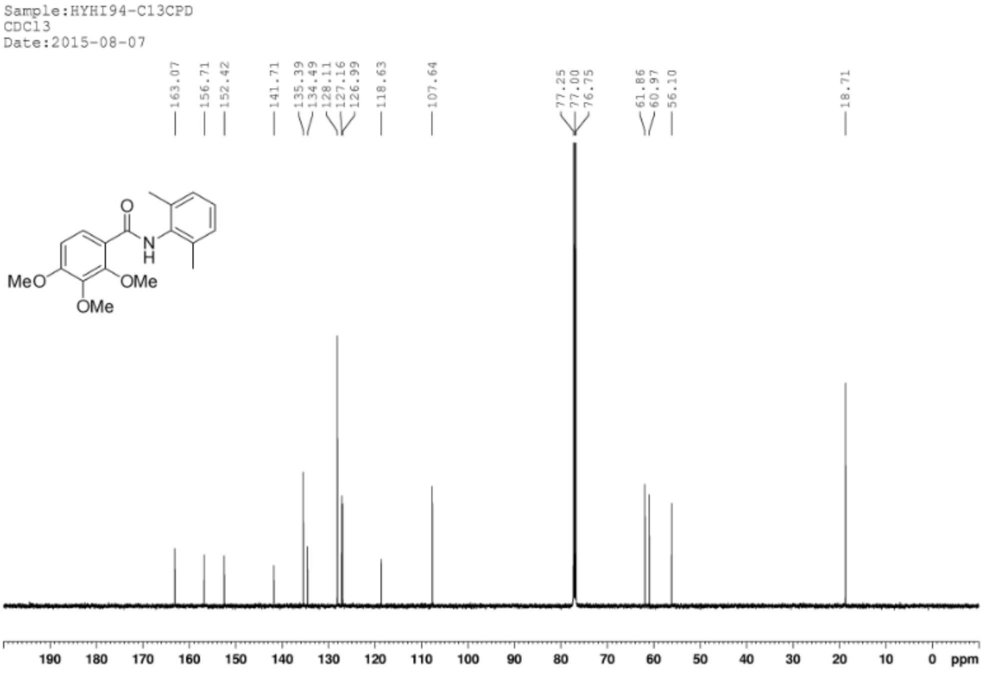

Supplementary Figure 51. ^{1}H NMR (500 MHz) and ^{13}C NMR (125 MHz) spectra of **1x** ($CDCl_3$)

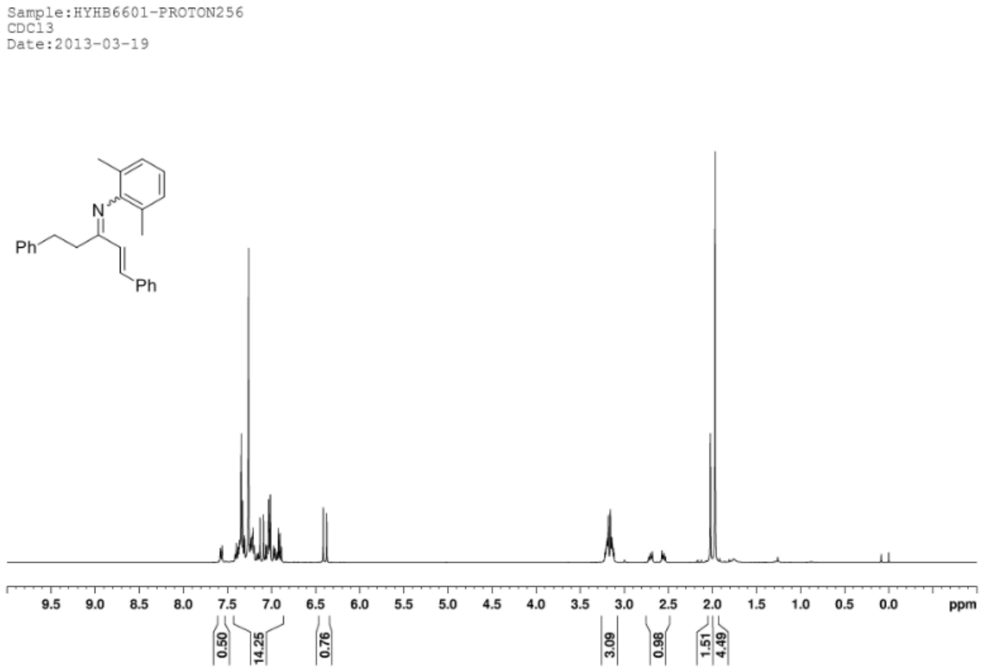

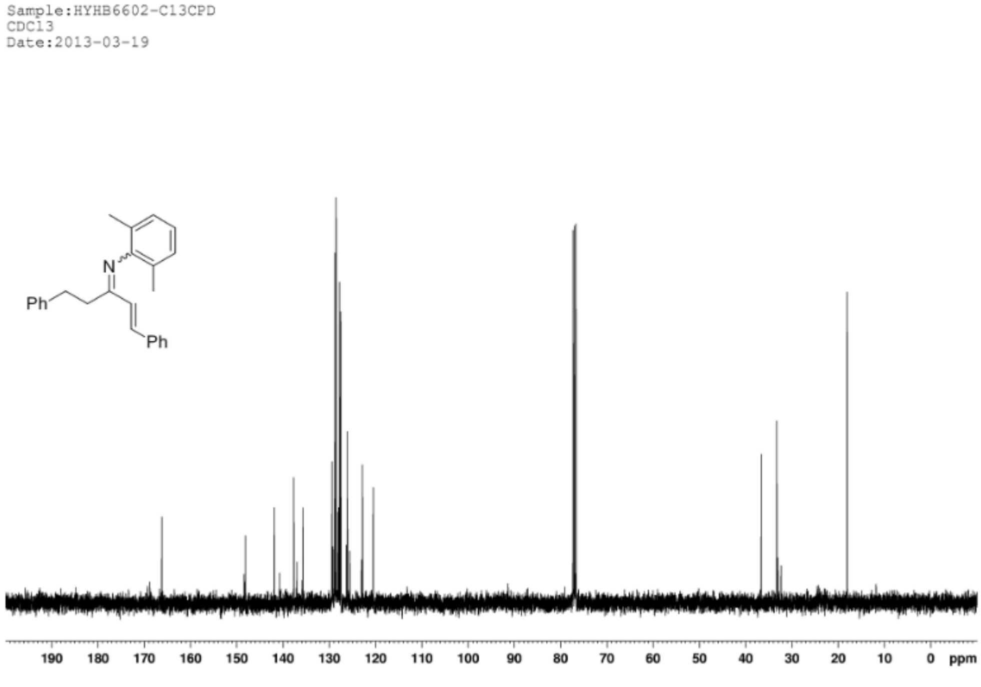

Supplementary Figure 52. ^{1}H NMR (400 MHz) and ^{13}C NMR (100 MHz) spectra of **2af** ($CDCl_3$)

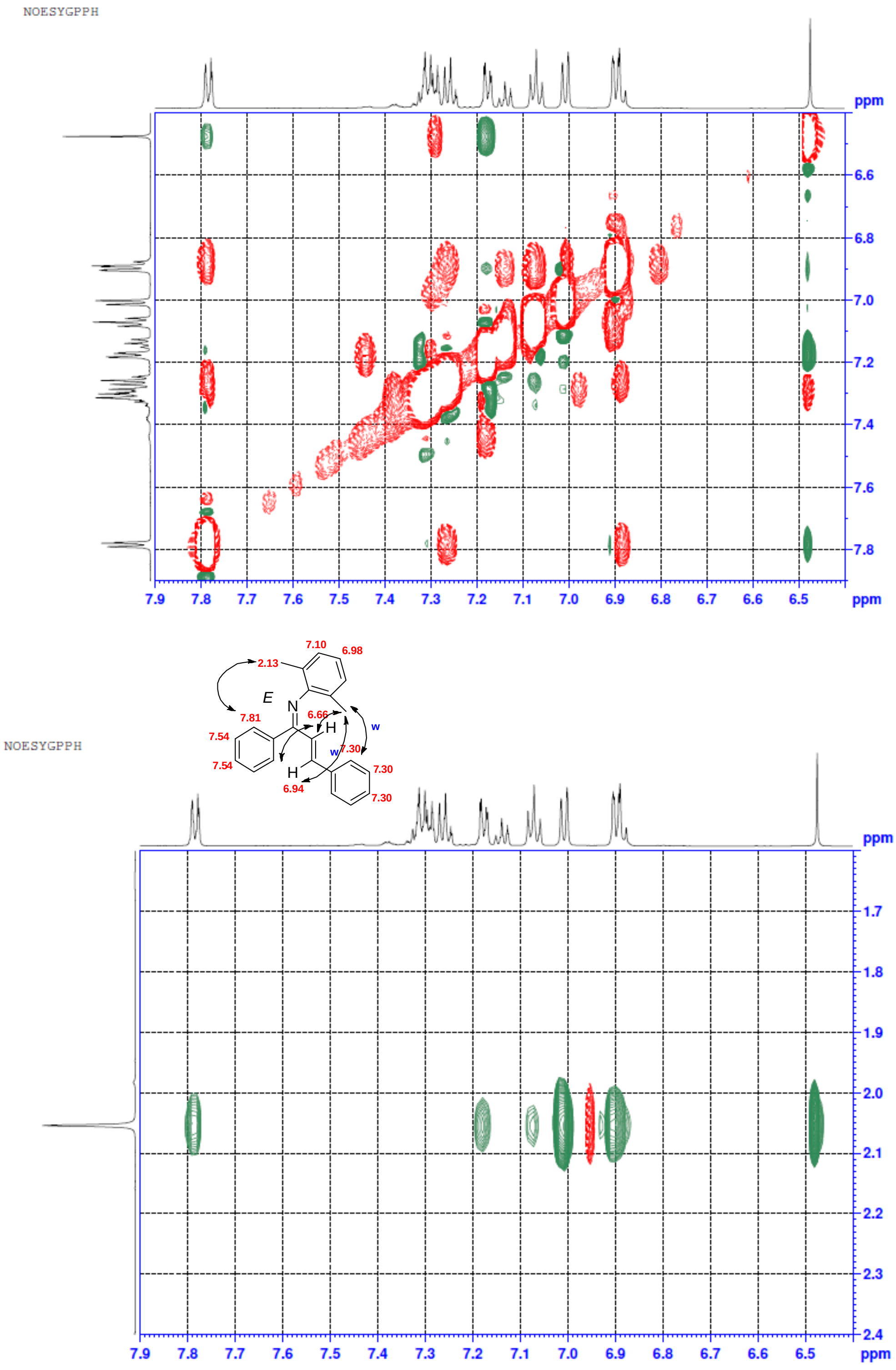

Supplementary Figure 53. NOESY spectra of α,β-unsaturated ketimine **2a.**

Crystallographic data for ketimine 2w and X-ray structure (CCDC 1438540)

Bond precision:	C-C = 0.0036 A	Wavelength=0.71073	
Cell:	a=25.692 (3)	b=11.5912 (11)	c=18.7420 (19)
	alpha=90	beta=129.883 (10)	gamma=90
Temperature:	187 K		

	Calculated	Reported
Volume	4282.9 (10)	4282.9 (7)
Space group	C 2/c	C 2/c
Hall group	-C 2yc	?
Moiety formula	C25 H22 N2 O4	?
Sum formula	C25 H22 N2 O4	C25 H22 N2 O4
Mr	414.45	414.45
Dx,g cm-3	1.286	1.285
Z	8	8
Mu (mm-1)	0.088	0.088
F000	1744.0	1744.0
F000’	1744.82	
h,k,lmax	35,15,25	32,15,25
Nref	5787	4997
Tmin,Tmax	0.991,0.996	0.991,0.996
Tmin’	0.991	

Correction method= MULTI-SCAN

Data completeness= 0.863 Theta(max)= 29.170

R(reflections)= 0.0637(3208) wR2(reflections)= 0.1524(4997)

S = 1.018 Npar= 280

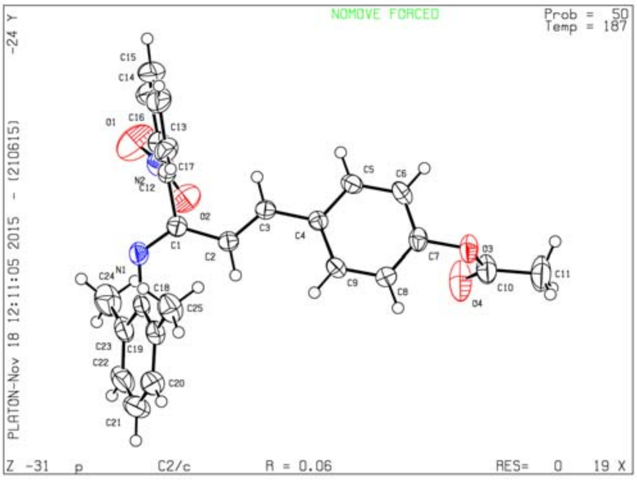

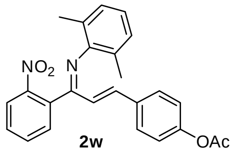

Supplementary Figure 54. Ortep structure of ketimine 2w. (CCDC 1438540)

Shimadzu CLASS-VP V6.13 SP2 ***Area % Report***
Column: Chiralpak AD-H
Mobile Phase:Hex/IPA=50/50(v/v)
Flow Rate: 1.0mL/min
CT: 30℃ **Sample Name:HYHI8-(R)+HYHG74-(S)**

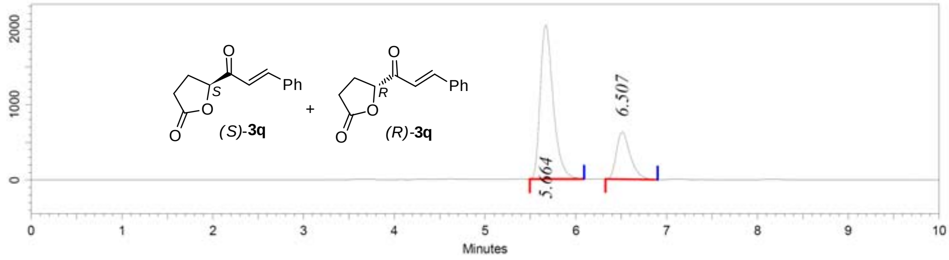

Detector A-264 nm

Pk #	Retention Time	Area	Area Percent
1	5.664	20223377	75.30
2	6.507	6634711	24.70
Totals		26858088	100.00

Chiral HPLC diagrams of enone *(S)*-3q

Shimadzu CLASS-VP V6.13 SP2 ***Area % Report***
Column: Chiralpak AD-H
Mobile Phase:Hex/IPA=50/50(v/v)
Flow Rate: 1.0mL/min
CT: 30℃ **Sample Name:HYHG74-(S)**

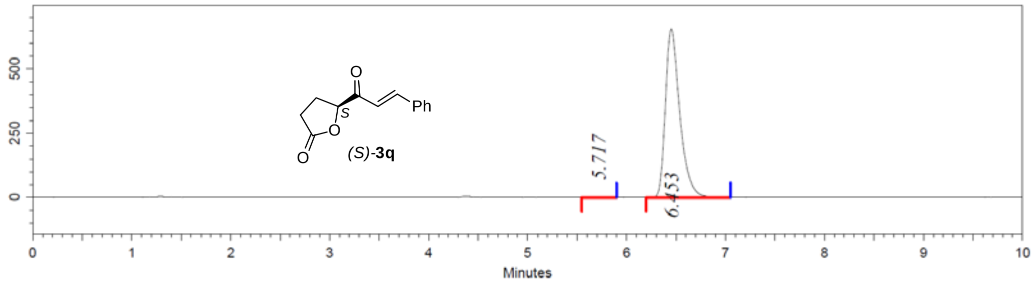

Detector A-264 nm

Pk #	Retention Time	Area	Area Percent
1	5.717	28653	0.43
2	6.453	6655022	99.57
Totals		6683675	100.00

Supplementary Figure 55. Chiral HPLC diagrams of enone *(S)*-3q and *(R)*-3q

Supplementary Methods

General Information. Melting points were determined on a Büchi M560 Automatic Melting Point apparatus and are uncorrected. ^{1}H NMR and ^{13}C NMR spectra were recorded on a Bruker spectrometer at 400 MHz (100 MHz for ^{13}C NMR), 500 MHz (125 MHz for ^{13}C NMR) or 600 MHz (150 MHz for ^{13}C NMR). Chemical shifts (δ) are reported in ppm and respectively referenced to internal standard Me_4Si and solvent signals (Me_4Si, 0 ppm for ^{1}H NMR and $CDCl_3$, 77.0 ppm for ^{13}C NMR). Multiplicities are described as follows: s (singlet), brs (broad singlet), d (doublet), t (triplet), q (quartet) and m (multiplet). Coupling constants (*J*) are reported in Hertz (Hz). ^{13}C NMR spectra were recorded with total proton decoupling. Infrared spectra were measured with a Nicolet Avatar 360 FT-IR spectrometer using film KBr pellet techniques. Mass spectra were recorded on a Bruker Dalton ESquire 3000 plus LC-MS apparatus (ESI direct injection). HRMS spectra were recorded on a 7.0T FT-MS apparatus.

Materials. Silica gel (300-400 mesh) was used for flash column chromatography (FC), eluting (unless otherwise stated) with ethyl acetate/ *n*-hexane mixture. Trifluoromethanesulfonic anhydride (Tf_2O) was distilled over phosphorous pentoxide and was stored for no more than a week before use. All other commercially available compounds were used as received. Dichloromethane was distilled over calcium hydride under Argon. All reactions were carried out under Argon.

General procedure for the direct C-H alkyliminylation of alkenes with secondary amides to give α,β-unsaturated ketimines 2 (General Procedure I).

Into a dry 10-mL round-bottom flask equipped with a magnetic stirring bar were added successively a secondary amide (0.5 mmol, 1.0 equiv), 2 mL of anhydrous CH_2Cl_2 and 2-fluoropyridine (0.6 mmol, 1.2 equiv) under an argon atmosphere. After being cooled to 0 °C, trifluoromethanesulfonic anhydride (Tf_2O) (155 mg, 93 μL, 0.55 mmol, 1.1 equiv) was added dropwise *via* a syringe and the reaction was stirred for 10 min. To the resulting mixture, alkenes (0.6 mmol, 1.2 equiv) was added dropwise at 0 °C. The mixture was allowed to warm-up to room temperature (or 40 °C) and stirred for 2 h. The reaction mixture was concentrated under reduced pressure, and the residue was purified by flash column chromatography (FC) on silica gel

(pre-neutralized with 2% Et_3N in *n*-hexane) to afford the desired α,β-unsaturated ketimine **2**.

General procedure for the direct C-H acylation of alkenes with secondary amides to give enones 3 (General Procedure II).

Into a dry 10-mL round-bottom flask equipped with a stirring bar were added successively a secondary amide (0.5 mmol, 1.0 equiv), 2 mL of anhydrous CH_2Cl_2 and 2xxx (0.6 mmol, 1.2 equiv) under an argon atmosphere. After being cooled to 0 °C, trifluoromethanesulfonic anhydride (Tf_2O) (155 mg, 93 μL, 0.55 mmol, 1.1 equiv) was added dropwise *via* a syringe and the reaction was stirred for 10 min. To the resulting mixture, alkenes (0.6 mmol, 1.2 equiv) was added dropwise at 0 °C. The mixture was allowed to warm-up to room temperature (or 40 °C) and stirred for 2 h. The reaction mixture was concentrated under reduced pressure. To the resulting residue were added 5 mL of EtOH and 5 mL of an aqueous solution of HCl (3.0 M). The resulting mixture was heated to reflux until completion of the reaction as monitored by TLC analysis (2-12 h). After being cooled to rt, 10 mL of CH_2Cl_2 was added, and the mixture extracted with CH_2Cl_2 (3 × 10 mL). The combined organic layers were washed with brine, dried over anhydrous Na_2SO_4, filtered, and concentrated under reduced pressure. The residue was purified by flash column chromatography on silica gel to afford the desired α,β-unsaturated ketone (enone) **3**.

Mechanistic studies

Investigation of the *in suit* formed intermediates by NMR

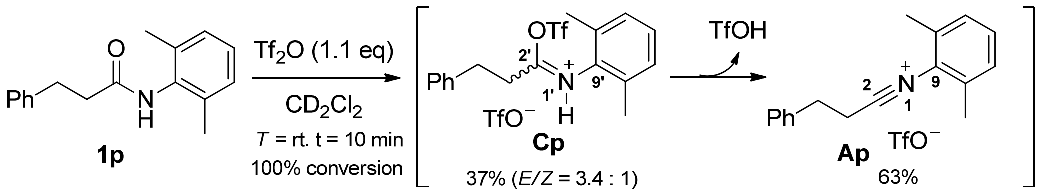

Procedure: To a dry NMR tube were sequentially added amide **1p** (25.3 mg, 0.1 mmol), CD_2Cl_2 (0.75 mL) at 0 °C, The NMR tube was slightly shaken. At this point, ^1H NMR was taken giving **spectrum A**.

Then the NMR tube was kept in ice-water bath. To the mixture Tf_2O (18.5 μL, 1.1 eq.) was added. The NMR tube was slightly shaken. The mixture was allowed to warm to rt and kept for 10 min. At this point, ^1H NMR was taken giving **spectrum B**.

Summary: The nitrinium ion intermediate **Ap** (63%) and the imidoyl trifluoromethyl sulfonate **Cp** (37%, the *E/Z* ratio is 3.4:1) were generated in the Tf_2O activated reaction. (The nitrilium ion **Ap** is confirmed by the observed resonances at δ_{C2} = 123.4 as a triplet with $J_{C13\text{-}N14}$ = 45.6 Hz, and δ_{C9} = 121.9 (t, $J_{C13\text{-}N14}$ = 13.5 Hz).)

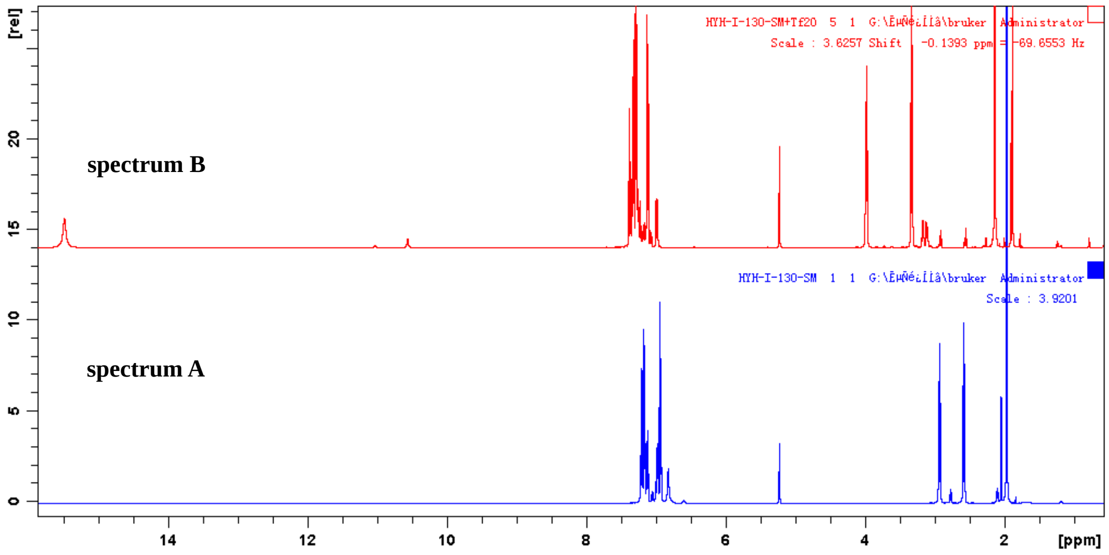

Supplementary Figure 56. The NMR analysis of the intermediate of Tf_2O-activated-**1p.**

Investigation of the *in suit* formed intermediates by NMR in the presence of 2-fluoropyridine

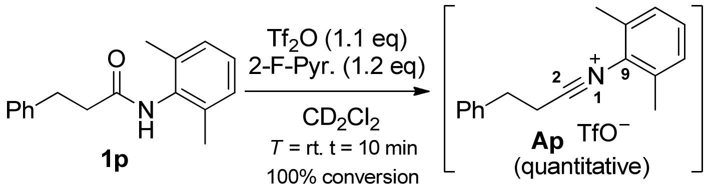

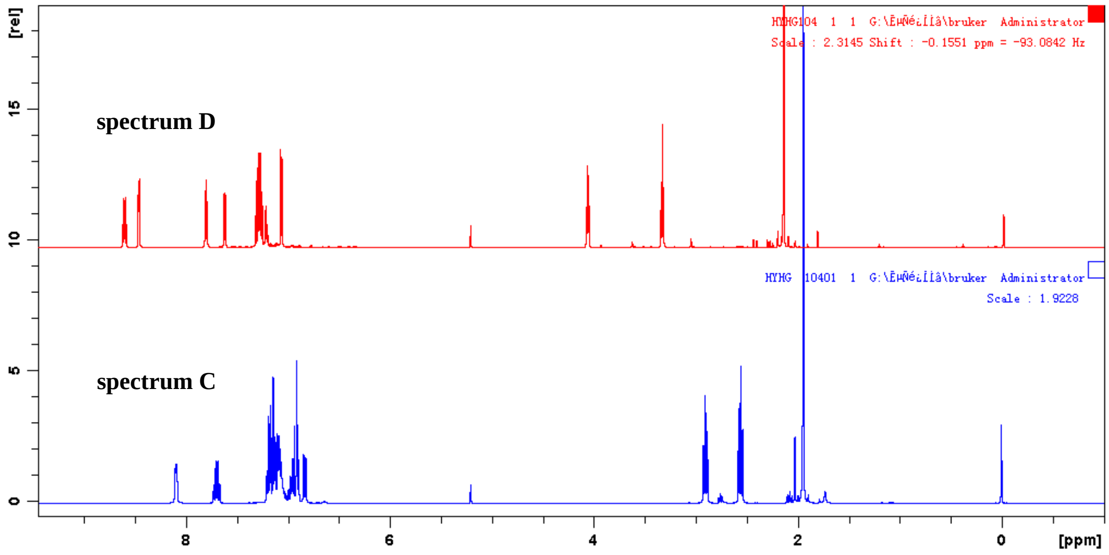

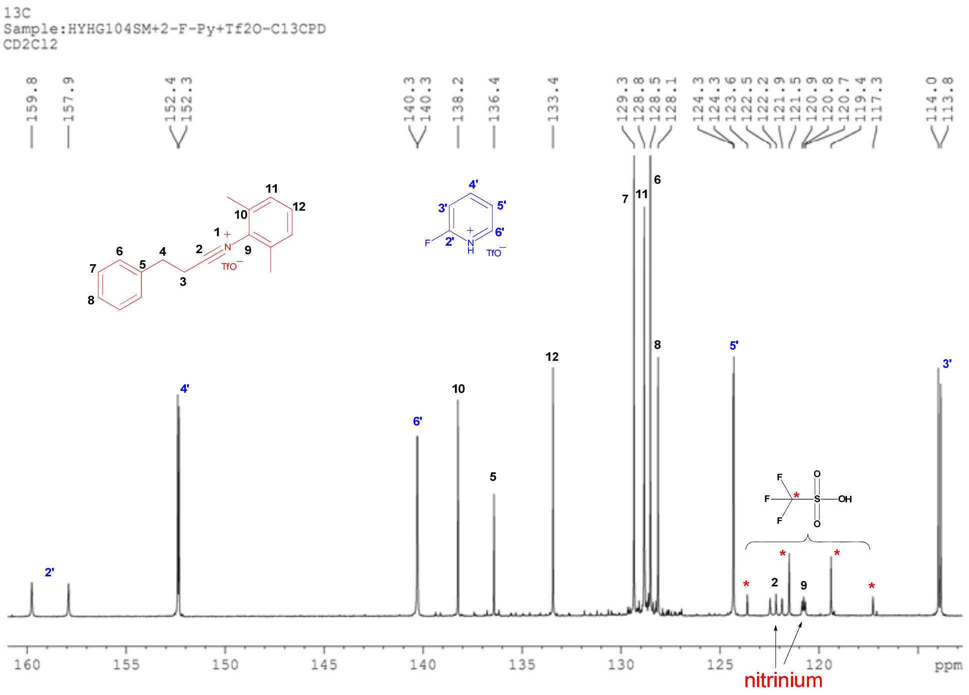

Supplementary Figure 57. The NMR analysis of the intermediate of Tf_2O-activated-**1p** in the presence of 2-fluoropyridine.

Spectrum C:

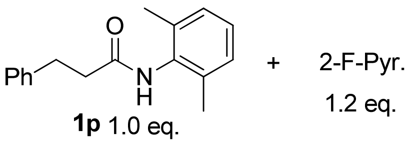

Spectrum D:

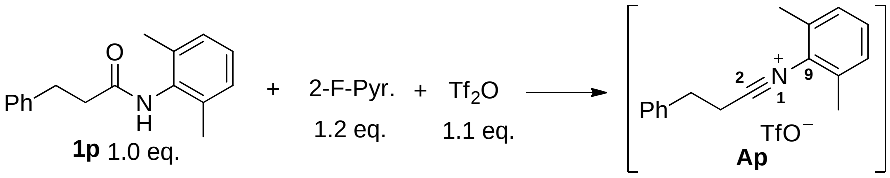

Investigation of the *in situ* generated intermediates by *in situ* IR

ReactIR® 15 (Mettler Toledo) was used for the *in situ* IR measurement.

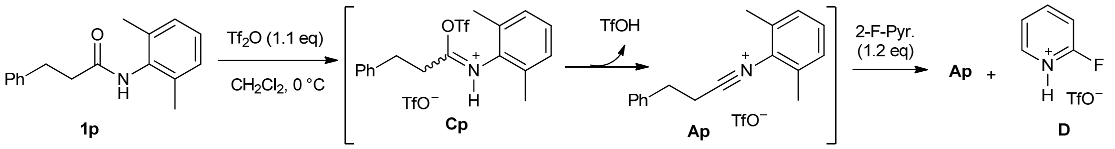

a

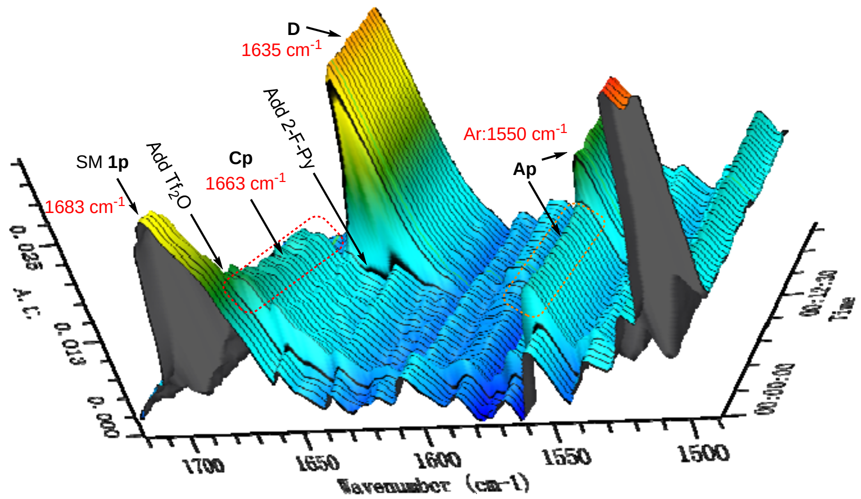

b

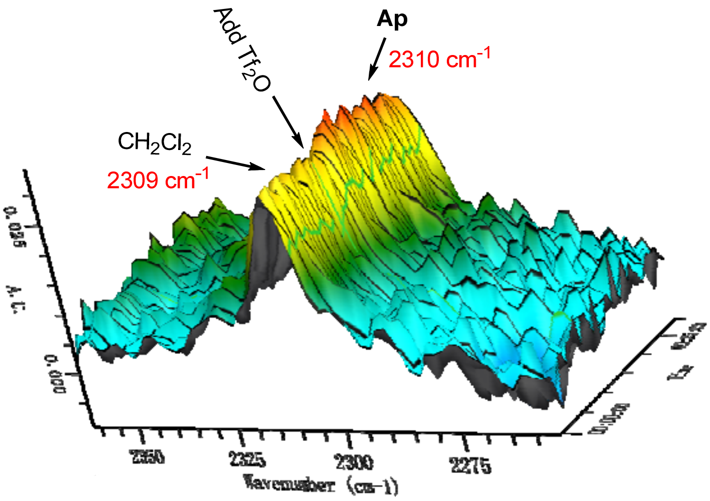

c

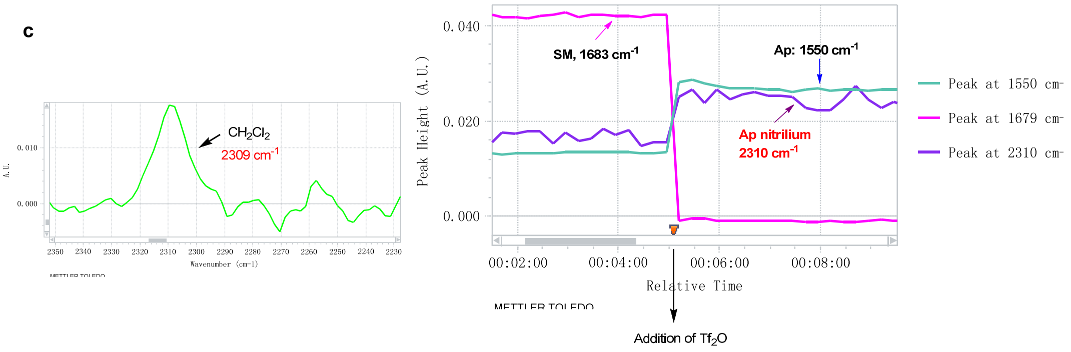

Supplementary Figure 58. The *in situ* IR analysis of the intermediates generated from the Tf_2O-activated **1p**.

Procedure and results: The starting material **1p** (126.5 mg, 0.5 mmol, 1.0 eq., 1683 cm^{-1}) was added to dry DCM (6.0 mL) under N_2. The mixture was cooled to 0 °C, the addition of Tf_2O (92.5 μL, 1.1 eq.) resulted in an immediate consumption of the starting material and appearance of an absorption consistent with an iminium salt **Cp** (1663 cm^{-1}) and an absorption consistent with a nitrilium trifluoromethanesulfonate salt **Ap** (2310 cm^{-1}). Addition of 2-F-Pyr. (52.0 μL, 1.2 eq.) resulted in the disappearance of the absorption of iminium salt **Cp** (1663 cm^{-1}), a drastic decrease in the absorption at 1663 cm^{-1} (**Cp**), and an increase in the intensity of the absorption at 2310 cm^{-1} (**Ap**).

Summary: The iminium salt **Cp**[3] and nitrilium trifluoromethanesulfonate salt **Ap**[1,2] are formed as soon as Tf_2O is added to the starting material **1p**; addition of the base 2-F-pyridine promoted a complete conversion of the iminium salt **Cp** to nitrilium salt **Ap**.

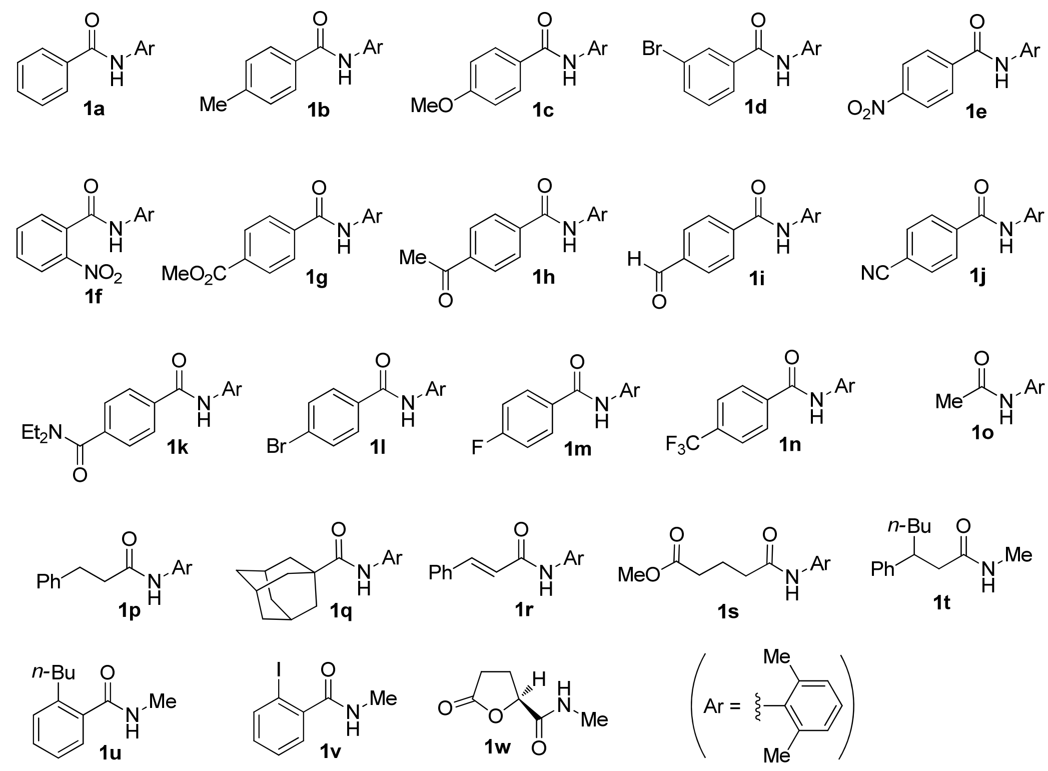

Supplementary Figure 59. Structures of Substrates

Compound Characterizations

N-[*(E)*-1,3-Diphenylallylidene]-2,6-dimethylaniline (2a)

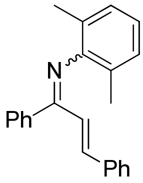

Following the general procedure **I**, the reaction of secondary amide **1a** (113 mg, 0.5 mmol) with styrene (69 μL, 0.6 mmol) gave, after FC (eluent: EtOAc/*n*-hexane = 1: 50), α,β-unsaturated ketimine **2a** (153 mg, yield: 98%). The geometry of the olefin moiety was determined as *E* according to the observed vicinal coupling constant (J_{vic} = 16.4 Hz). The ratio of geometric isomers of the ketimine moiety was determined (*E/Z* = 7.6: 1) by integration of the methyl peak in its ^{1}H NMR spectrum). The major geometric isomer was determined as *E* by NOESY technique (cf. SI-81). Yellow solid; Mp 61-62 °C; ^{1}H NMR (500 MHz, $CDCl_3$, data of the major geometric isomer read from the spectrum of the two geometric isomers) δ 7.74-7.81 (m, 2H), 7.46-7.52 (m, 3H), 7.20-7.28 (m, 5H), 7.03-7.09 (m, 2H), 6.87-6.97 (m, 2H), 6.63 (d, *J* = 16.4 Hz, 1H), 2.11 (s, 6H); ^{13}C NMR (125 MHz, $CDCl_3$, data of the major geometric isomer read from the spectrum of the two geometric isomers) δ 167.0, 148.4, 141.6, 138.9, 135.5, 129.7, 129.4, 128.9, 128.7, 128.3, 127.8, 127.5, 126.0, 123.0, 120.9, 18.1; IR (film) ν_{max}: 3058, 3020, 2917, 2857, 1620, 1588, 1444, 1198, 762, 691 cm^{-1}; HRMS calcd for $[C_{23}H_{22}N]^+$ (M+H$^+$): 312.1747; found: 312.1748.

2,6-Dimethyl-*N*-[(*E*)-1-phenyl-3-(*p*-tolyl)allylidene]aniline (2b)

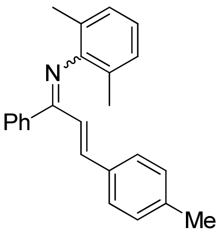

Following the general procedure **I**, the reaction of amide **1a** (113 mg, 0.5 mmol) with 4-methylphenylene (76 μL, 0.6 mmol) gave, after after FC (eluent: EtOAc/*n*-hexane = 1: 50), α,β-unsaturated ketimine **2b** (158 mg, yield: 97%, the ratio of geometric isomers *E/Z* = 7: 1). Yellow solid; Mp 91-92 °C; ^{1}H NMR (400 MHz, $CDCl_3$, data of the major geometric isomer read from the spectrum of the two geometric isomers) δ 7.73-7.79 (m, 2H), 7.47-7.53 (m, 3H), 7.12-7.17 (m, 2H), 7.03-7.10 (m, 4H), 6.91-6.97 (m, 1H), 6.89 (d, *J* = 16.4 Hz, 1H), 6.58 (d, *J* =

16.4 Hz, 1H), 2.30 (s, 3H), 2.10 (s, 6H); ^{13}C NMR (100 MHz, $CDCl_3$, data of the major geometric isomer read from the spectrum of the two geometric isomers) δ 167.2, 148.5, 141.7, 139.8, 139.0, 132.8, 129.6, 129.4, 128.9, 128.3, 127.8, 127.5, 126.1, 123.0, 120.0, 21.3, 18.1; IR (film) ν_{max}: 3021, 2971, 2917, 2851, 1619, 1590, 1570, 1512, 1317, 1192, 981, 810, 761, 699 cm^{-1}; HRMS calcd for $[C_{24}H_{24}N]^+$ $(M+H^+)$: 326.1903; found: 326.1901.

***N*-[(*E*)-3-(4-Methoxyphenyl)-1-phenylallylidene]-2,6-dimethylaniline (2c)**

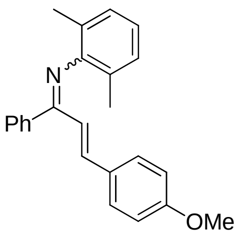

Following the general procedure **I**, the reaction of amide **1a** (113 mg, 0.5 mmol) with 4-methoxystyrene (81 μL, 0.6 mmol) gave, after FC (eluent: EtOAc/*n*-hexane = 1: 50), α,β-unsaturated ketimine **2c** (169 mg, yield: 99%, the ratio of geometric isomers *E/Z* = 7: 1). Yellow solid; Mp 77-78 °C; ^{1}H NMR (400 MHz, $CDCl_3$, data of the major geometric isomer read from the spectrum of the two geometric isomers) δ 7.72-7.80 (m, 2H), 7.44-7.50 (m, 3H), 7.13-7.19 (m, 2H), 7.02-7.08 (m, 2H), 6.81-6.96 (m, 2H), 6.71-6.79 (m, 2H), 6.51 (d, *J* = 16.3 Hz, 1H), 3.71 (s, 3H), 2.11 (s, 6H); ^{13}C NMR (100 MHz, $CDCl_3$, data of the major geometric isomer read from the spectrum of the two geometric isomers) δ 167.1, 160.7, 148.5, 141.3, 139.1, 129.4, 129.0, 128.8, 128.2, 128.1, 127.6, 126.0, 122.8, 118.6, 114.1, 55.1, 18.1; IR (film) ν_{max}: 3058, 3032, 2918, 2834, 1607, 1591, 1505, 1262, 1172, 823, 752, 704 cm^{-1}; HRMS calcd for $[C_{24}H_{24}NO]^+$ $(M+H^+)$: 342.1852; found: 342.1853.

***N*-[(*E*)-3-(4-Bromophenyl)-1-phenylallylidene]-2,6-dimethylaniline (2d)**

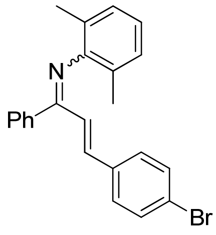

Following the general procedure **I**, the reaction of amide **1a** (113 mg, 0.5 mmol) with 4-bromostyrene (79 μL, 0.6 mmol) gave, after FC (eluent: EtOAc/*n*-hexane = 1: 50), α,β-unsaturated ketimine **2d** (191 mg, yield: 98%, the ratio of geometric isomers *E/Z* = 6: 1). Yellow solid; Mp 84-85 °C; ^{1}H NMR (400 MHz, $CDCl_3$, data of the major geometric isomer read

from the spectrum of the two geometric isomers) δ 7.72-7.79 (m, 2H), 7.47-7.54 (m, 3H), 7.37-7.42 (m, 2H), 7.04-7.13 (m, 4H), 6.92-6.98 (m, 1H), 6.84 (d, J = 16.4 Hz, 1H), 6.60 (d, J = 16.4 Hz, 1H), 2.09 (s, 6H); ^{13}C NMR (100 MHz, $CDCl_3$, data of the major geometric isomer read from the spectrum of the two geometric isomers) δ 166.7, 148.3, 140.2, 138.7, 134.4, 131.9, 129.8, 128.9, 128.9, 128.4, 127.9, 125.9, 123.6, 123.1, 121.4, 18.1; IR (film) ν_{max}: 3055, 3036, 2917, 2847, 1623, 1591, 1486, 1069, 768, 701 cm^{-1}; HRMS calcd for $[C_{23}H_{21}BrN]^+$ (M+H$^+$): 390.0852 and 392.0837; found: 390.0858 and 392.0838.

_N_-[(_E_)-3-(4-Chlorophenyl)-1-phenylallylidene]-2,6-dimethylaniline (2e)

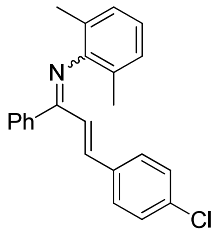

Following the general procedure **I**, the reaction of amide **1a** (113 mg, 0.5 mmol) with styrene (60 μL, 0.6 mmol) gave, after FC (eluent: EtOAc/n-hexane = 1: 50), α,β-unsaturated ketimine **2e** (167 mg, yield: 97%, the ratio of geometric isomers E/Z = 6: 1). Yellow solid; Mp 87-88 °C; ^{1}H NMR (500 MHz, $CDCl_3$, data of the major geometric isomer read from the spectrum of the two geometric isomers) δ 7.73-7.79 (m, 2H), 7.45-7.52 (m, 3H), 7.11-7.16 (m, 2H), 7.03-7.08 (m, 2H), 6.91-6.97 (m, 1H), 6.85 (d, J = 16.4 Hz, 1H), 6.59 (d, J = 16.4 Hz, 1H), 7.18-7.22 (m, 2H), 2.10 (s, 6H); ^{13}C NMR (125 MHz, $CDCl_3$, data of the major geometric isomer read from the spectrum of the two geometric isomers) δ 166.6, 148.3, 140.1, 138.7, 135.2, 134.0, 129.7, 128.9, 128.8, 128.6, 128.3, 127.9, 125.9, 123.1, 121.3, 18.1; IR (film) ν_{max}: 3058, 3036, 2917, 2850, 1620, 1585, 1492, 1200, 1089, 758, 698 cm^{-1}; HRMS calcd for $[C_{23}H_{21}ClN]^+$ (M+H$^+$): 346.1357; found: 346.1360.

_N_-[(_E_)-3-(4-Fluorophenyl)-1-phenylallylidene]-2,6-dimethylaniline (2f)

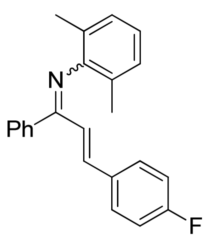

Following the general procedure **I**, the reaction of amide **1a** (113 mg, 0.5 mmol) with 4-fluorostyrene (72 μL, 0.6 mmol) gave, after FC (eluent: EtOAc/n-hexane = 1: 50), α,β-unsaturated ketimine **2f** (145 mg, yield: 88%, the ratio of geometric isomers E/Z = 6.8: 1).

Yellow oil; ^1H NMR (500 MHz, $CDCl_3$, data of the major geometric isomer read from the spectrum of the two geometric isomers) δ 7.72-7.79 (m, 2H), 7.45-7.54 (m, 3H), 7.19-7.25 (m, 2H), 7.04-7.09 (m, 2H), 6.92-7.00 (m, 3H), 6.87 (d, J = 16.4 Hz, 1H), 6.54 (d, J = 16.4 Hz, 1H), 2.10 (s, 6H); ^{13}C NMR (125 MHz, $CDCl_3$, data of the major geometric isomer read from the spectrum of the two geometric isomers) δ 166.9, 163.5 (d, J = 250.0 Hz), 148.4, 140.4, 138.8, 131.8 (d, J = 3.4 Hz), 129.7, 129.4, 129.3, 128.9, 128.4, 127.9, 126.0, 123.1, 120.7 (d, J = 2.4 Hz), 115.8 (d, J = 22.0 Hz), 18.1; IR (film) ν_{max}: 3061, 3029, 2914, 2847, 1597, 1508, 1236, 765 cm^{-1}; HRMS calcd for $[C_{23}H_{21}FN]^+$ $(M+H^+)$: 330.1653; found: 330.1654.

N-(1,3-Diphenylbut-2-en-1-ylidene)-2,6-dimethylaniline (2g)

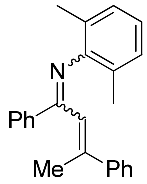

Following the general procedure **I**, the reaction of amide **1a** (113 mg, 0.5 mmol) with α-methylstyrene (78 μL, 0.6 mmol) gave, after FC (eluent: EtOAc/n-hexane = 1: 50), α,β-unsaturated ketimine **2g** (159 mg, yield: 98%, the geometric chemistry was not determined and the ratio of geometric isomers is 68: 14: 9: 9). Yellow solid;. Mp 92-94 °C; ^1H NMR (400 MHz, $CDCl_3$, data of the major geometric isomer read from the spectrum of the four geometric isomers) δ 7.94-7.99 (m, 2H), 7.43-7.50 (m, 3H), 7.23-7.28 (m, 3H), 7.15-7.19 (m, 2H), 6.99-7.04 (m, 2H), 6.86-6.92 (m, 1H), 6.14 (s, 1H), 2.08 (s, 6H), 1.82 (s, 3H); ^{13}C NMR (100 MHz, $CDCl_3$, data of the major geometric isomer read from the spectrum of the four geometric isomers) δ 166.7, 149.1, 143.0, 142.0, 139.3, 130.4, 128.5, 128.4, 128.2, 128.1, 127.8, 125.9, 125.7, 123.0, 122.8, 19.2, 18.0; IR (film) ν_{max}: 3059, 3021, 2918, 2847, 1619, 1590, 1570, 1445, 1196, 753, 690 cm^{-1}; HRMS calcd for $[C_{24}H_{24}N]^+$ $(M+H^+)$: 326.1903; found: 326.1903.

2,6-Dimethyl-N-(1,3,3-triphenylallylidene)aniline (2h)

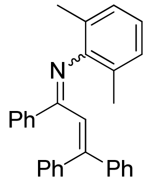

Following the general procedure **I**, the reaction of amide **1a** (113 mg, 0.5 mmol) with 1,1-diphenylethylene (85 μL, 0.6 mmol) gave, after FC (eluent: EtOAc/n-hexane = 1: 50),

α,β-unsaturated ketimine **2h** (194 mg, yield: 98%, the ratio of geometric isomers *E/Z* = 20: 1). Yellow solid; Mp 95-97 °C; ^{1}H NMR (500 MHz, $CDCl_3$) δ 7.71-7.77 (m, 2H), 7.18-7.28 (m, 7H), 7.09-7.15 (m, 2H), 6.98-7.04 (m, 2H), 6.93-6.97 (m, 2H), 6.81-6.87 (m, 3H), 6.43 (s, 1H), 2.00 (s, 6H); ^{13}C NMR (125 MHz, $CDCl_3$) δ 165.4, 149.7, 148.7, 142.1, 139.1, 139.1, 130.2, 129.5, 128.5, 128.4, 128.3, 128.2, 128.1, 127.8, 127.8, 127.6, 126.0, 122.9, 122.0, 18.1; IR (film) ν_{max}: 3059, 3017, 2922, 2851, 1603, 1590, 1574, 1445, 1200, 765, 699 cm^{-1}; HRMS calcd for $[C_{29}H_{26}N]^+$ ($M+H^+$): 388.2060; found: 388.2060.

***N*-(2-Cyclohexylidene-1-phenylethylidene)-2,6-dimethylaniline (2i)**

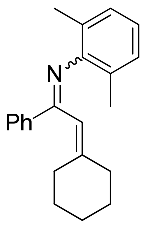

Following the general procedure **I**, the reaction of amide **1a** (113 mg, 0.5 mmol) with methylenecyclohexane (72 μL, 0.6 mmol) gave, after FC (eluent: EtOAc/*n*-hexane = 1: 50), α,β-unsaturated ketimine **2i** (150 mg, yield: 99%, the ratio of geometric isomers *E/Z* = 12: 1). Yellow oil; ^{1}H NMR (400 MHz, $CDCl_3$, data of the major geometric isomer read from the spectrum of the two geometric isomers) δ 7.94-8.02 (m, 2H), 7.38-7.47 (m, 3H), 7.02 (d, *J* = 7.4 Hz, 2H), 6.86-6.93 (m, 1H), 5.20-5.26 (m, 1H), 3.12-3.16 (m, 2H), 2.06 (s, 6H), 1.79-1.88 (m, 2H), 1.63-1.71 (m, 2H), 1.35-1.49 (m, 4H); ^{13}C NMR (100 MHz, $CDCl_3$, data of the major geometric isomer read from the spectrum of the two geometric isomers) δ 167.3, 148.4, 139.1, 132.8, 130.0, 128.1, 127.8, 127.7, 125.9, 125.2, 122.7, 39.2, 29.0, 25.2, 22.7, 21.9, 18.2; IR (film) ν_{max}: 3066, 3017, 2930, 2855, 1624, 1590, 1466, 1445, 1196, 761, 695 cm^{-1}; HRMS calcd for $[C_{22}H_{26}N]^+$ ($M+H^+$): 304.2060; found: 304.2060.

(*Z*)-2,6-Dimethyl-*N*-[(*E*)-3-methyl-1-phenylpenta-2,4-dien-1-ylidene]aniline (2j)

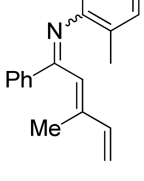

Following the general procedure **I**, the reaction of amide **1a** (113 mg, 0.5 mmol) with isoprene (60 μL, 0.6 mmol) gave, after FC (eluent: EtOAc/*n*-hexane = 1: 100), α,β-unsaturated ketimine **2j** (120

mg, yield: 87%). Yellow oil; ^{1}H NMR (400 MHz, $CDCl_3$) δ 7.82-7.92 (m, 2H), 7.40-7.50 (m, 3H), 6.98-7.03 (m, 2H), 6.85-6.91 (m, 1H), 6.27 (dd, J = 17.3, 10.7, 1H), 5.81 (brs, 1H), 5.25 (d, J = 17.3, 1H), 5.11 (d, J = 10.7, 1H), 2.03 (s, 6H), 1.52 (d, J = 0.9, 3H); ^{13}C NMR (100 MHz, $CDCl_3$) δ 166.5, 149.0, 141.5, 140.1, 139.5, 130.3, 128.4, 128.1, 127.8, 125.8, 125.6, 122.9, 115.7, 18.0, 15.0; IR (film) ν_{max}: 3062, 3023, 2920, 2847, 1611, 1588, 1470, 1444, 1201, 762, 701 cm^{-1}; HRMS calcd for $[C_{20}H_{22}N]^+$ $(M+H^+)$: 276.1747; found: 276.1747.

***N*-(2,3-Dimethyl-1-phenylbut-3-en-1-ylidene)-2,6-dimethylaniline (2k)**

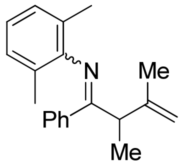

Following the general procedure **I**, the reaction of amide **1a** (113 mg, 0.5 mmol) with 2-methyl-2-butene (64 μL, 0.6 mmol) gave, after FC (eluent: EtOAc/*n*-hexane = 1: 20), α,β-unsaturated ketimine **2k** (118 mg, yield: 85%, the ratio of geometric isomers *E/Z* = 1:1). Yellow oil; ^{1}H NMR (400 MHz, $CDCl_3$, data of the mixture of the two geometric isomers) δ 7.15-7.25 (m, 3H), 7.06-7.13 (m, 2H), 6.89-6.95 (m, 1H), 6.74-6.86 (m, 2H), 4.84-4.89 (m, 2H), 3.81 (q, J = 6.3 Hz, 1H), 2.16 (s, 3H), 1.91 (s, 3H), 1.87 (s, 3H), 1.51 (d, J = 6.3 Hz, 3H); ^{13}C NMR (100 MHz, $CDCl_3$, data of the mixture of the two geometric isomers) δ 171.4, 169.7, 148.9, 148.4, 146.0, 139.2, 138.3, 132.4, 130.0, 128.5, 128.3, 127.9, 127.8, 127.6, 127.5, 126.6, 122.5, 122.4, 112.8, 50.6, 23.9, 20.5, 19.9, 18.6, 18.4, 18.3, 17.5, 16.2; IR (film) ν_{max}: 3067, 3025, 2988, 2926, 2847, 1615, 1586, 1573, 1449, 1200, 761, 694 cm^{-1}; HRMS calcd for $[C_{20}H_{24}N]^+$ $(M+H^+)$: 278.1903; found: 278.1903.

***N*-[Cyclohexa-1,5-dien-1-yl(phenyl)methylene]-2,6-dimethylaniline (2l)**

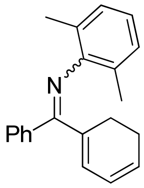

Following the general procedure **I**, the reaction of amide **1a** (113 mg, 0.5 mmol) with 1,3-cyclohexadiene (58 μL, 0.6 mmol) gave, after FC (eluent: EtOAc/*n*-hexane = 1: 100), α,β-unsaturated ketimine **2l** (123 mg, yield: 86%, the ratio of geometric isomers *E/Z* = 6: 1). Yellow oil; ^{1}H NMR (400 MHz, $CDCl_3$, data of the major geometric isomer read from the spectrum of the two geometric isomers) δ 7.13-7.22(m, 3H), 7.02-7.06 (m, 2H), 6.79-6.84 (m, 2H),

6.68-6.73 (m, 1H), 6.09-6.15 (m, 1H), 6.05-6.08 (m, 1H), 5.98-6.04 (m, 1H), 2.87 (t, *J* = 9.8 Hz, 2H), 2.37-2.45 (m, 2H), 2.00 (s, 6H); ^{13}C NMR (100 MHz, $CDCl_3$, data of the major geometric isomer read from the spectrum of the two geometric isomers) δ 168.2, 149.1, 137.9, 136.6, 132.3, 131.7, 128.2, 127.8, 127.4, 127.4, 125.4, 124.5, 122.3, 23.3, 22.0, 18.5; IR (film) ν_{max}: 3063, 3038, 2926, 2872, 2851, 1603, 1586, 1565, 1437, 1275, 1229, 1088, 761, 703, 674 cm^{-1}; HRMS calcd for $[C_{21}H_{22}N]^+$ (M+H$^+$): 288.1747; found: 288.1743.

2,6-Dimethyl-*N*-[(*E*)-3-phenyl-1-(*p*-tolyl)allylidene]aniline (2m)

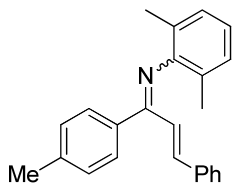

Following the general procedure **I**, the reaction of amide **1b** (120 mg, 0.5 mmol) with styrene (69 μL, 0.6 mmol) gave, after FC (eluent: EtOAc/*n*-hexane = 1: 50), α,β-unsaturated ketimine **2m** (159 mg, yield: 98%, the ratio of geometric isomers *E/Z* = 7: 1). Yellow oil; ^{1}H NMR (400 MHz, $CDCl_3$, data of the major geometric isomer read from the spectrum of the two geometric isomers) δ 7.66-7.78 (m, 2H), 7.22-7.33 (m, 7H), 7.01-7.08 (m, 2H), 6.90-6.96 (m, 2H), 6.61 (d, *J* = 16.3 Hz, 1H), 2.45 (s, 3H), 2.09 (s, 6H); ^{13}C NMR (100 MHz, $CDCl_3$, data of the major geometric isomer read from the spectrum of the two geometric isomers) δ 166.8, 148.6, 141.3, 139.9, 136.1, 135.7, 129.4, 129.0, 129.0, 128.7, 127.8, 127.5, 126.1, 122.9, 121.2, 21.4, 18.1; IR (film) ν_{max}: 3063, 3029, 2918, 2851, 1624, 1586, 1470, 1449, 1312, 1196, 818, 760, 694 cm^{-1}; HRMS calcd for $[C_{24}H_{24}N]^+$ (M+H$^+$): 326.1903; found: 326.1903.

***N*-[(*E*)-1-(4-Methoxyphenyl)-3-phenylallylidene]-2,6-dimethylaniline (2n)**

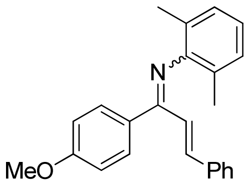

Following the general procedure **I**, the reaction of amide **1c** (128 mg, 0.5 mmol) with styrene (69 μL, 0.6 mmol) gave, after FC (eluent: EtOAc/*n*-hexane = 1: 50), α,β-unsaturated ketimine **2n** (167 mg, yield: 98%, the ratio of geometric isomers *E/Z* = 7: 1). Yellow oil; ^{1}H NMR (400 MHz, $CDCl_3$, data of the major geometric isomer read from the spectrum of the two geometric isomers) δ 7.73-7.80 (m, 2H), 7.21-7.31 (m, 5H), 6.98-7.09 (m, 4H), 6.84-6.96 (m, 2H), 6.61 (d, *J* = 16.4

Hz, 1H), 3.87 (s, 3H), 2.09 (s, 6H); ^{13}C NMR (100 MHz, $CDCl_3$, data of the major geometric isomer read from the spectrum of the two geometric isomers) δ 166.1, 161.0, 148.7, 141.0, 135.7, 131.4, 130.5, 129.3, 128.7, 127.8, 127.5, 126.1, 122.9, 121.4, 113.7, 55.4, 18.1; IR (film) ν_{max}: 3058, 3034, 2922, 2835, 1607, 1582, 1503, 1312, 1254, 1163, 1030, 757, 694 cm^{-1}; HRMS calcd for $[C_{24}H_{24}NO]^+$ ($M+H^+$): 342.1852; found: 342.1852.

***N*-[(*E*)-1-(3-Bromophenyl)-3-phenylallylidene]-2,6-dimethylaniline (2o)**

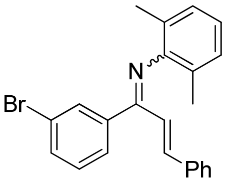

Following the general procedure **I**, the reaction of amide **1d** (152 mg, 0.5 mmol) with styrene (69 μL, 0.6 mmol) gave, after FC (eluent: EtOAc/*n*-hexane = 1: 50), α,β-unsaturated ketimine **2o** (171 mg, yield: 88%, the ratio of geometric isomers *E/Z* = 8: 1). Yellow oil; ^{1}H NMR (400 MHz, $CDCl_3$, data of the major geometric isomer read from the spectrum of the two geometric isomers) δ 7.90-7.94 (m, 1H), 7.60-7.71 (m, 2H), 7.33-7.39 (m, 1H), 7.21-7.29 (m, 5H), 7.04-7.09 (m, 2H), 6.92-6.98 (m, 1H), 6.89 (d, *J* = 16.4 Hz, 1H), 6.60 (d, *J* = 16.4 Hz, 1H), 2.09 (s, 6H); ^{13}C NMR (100 MHz, $CDCl_3$, data of the major geometric isomer read from the spectrum of the two geometric isomers) δ 165.6, 148.1, 141.8, 140.9, 135.3, 132.7, 131.8, 129.8, 129.7, 128.7, 127.9, 127.6, 127.5, 125.9, 123.3, 122.6, 120.5, 18.1; IR (film) ν_{max}: 3055, 3033, 2917, 2847, 1627, 1585, 1556, 1476, 1313, 1191, 752, 698 cm^{-1}; HRMS calcd for $[C_{23}H_{21}BrN]^+$ ($M+H^+$): 390.0852 and 392.0837; found: 390.0857 and 392.0836.

2,6-Dimethyl-*N*-[(*E*)-1-(4-nitrophenyl)-3-phenylallylidene]aniline (2p)

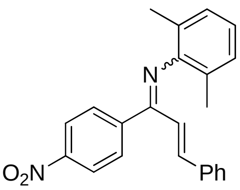

Following the general procedure **I**, the reaction of amide **1e** (135 mg, 0.5 mmol) with styrene (69 μL, 0.6 mmol) gave, after FC (eluent: EtOAc/*n*-hexane = 1: 5), α,β-unsaturated ketimine **2p** (137 mg, yield: 77%, the ratio of geometric isomers *E/Z* = 8: 1). Yellow solid; Mp 169-171 °C; ^{1}H NMR (400 MHz, $CDCl_3$, data of the major geometric isomer read from the spectrum of the two geometric isomers) δ 8.33-8.39 (m, 2H), 7.90-7.97 (m, 2H), 7.23-7.33 (m, 5H), 7.06-7.12 (m, 2H),

6.95-7.02 (m, 1H), 6.85 (d, J = 16.5 Hz, 1H), 6.65 (d, J = 16.5 Hz, 1H), 2.10 (s, 6H); ^{13}C NMR (100 MHz, $CDCl_3$, data of the major geometric isomer read from the spectrum of the two geometric isomers) δ 18.1, 120.0, 123.6, 123.6, 125.7, 127.7, 128.0, 128.8, 129.9, 130.0, 135.0, 142.2, 144.9, 147.8, 148.6, 165.4; IR (film) ν_{max}: 3075, 3034, 2918, 2843, 1582, 1520, 1350, 1317, 981, 753, 703 cm^{-1}; HRMS calcd for $[C_{23}H_{21}N_2O_2]^+$ (M+H$^+$): 357.1598; found: 357.1598.

(*E*)-2,6-Dimethyl-*N*-[(*E*)-1-(2-nitrophenyl)-3-phenylallylidene]aniline (2q)

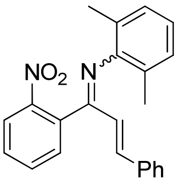

Following the general procedure **I**, the reaction of amide **1f** (135 mg, 0.5 mmol) with styrene (69 μL, 0.6 mmol) gave, after FC (eluent: EtOAc/*n*-hexane = 1: 5), α,β-unsaturated ketimine **2q** (167 mg, yield: 94%, the ratio of geometric isomers *E/Z* > 20: 1). Yellow solid; Mp 134-136 °C; ^{1}H NMR (400 MHz, $CDCl_3$) δ 8.13-8.17 (m, 1H), 7.76-7.81 (m, 1H), 7.70-7.75 (m, 1H), 7.61-7.67 (m, 1H), 7.15-7.26 (m, 5H), 7.06-7.11(m, 2H), 6.95-7.01 (m, 1H), 6.66 (d, J = 16.4 Hz, 1H), 6.51 (d, J = 16.4 Hz, 1H), 2.20 (s, 6H); ^{13}C NMR (100 MHz, $CDCl_3$) δ 165.0, 148.1, 147.1, 140.0, 134.9, 134.7, 133.8, 131.4, 129.9, 129.7, 128.7, 128.0, 127.7, 126.6, 124.5, 123.6, 121.0, 18.2; IR (film) ν_{max}: 3063, 3038, 2922, 1624, 1590, 1532, 1317, 757, 699 cm^{-1}; HRMS calcd for $[C_{23}H_{20}N_2NaO_2]^+$ (M+Na$^+$): 379.1417; found: 379.1413.

Methyl 4-{(2*E*)-1-[(2,6-dimethylphenyl)imino]-3-phenylallyl}benzoate (2r)

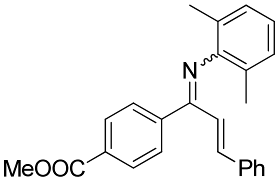

Following the general procedure **I**, the reaction of amide **1g** (142 mg, 0.5 mmol) with styrene (69 μL, 0.6 mmol) gave, after FC (eluent: EtOAc/*n*-hexane = 1: 10), α,β-unsaturated ketimine **2r** (172 mg, yield: 93%, the ratio of geometric isomers *E/Z* = 6: 1). Yellow solid; Mp 119-121 °C; ^{1}H NMR (400 MHz, $CDCl_3$, data of the major geometric isomer read from the spectrum of the two geometric isomers) δ 8.14-8.21 (m, 2H), 7.80-7.86 (m, 2H), 7.22-7.31 (m, 5H), 7.04-7.11 (m, 2H), 6.93-6.99 (m, 1H), 6.86 (d, J = 16.4 Hz, 1H), 6.63 (d, J = 16.4 Hz, 1H), 3.97 (s, 3H), 2.10 (s, 6H); ^{13}C NMR (100 MHz, $CDCl_3$, data of the major geometric isomer read from the spectrum of the

two geometric isomers) δ 166.7, 166.4, 148.1, 143.2, 142.0, 135.3, 131.2, 129.7, 129.6, 128.9, 128.8, 127.9, 127.6, 125.9, 123.3, 120.5, 52.2, 18.1; IR (film) v_{max}: 3059, 3034, 2951, 2918, 1727, 1619, 1586, 1275, 1192, 1113, 752 cm^{-1}; HRMS calcd for $[C_{25}H_{24}NO_2]^+$ ($M+H^+$): 370.1802; found: 370.1802.

1-{4-{(2*E*)-1-[(2,6-Dimethylphenyl)imino]-3-phenylallyl}phenyl}ethanone (2s)

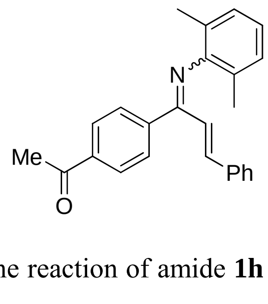

Following the general procedure **I**, the reaction of amide **1h** (134 mg, 0.5 mmol) with styrene (69 μL, 0.6 mmol) gave, after FC (eluent: EtOAc/*n*-hexane = 1: 50), α,β-unsaturated ketimine **2s** (154 mg, yield: 87%, the ratio of geometric isomers *E/Z* = 7: 1). Yellow solid; Mp 130-131 °C; ^{1}H NMR (400 MHz, $CDCl_3$, data of the major geometric isomer read from the spectrum of the two geometric isomers) δ 8.06-8.12 (m, 2H), 7.83-7.89 (m, 2H), 7.22-7.31 (m, 5H), 7.05-7.11 (m, 2H), 6.94-7.00 (m, 1H), 6.87 (d, *J* = 16.4 Hz, 1H), 6.63 (d, *J* = 16.4 Hz, 1H), 2.10 (s, 6H), 2.68 (s, 3H); ^{13}C NMR (100 MHz, $CDCl_3$, data of the major geometric isomer read from the spectrum of the two geometric isomers) δ 197.7, 166.3, 148.1, 143.3, 141.9, 137.9, 135.3, 129.8, 129.2, 128.8, 128.4, 128.0, 127.6, 125.9, 123.3, 120.5, 26.8, 18.1; IR (film) v_{max}: 3059, 3038, 2913, 2851, 1686, 1624, 1582, 1263, 753 cm^{-1}; HRMS calcd for $[C_{25}H_{24}NO]^+$ ($M+H^+$): 354.1852; found: 354.1852.

4-{(2*E*)-1-[(2,6-Dimethylphenyl)imino]-3-phenylallyl}benzaldehyde (2t)

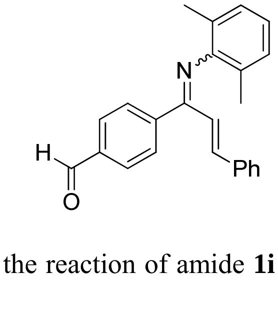

Following the general procedure **I**, the reaction of amide **1i** (127 mg, 0.5 mmol) with styrene (69 μL, 0.6 mmol) gave, after FC (eluent: EtOAc/*n*-hexane = 1: 50), α,β-unsaturated ketimine **2t** (139 mg, yield: 82%, the ratio of geometric isomers *E/Z* = 8: 1). Yellow oil; ^{1}H NMR (400 MHz, $CDCl_3$, data of the major geometric isomer read from the spectrum of the two geometric isomers) δ 10.13 (s, 1H), 8.00-8.06 (m, 2H), 7.90-7.96 (m, 2H), 7.22-7.33 (m, 5H), 7.06-7.12 (m, 2H), 6.95-7.00 (m, 1H), 6.86 (d, *J* = 16.5 Hz, 1H), 6.64 (d, *J* = 16.5 Hz, 1H), 2.11 (s, 6H); ^{13}C NMR

(100 MHz, $CDCl_3$, data of the major geometric isomer read from the spectrum of the two geometric isomers) δ 191.8, 166.2, 148.0, 144.6, 142.1, 137.0, 135.1, 129.8, 129.7, 129.5, 128.8, 128.0, 127.6, 125.8, 123.4, 120.3, 18.1; IR (film) ν_{max}: 3062, 3033, 2917, 2850, 2735, 1703, 1585, 1198, 759 cm^{-1}; HRMS calcd for $[C_{24}H_{21}NNaO]^+$ ($M+Na^+$): 362.1515; found: 362.1517.

4-{(2*E*)-1-[(2,6-Dimethylphenyl)imino]-3-phenylallyl}benzonitrile (2u)

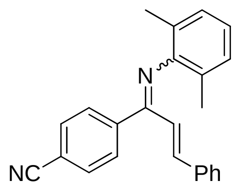

Following the general procedure **I**, the reaction of amide **1j** (125 mg, 0.5 mmol) with styrene (69 μL, 0.6 mmol) gave, after FC (eluent: EtOAc/*n*-hexane = 1: 20), α,β-unsaturated ketimine **2u** (152 mg, yield: 90%, the ratio of geometric isomers *E/Z* = 9: 1). Yellow solid; Mp 137-139 °C; ^1H NMR (400 MHz, $CDCl_3$, data of the major geometric isomer read from the spectrum of the two geometric isomers) δ 7.85-7.91 (m, 2H), 7.77-7.83 (m, 2H), 7.22-7.32 (m, 5H), 7.05-7.11 (m, 2H), 6.95-7.01 (m, 1H), 6.83 (d, J = 16.5 Hz, 1H), 6.62 (d, J = 16.5 Hz, 1H), 2.09 (s, 6H); ^{13}C NMR (100 MHz, $CDCl_3$, data of the major geometric isomer read from the spectrum of the two geometric isomers) δ 165.6, 147.9, 143.2, 142.1, 135.0, 132.2, 130.0, 129.6, 128.8, 128.0, 127.6, 125.7, 123.6, 120.1, 118.5, 113.4, 18.1; IR (film) ν_{max}: 3063, 3038, 2926, 2855, 2233, 1624, 1582, 1445, 1312, 1188, 764, 690 cm^{-1}; HRMS calcd for $[C_{24}H_{21}N_2]^+$ ($M+H^+$): 337.1699; found: 337.1699.

4-{(2*E*)-1-[(2,6-Dimethylphenyl)imino]-3-phenylallyl}-*N*,*N*-diethylbenzamide (2v)

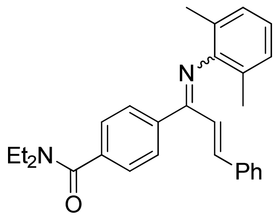

Following the general procedure **I**, the reaction of amide **1k** (164 mg, 0.5 mmol) with styrene (69 μL, 0.6 mmol) gave, after FC (eluent: EtOAc/*n*-hexane = 1: 5), α,β-unsaturated ketimine **2v** (179 mg, yield: 87%, the ratio of geometric isomers *E/Z* = 10: 1). Yellow oil; ^1H NMR (400 MHz, $CDCl_3$, data of the major geometric isomer read from the spectrum of the two geometric isomers) δ 7.75-7.82 (m, 2H), 7.48-7.55 (m, 2H), 7.21-7.33 (m, 5H), 7.05-7.11 (m, 2H), 6.88-7.00 (m, 2H),

6.63 (d, J = 16.4 Hz, 1H), 3.50-3.67 (m, 2H), 3.24-3.40 (m, 2H), 2.12 (s, 6H), 1.10-1.34 (m, 6H); ^{13}C NMR (100 MHz, $CDCl_3$, data of the major geometric isomer read from the spectrum of the two geometric isomers) δ 170.8, 166.8, 148.1, 142.2, 139.7, 138.4, 135.3, 129.6, 129.0, 128.7, 127.9, 127.6, 126.3, 125.9, 123.2, 120.4, 43.2, 39.2, 18.1, 14.2, 12.9; IR (film) ν_{max}: 3059, 3025, 2971, 2930, 2876, 2851, 1624, 1590, 1474, 1429, 1325, 1288, 1097, 761, 694 cm^{-1}; HRMS calcd for $[C_{28}H_{31}N_2O]^+$ ($M+H^+$): 411.2431; found: 411.2425.

4-{(*1E,3E*)-3-[(2,6-Dimethylphenyl)imino]-3-(2-nitrophenyl)prop-1-en-1-yl}phenyl acetate (2w)

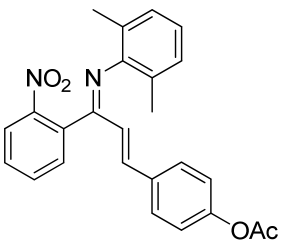

Following the general procedure **I**, the reaction of amide **1f** (135 mg, 0.5 mmol) with 4-vinylphenyl acetate (98 mg, 0.6 mmol) gave, after FC (eluent: EtOAc/*n*-hexane = 1: 5), α,β-unsaturated ketimine **2w** (182 mg, yield: 88%). The structure determined by X-Ray analysis (cf. SI-82).Yellow solid; Mp 136-138 °C; ^{1}H NMR (500 MHz, $CDCl_3$) δ 8.13-8.19 (m, 1H), 7.77-7.84 (m, 1H), 7.71-7.75 (m, 1H), 7.62-7.69 (m, 1H), 7.16-7.23 (m, 2H), 7.06-7.12 (m, 2H), 6.95-7.02 (m, 3H), 6.60 (d, J = 16.4 Hz, 1H), 6.48 (d, J = 16.4 Hz, 1H), 2.26 (s, 3H), 2.19 (s, 6H); ^{13}C NMR (125 MHz, $CDCl_3$) δ 169.0, 164.8, 151.6, 148.1, 147.1, 138.7, 134.6, 133.8, 132.7, 131.4, 130.0, 128.8, 128.1, 126.6, 124.5, 123.6, 121.9, 121.2, 21.0, 18.2; IR (film) ν_{max}: 3068, 3039, 2917, 2847, 1761, 1623, 1595, 1524, 1348, 1194, 1162 cm^{-1}; HRMS calcd for $[C_{25}H_{22}N_2NaO_4]^+$ ($M+Na^+$): 437.1472; found: 437.1468.

(*E*)-*N*-{(*E*)-3-[4-(Azidomethyl)phenyl]-1-(2-nitrophenyl)allylidene}-2,6-dimethylaniline (2x)

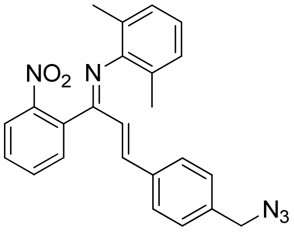

Following the general procedure **I**, the reaction of amide **1f** (135 mg, 0.5 mmol) with 1-(azidomethyl)-4-vinylbenzene (96 mg, 0.6 mmol) gave, after FC (eluent: EtOAc/*n*-hexane = 1: 5), α,β-unsaturated ketimine **2x** (189 mg, yield: 92%). Yellow oil; ^{1}H NMR (500 MHz, $CDCl_3$) δ

8.13-8.21 (m, 1H), 7.78-7.84 (m, 1H), 7.72-7.76 (m, 1H), 7.64-7.71 (m, 1H), 7.20 (br s, 4H), 7.07-7.12 (m, 2H), 6.96-7.03 (m, 1H), 6.66 (d, *J* = 16.4 Hz, 1H), 6.50 (d, *J* = 16.4 Hz, 1H), 4.28 (s, 2H), 2.20 (s, 6H); ^{13}C NMR (125 MHz, $CDCl_3$) δ 164.8, 148.1, 147.1, 139.1, 137.0, 135.0, 134.7, 133.8, 131.4, 130.0, 128.4, 128.2, 128.1, 126.6, 124.5, 123.7, 121.5, 54.3, 18.2; IR (film) ν_{max}: 3062, 3030, 2924, 2853, 2098, 1623, 1591, 1527, 1348 cm^{-1}; HRMS calcd for $[C_{24}H_{21}N_5NaO_2]^+$ (M+Na$^+$): 434.1587; found: 434.1586.

***N*-Benzyl-*N*-{4-{(*1E,3E*)-3-[(2,6-dimethylphenyl)imino]-3-(2-nitrophenyl)prop-1-en-1-yl}phenyl}acetamide (2y)**

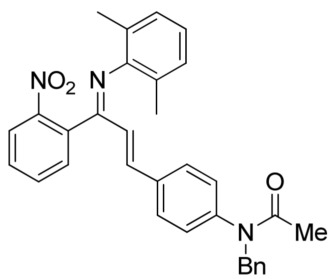

Following the general procedure **I**, the reaction of amide **1f** (135 mg, 0.5 mmol) with *N*-benzyl-*N*-(4-vinylphenyl)acetamide (151 mg, 0.6 mmol) gave, after FC (eluent: EtOAc/*n*-hexane = 1: 5), α,β-unsaturated ketimine **2y** (216 mg, yield: 86%). Yellow oil; ^{1}H NMR (500 MHz, $CDCl_3$) δ 8.14-8.20 (m, 1H), 7.77-7.84 (m, 1H), 7.63-7.75 (m, 2H), 7.19-7.26 (m, 3H), 7.06-7.16 (m, 6H), 6.95-7.02 (m, 1H), 6.80-6.92 (m, 2H), 6.62 (d, *J* = 16.4 Hz, 1H), 6.45 (d, *J* = 16.4 Hz, 1H), 4.82 (s, 2H), 2.19 (s, 6H), 1.84 (s, 3H); ^{13}C NMR (125 MHz, $CDCl_3$) δ 170.0, 164.7, 148.0, 147.0, 143.8, 138.3, 137.1, 134.5, 133.9, 131.4, 130.1, 128.8, 128.6, 128.4, 128.4, 128.1, 127.4, 126.5, 124.5, 123.7, 121.8, 52.5, 22.7, 18.2; IR (film) ν_{max}: 3062, 3035, 2924, 2847, 1661, 1598, 1527, 1389, 1345, 1322, 710 cm^{-1}; HRMS calcd for $[C_{32}H_{29}N_3NaO_3]^+$ (M+Na$^+$): 526.2101; found: 526.2103.

(*8R,9S,13S,14S*)-3-{(*1E,3E*)-3-[(2,6-Dimethylphenyl)imino]-3-(2-nitrophenyl)prop-1-en-1-yl}-13-methyl-7,8,9,11,12,13,15,16-octahydro-*6H*-cyclopenta[a]phenanthren-17(*14H*)-one (2z)

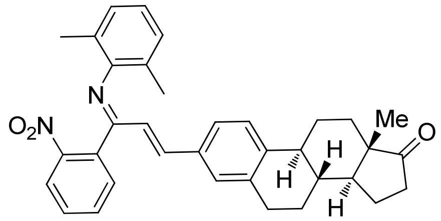

Following the general procedure **I**, the reaction of amide **1f** (135 mg, 0.5 mmol) with

3-vinylestrone[4] (168 mg, 0.6 mmol) gave, after FC (eluent: EtOAc/*n*-hexane = 1: 5), α,β-unsaturated ketimine **2z** (247 mg, yield: 93%). Yellow oil; ^{1}H NMR (500 MHz, $CDCl_3$) δ 8.13-8.18 (m, 1H), 7.77-7.83 (m, 1H), 7.71-7.75 (m, 1H), 7.63-7.69 (m, 1H), 7.15-7.19 (m, 1H), 7.07-7.11 (m, 2H), 6.96-7.01 (m, 2H), 6.92 (s, 1H), 6.60 (d, J = 16.4 Hz, 1H), 6.46 (d, J = 16.4 Hz, 1H), 2.77-2.85 (m, 2H), 2.49 (dd, J = 19.1, 8.8 Hz, 1H), 2.30-2.39 (m, 1H), 2.20-2.28 (m, 1H), 2.20 (s, 3H), 2.19 (s, 3H), 2.08-2.17 (m, 1H), 1.89-2.07 (m, 3H), 1.34-1.65 (m, 6H), 0.88 (s, 3H); ^{13}C NMR (125 MHz, $CDCl_3$) δ 220.6, 165.0, 148.1, 147.2, 142.0, 140.0, 137.0, 134.8, 133.8, 132.5, 131.4, 129.9, 128.5, 128.0, 126.7, 126.7, 125.7, 125.0, 124.4, 123.5, 120.4, 50.4, 47.8, 44.4, 37.9, 35.8, 31.5, 29.1, 26.2, 25.5, 21.5, 18.2, 13.7; IR (film) ν_{max}: 3062, 3049, 2921, 2850, 1739, 1620, 1527, 1344 cm^{-1}; HRMS calcd for $[C_{35}H_{37}N_2O_3]^+$ (M+H$^+$): 533.2799; found: 533.2795.

***N*-{4-{(*1E,3E*)-3-[(2,6-Dimethylphenyl)imino]-3-(2-nitrophenyl)prop-1-en-1-yl}benzyl}-4-methylbenzenesulfonamide (2aa)**

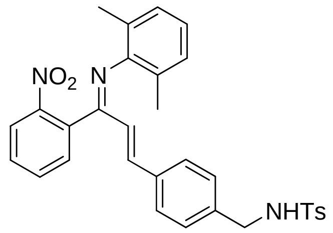

Following the general procedure **I**, the reaction of amide **1f** (135 mg, 0.5 mmol) with 4-methyl-*N*-(4-vinylbenzyl)benzenesulfonamide (172 mg, 0.6 mmol) gave, after FC (eluent: EtOAc/*n*-hexane = 1: 5), α,β-unsaturated ketimine **2aa** (261 mg, yield: 97%). Yellow solid; Mp 207-209 °C; ^{1}H NMR (500 MHz, $CDCl_3$) δ 8.12-8.17 (m, 1H), 7.78-7.83 (m, 1H), 7.71-7.75 (m, 1H), 7.64-7.69 (m, 3H), 7.22-7.27 (m, 2H), 7.04-7.11 (m, 6H), 6.96-7.01 (m, 1H), 6.59 (d, J = 16.4 Hz, 1H), 6.44 (d, J = 16.4 Hz, 1H), 5.07 (t, J = 6.3 Hz, 1H), 4.02 (d, J = 6.3 Hz, 2H), 2.39 (s, 3H), 2.18 (s, 6H); ^{13}C NMR (125 MHz, $CDCl_3$) δ 164.9, 148.0, 147.0, 143.5, 139.2, 138.3, 136.8, 134.5, 134.4, 133.9, 131.4, 130.0, 129.7, 128.1, 127.9, 127.0, 126.6, 124.5, 123.7, 121.2, 46.7, 21.4, 18.2; IR (film) ν_{max}: 3283, 3067, 3033, 2923, 1620, 1595, 1521, 1357, 1319, 1162 cm^{-1}; HRMS calcd for $[C_{31}H_{30}N_3O_4S]^+$ (M+H$^+$): 540.1952; found: 540.1951.

4-{(*1E,3E*)-3-[(2,6-Dimethylphenyl)imino]-3-(2-nitrophenyl)prop-1-en-1-yl}phenol (2ab)

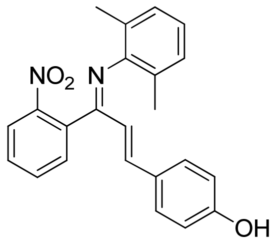

Following the general procedure **I**, the reaction of amide **1f** (135 mg, 0.5 mmol) with 4-vinylphenol (72 mg, 0.6 mmol) gave, after FC (eluent: EtOAc/*n*-hexane = 1: 2), α,β-unsaturated ketimine **2ab** (151 mg, yield: 81%). Yellow solid; Mp 193-195 °C; ^{1}H NMR (500 MHz, CD_3OD) δ 8.21-8.26 (m, 1H), 7.90-7.96 (m, 1H), 7.76-7.84 (m, 2H), 7.12-7.17 (m, 2H), 7.01-7.10 (m, 3H), 6.67-6.73 (m, 2H), 6.60 (d, J = 16.2 Hz, 1H), 6.50 (d, J = 16.2 Hz, 1H), 2.22 (s, 6H); ^{13}C NMR (125 MHz, CD_3OD) δ 169.3, 162.0, 150.6, 148.6, 144.4, 136.2, 135.9, 133.3, 132.5, 131.5, 130.1, 129.5, 128.3, 126.5, 126.2, 119.4, 117.7, 19.3; IR (film) ν_{max}: 3375, 3068, 3014, 2917, 1569, 1524, 1345, 1274, 1175 cm^{-1}; HRMS calcd for $[C_{23}H_{21}N_2O_3]^+$ (M+H$^+$): 373.1547; found: 373.1547.

(*E*)-*N*-{(*E*)-3-{4-[(*tert*-Butyldimethylsilyl)oxy]phenyl}-1-(2-nitrophenyl)allylidene}-2,6-dimethylaniline (2ac)

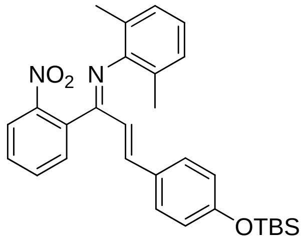

Following the general procedure **I**, the reaction of amide **1f** (135 mg, 0.5 mmol) with *tert*-butyldimethyl(4-vinylphenoxy)silane (141 mg, 0.6 mmol) at 0 °C gave, after FC (eluent: EtOAc/*n*-hexane = 1: 5), α,β-unsaturated ketimine **2ac** (217 mg, yield: 89%). When the reaction temperature is 40 °C gave α,β-unsaturated imine **2ab** (168 mg, 90%). **2ac**: Yellow solid; Mp 101-103 °C; ^{1}H NMR (500 MHz, $CDCl_3$) δ 8.12-8.19 (m, 1H), 7.75-7.82 (m, 1H), 7.69-7.74 (m, 1H), 7.61-7.68 (m, 1H), 7.04-7.11 (m, 4H), 6.94-7.01 (m, 1H), 6.67-6.73 (m, 2H), 6.52 (d, J = 16.3 Hz, 1H), 6.44 (d, J = 16.3 Hz, 1H), 2.20 (s, 6H), 0.94 (s, 9H), 0.15 (s, 6H); ^{13}C NMR (125 MHz, $CDCl_3$) δ 165.1, 157.4, 148.2, 147.3, 139.9, 135.0, 133.7, 131.4, 129.8, 129.3, 128.3, 128.0, 126.7, 124.4, 123.5, 120.4, 119.0, 25.6, 18.2, 18.2, -4.5; IR (film) ν_{max}: 3071, 3033, 2956, 2924, 2860, 1601, 1527, 1502, 1268, 909 cm^{-1}; HRMS calcd for $[C_{29}H_{35}N_2O_3Si]^+$ (M+H$^+$): 487.2411; found: 487.2411.

(*E*)-2,6-Dimethyl-*N*-[(*E*)-1-(2-nitrophenyl)-3-(4-vinylphenyl)allylidene]aniline (2ad)

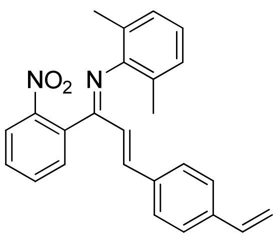

Following the general procedure **I**, the reaction of amide **1f** (135 mg, 0.5 mmol) with 1,4-divinylbenzene (78 mg, 0.6 mmol) gave, after FC (eluent: EtOAc/*n*-hexane = 1: 5), α,β-unsaturated ketimine **2ad** (167 mg, yield: 87%). Yellow solid; Mp 76-78 °C; ^{1}H NMR (500 MHz, $CDCl_3$) δ 8.14-8.20 (m, 1H), 7.77-7.83 (m, 1H), 7.71-7.76 (m, 1H), 7.63-7.69 (m, 1H), 7.25-7.30 (m, 2H), 7.12-7.17 (m, 2H), 7.07-7.11 (m, 2H), 6.96-7.02 (m, 1H), 6.58-6.68 (m, 2H), 6.48 (d, J = 16.4 Hz, 1H), 5.72 (d, J = 17.6 Hz, 1H), 5.25 (d, J = 10.8 Hz, 1H), 2.20 (s, 6H); ^{13}C NMR (125 MHz, $CDCl_3$) δ 164.9, 148.1, 147.2, 139.5, 139.0, 136.0, 134.8, 134.4, 133.8, 131.4, 129.9, 128.0, 128.0, 126.8, 126.5, 124.5, 123.6, 120.8, 115.1, 18.2; IR (film) ν_{max}: 3062, 3039, 2917, 2850, 1623, 1585, 1527, 1348, 1310, 1191, 762 cm^{-1}; HRMS calcd for $[C_{25}H_{23}N_2O_2]^+$ (M+H$^+$): 383.1754; found: 383.1754.

(*N,N'E,N,N'E*)-*N,N'*-[(*2E,2'E*)-1,4-Phenylenebis(1-(2-nitrophenyl)prop-2-en-3-yl-1-ylidene])bis(2,6-dimethylaniline) (2ae)

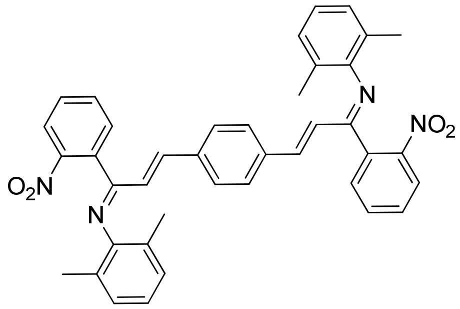

Following the general procedure **I**, the reaction of amide **1f** (338 mg, 1.25 mmol, 2.5 equiv) with 1,4-divinylbenzene (65 mg, 0.5 mmol, 1.0 equiv) gave, after FC (eluent: EtOAc/*n*-hexane = 1: 5), α,β-unsaturated ketimine **2ae** (269 mg, yield: 85%). Yellow solid; Mp 196-197 °C; ^{1}H NMR (500 MHz, $CDCl_3$) δ 8.13-8.17 (m, 2H), 7.76-7.82 (m, 2H), 7.63-7.72 (m, 4H), 7.03-7.08 (m, 8H), 6.94-6.99 (m, 2H), 6.59 (d, J = 16.4 Hz, 2H), 6.40 (d, J = 16.4 Hz, 2H), 2.16 (s, 12H); ^{13}C NMR (125 MHz, $CDCl_3$) δ 164.8, 148.1, 147.1, 138.6, 136.4, 134.6, 133.9, 131.5, 130.1, 128.1, 128.0, 126.6, 124.6, 123.8, 121.8, 18.2; IR (film) ν_{max}: 3068, 3033, 2914, 2847, 1623, 1588, 1527, 1345, 1194 cm^{-1}; HRMS calcd for $[C_{40}H_{35}N_4O_4]^+$ (M+H$^+$): 635.2653; found: 635.2652.

(*E*)-Chalcone (3a)[5]

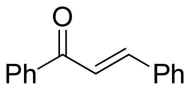

Following the general procedure **II**, the reaction of amide **1a** (113 mg, 0.5 mmol) with styrene (69 μL, 0.6 mmol) gave, after FC (eluent: EtOAc/*n*-hexane = 1: 50), α,β-unsaturated ketone **3a** (99 mg, yield: 98%). White solid; Mp 56-57 °C; ^{1}H NMR (400 MHz, $CDCl_3$) δ 7.99-8.05 (m, 2H), 7.81 (d, *J* = 15.8 Hz, 1H), 7.60-7.68 (m, 2H), 7.46-7.59 (m, 4H), 7.37-7.44 (m, 3H); ^{13}C NMR (100 MHz, $CDCl_3$) δ 190.5, 144.8, 138.2, 134.8, 132.7, 130.5, 128.9, 128.6, 128.5, 128.4, 122.0; IR (film) ν_{max}: 3059, 3025, 1665, 1644, 1599, 1574, 1491, 1445, 1341, 1213, 744, 690 cm^{-1}; MS (ESI) *m/z* 231 (M+Na$^+$, 100%).

(1*H*-Inden-2-yl)(phenyl)methanone (3b)[6]

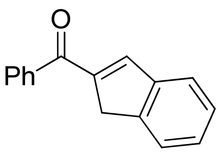

Following the general procedure **II**, the reaction of amide **1a** (113 mg, 0.5 mmol) with styrene (70 μL, 0.6 mmol) gave, after FC (eluent: EtOAc/*n*-hexane = 1: 50), α,β-unsaturated ketone **3b** (108 mg, yield: 98%). White solid; Mp 70-72 °C; ^{1}H NMR (400 MHz, $CDCl_3$) δ 7.81-7.85 (m, 2H), 7.46-7.59 (m, 6H), 7.32-7.39 (m, 2H), 3.88 (s, 2H); ^{13}C NMR (100 MHz, $CDCl_3$) δ 192.9, 145.1, 145.0, 143.6, 143.0, 138.9, 131.8, 128.8, 128.3, 128.1, 127.0, 124.5, 123.9, 38.5; IR (film) ν_{max}: 3063, 3017, 2951, 2918, 2855, 1632, 1549, 1354, 1250, 1213, 1118, 753, 703 cm^{-1}; MS (ESI) *m/z* 243 (M+Na$^+$, 100%);

(*E*)-3-(4-Bromophenyl)-1-phenylprop-2-en-1-one (3c)[5]

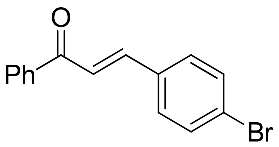

Following the general procedure **II**, the reaction of amide **1a** (113 mg, 0.5 mmol) with 4-bromostyrene (79 μL, 0.6 mmol) gave, after FC (eluent: EtOAc/*n*-hexane = 1: 50), α,β-unsaturated ketone **3c** (140 mg, yield: 98%). White solid; Mp 127-128 °C; ^{1}H NMR (400 MHz, $CDCl_3$) δ 7.98-8.04 (m, 2H), 7.74 (d, *J* = 15.7 Hz, 1H), 7.47-7.62 (m, 8H); ^{13}C NMR (100 MHz, $CDCl_3$) δ 190.2, 143.3, 138.0, 133.8, 132.9, 132.2, 129.7, 128.6, 128.5, 124.8, 122.5; IR (film)

ν_{max}: 3054, 3021, 2913, 2847 ,1657, 1603, 1582, 977, 823, 773, 686 cm^{-1}; MS (ESI) *m/z* 287, 289 ($M+H^+$, 100%).

(*E*)-3-(4-Fluorophenyl)-1-phenylprop-2-en-1-one (3d)[5]

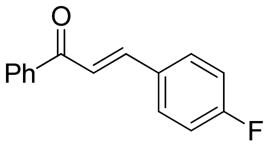

Following the general procedure **II**, the reaction of amide **1a** (113 mg, 0.5 mmol) with 4-fluorostyrene (72 μL, 0.6 mmol) gave, after FC (eluent: EtOAc/*n*-hexane = 1: 50), α,β-unsaturated ketone **3d** (98 mg, yield: 87%). White solid; Mp 88-89 °C; ^{1}H NMR (400 MHz, $CDCl_3$) δ 7.99-8.04 (m, 2H), 7.78 (d, *J* = 15.8 Hz, 1H), 7.56-7.66 (m, 3H), 7.48-7.53 (m, 2H), 7.45 (d, *J* = 15.8 Hz, 1H), 7.07-7.14 (m, 2H); ^{13}C NMR (100 MHz, $CDCl_3$) δ 190.3, 164.0 (d, *J* = 252.0 Hz), 143.5, 138.1, 132.8, 131.1 (d, *J* = 3.4 Hz), 130.3 (d, *J* = 8.8 Hz), 128.6, 128.4, 121.8, 116.1 (d, *J* = 22.0 Hz); IR (film) ν_{max}: 3062, 2913, 2839, 1669, 1607, 1586, 1567, 1217, 839, 773, 686 cm^{-1}; MS (ESI) *m/z* 249 ($M+Na^+$, 100%).

(*E*)-3-(4-Methoxyphenyl)-1-phenylprop-2-en-1-one (3e)[5]

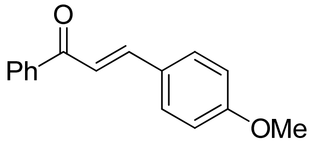

Following the general procedure **II**, the reaction of amide **1a** (113 mg, 0.5 mmol) with 4-methoxystyrene (81 μL, 0.6 mmol) gave, after FC (eluent: EtOAc/*n*-hexane = 1: 50), α,β-unsaturated ketone **3e** (111 mg, yield: 93%). White solid; Mp 76-77 °C; ^{1}H NMR (400 MHz, $CDCl_3$) δ 7.98-8.03 (m, 2H), 7.78 (d, *J* = 15.6 Hz, 1H), 7.53-7.62 (m, 3H), 7.46-7.52 (m, 2H), 7.41 (d, *J* = 15.6 Hz, 1H), 6.90-6.95 (m, 2H), 3.84 (s, 3H); ^{13}C NMR (100 MHz, $CDCl_3$) δ 190.5, 161.6, 144.6, 138.5, 132.5, 130.2, 128.5, 128.4, 127.6, 119.8, 114.4, 55.3; IR (film) ν_{max}: 3059, 2959, 2930, 2835, 1661, 1599, 1570, 1507, 1254, 1213, 1171, 1035, 1018, 831 cm^{-1}; MS (ESI) *m/z* 261 ($M+Na^+$, 100%).

(*E*)-1-(4-Bromophenyl)-3-phenylprop-2-en-1-one (3f)[5]

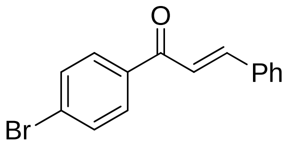

Following the general procedure **II**, the reaction of amide **1l** (152 mg, 0.5 mmol) with styrene (69

μL, 0.6 mmol) gave, after FC (eluent: EtOAc/*n*-hexane = 1: 50), α,β-unsaturated ketone **3f** (129 mg, yield: 90%). White solid; Mp 91-92 °C; ^{1}H NMR (400 MHz, $CDCl_3$) δ 7.85-7.91 (m, 2H), 7.81 (d, J = 15.7 Hz, 1H), 7.60-7.66 (m, 4H), 7.46 (d, J = 15.7 Hz, 1H), 7.38-7.44 (m, 3H); ^{13}C NMR (100 MHz, $CDCl_3$) δ 189.3, 145.3, 136.9, 134.6, 131.9, 130.7, 130.0, 129.0, 128.5, 127.8, 121.4; IR (film) ν_{max}: 3050, 2922, 1661, 1603, 1582, 1445, 985, 827, 757, 724, 694 cm^{-1}; MS (ESI) *m/z* 309, 311 (M+Na^{+}, 100%).

(*E*)-1-(4-Fluorophenyl)-3-phenylprop-2-en-1-one (3g)[5]

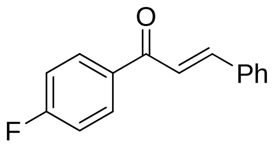

Following the general procedure **II**, the reaction of amide **1m** (122 mg, 0.5 mmol) with styrene (69 μL, 0.6 mmol) gave, after FC (eluent: EtOAc/*n*-hexane = 1: 50), α,β-unsaturated ketone **3g** (99 mg, yield: 88%). White solid; Mp 71-72°C; ^{1}H NMR (400 MHz, $CDCl_3$) δ 8.02-8.09 (m, 2H), 7.81 (d, J = 15.7 Hz, 1H), 7.61-7.67 (m, 2H), 7.50 (d, J = 15.7 Hz, 1H), 7.39-7.45 (m, 3H), 7.14-7.21 (m, 2H); ^{13}C NMR (100 MHz, $CDCl_3$) δ 188.8, 165.6 (d, J = 254.5 Hz), 145.0, 134.8, 134.5 (d, J = 3.2 Hz), 131.1 (d, J = 9.4 Hz), 130.6, 129.0, 128.4, 121.6, 115.7 (d, J = 21.9 Hz); IR (film) ν_{max}: 3063, 3034, 2926, 1669, 1611, 1582, 989, 839, 765, 690 cm^{-1}; MS (ESI) *m/z* 249 (M+Na^{+}, 100%).

(*E*)-3-Phenyl-1-(4-(trifluoromethyl)phenyl)prop-2-en-1-one (3h)[5]

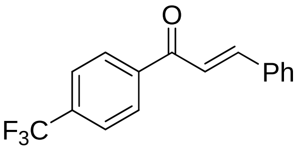

Following the general procedure **II**, the reaction of amide **1a** (147 mg, 0.5 mmol) with styrene (69 μL, 0.6 mmol) gave, after FC (eluent: EtOAc/*n*-hexane = 1: 50), α,β-unsaturated ketone **3h** (127 mg, yield: 92%). White solid; Mp 114-115 °C; ^{1}H NMR (400 MHz, $CDCl_3$) δ 8.06-8.12 (m, 2H), 7.82 (d, J = 15.7 Hz, 1H), 7.72-7.78 (m, 2H), 7.60-7.68 (m, 2H), 7.49 (d, J = 15.7 Hz, 1H), 7.39-7.45 (m, 3H); ^{13}C NMR (100 MHz, $CDCl_3$) δ 189.5, 146.0, 141.0, 134.4, 133.9 (d, J = 32.6 Hz), 130.9, 129.0, 128.7, 128.5, 125.6 (q, J = 3.6Hz), 123.6 (d, J = 272.6 Hz), 121.5; IR (film) ν_{max}: 3063, 1669, 1599, 1329, 1167, 1126, 981, 840, 769 cm^{-1}; MS (ESI) *m/z* 299 (M+Na^{+}, 100%).

(*E*)-4-Phenylbut-3-en-2-one (3i)[5]

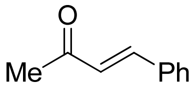

Following the general procedure **II**, the reaction of amide **1o** (82 mg, 0.5 mmol) with styrene (69 μL, 0.6 mmol) gave, after FC (eluent: EtOAc/*n*-hexane = 1: 50), α,β-unsaturated ketone **3i** (70 mg, yield: 96%). Colorless oil; ^{1}H NMR (400 MHz, $CDCl_3$) δ 7.52-7.57 (m, 3H), 7.38-7.42 (m, 3H), 6.72 (d, *J* = 16.3 Hz, 1H), 2.39 (s, 3H); ^{13}C NMR (100 MHz, $CDCl_3$) δ 198.3, 143.4, 134.4, 130.5, 128.9, 128.2, 127.2, 27.5; IR (film) ν_{max}: 3063, 3029, 2926, 2851, 1694, 1673, 1611, 1449, 1354, 1254, 976, 752, 690 cm^{-1}; MS (ESI) *m/z* 169 ($M+Na^+$, 100%).

(*E*)-1,5-Diphenylpent-1-en-3-one (3j)[7]

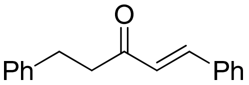

Following the general procedure **II**, the reaction of amide **1p** (127 mg, 0.5 mmol) with styrene (69 μL, 0.6 mmol) gave, after FC (eluent: EtOAc/*n*-hexane = 1: 50), α,β-unsaturated ketone **3j** (114 mg, yield: 97%). White solid; Mp 49-50 °C; ^{1}H NMR (500 MHz, $CDCl_3$) δ 7.49-7.56 (m, 3H), 7.38-7.41 (m, 3H), 7.18-7.32 (m, 5H), 6.73 (d, *J* = 16.2 Hz, 1H), 3.01 (s, 4H); ^{13}C NMR (125 MHz, $CDCl_3$) δ 199.3, 142.7, 141.2, 134.4, 130.4, 128.9, 128.5, 128.4, 128.2, 126.1, 126.1, 42.4, 30.1; IR (film) ν_{max}: 3083, 3059, 3021, 2926, 2851, 1690, 1660, 1599, 1578, 1495, 1445, 1176, 1097, 981, 748, 690 cm^{-1}; MS (ESI) *m/z* 259 ($M+Na^+$, 100%).

(*E*)-1-[(3*r*,5*r*,7*r*)-Adamantan-1-yl]-3-phenylprop-2-en-1-one (3k)[8]

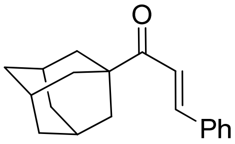

Following the general procedure **II**, the reaction of amide **1q** (142 mg, 0.5 mmol) with styrene (69 μL, 0.6 mmol) gave, after FC (eluent: EtOAc/*n*-hexane = 1: 50), α,β-unsaturated ketone **3k** (132 mg, yield: 99%). White wax; ^{1}H NMR (500 MHz, $CDCl_3$) δ 7.67 (d, *J* = 15.6 Hz, 1H), 7.55-7.60 (m, 2H), 7.36-7.41 (m, 3H), 7.16 (d, *J* = 15.6 Hz, 1H), 2.07-2.12 (m, 3H), 1.87-1.92 (m, 6H), 1.70-1.82 (m, 6H); ^{13}C NMR (125 MHz, $CDCl_3$) δ 203.9, 142.8, 135.1, 130.1, 128.8, 128.2, 120.3, 45.5, 38.0, 36.6, 27.9; IR (film) ν_{max}: 3067, 3025, 2905, 2855, 1677, 1603, 1487, 1453, 1325, 1159, 1022, 757, 699 cm^{-1}; HRMS calcd for $[C_{19}H_{22}NaO]^+$ ($M+Na^+$): 289.1563; found: 289.1563.

(1*E*,4*E*)-1,5-Diphenylpenta-1,4-dien-3-one (3l)[9]

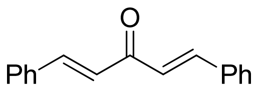

Following the general procedure **II**, the reaction of amide **1r** (126 mg, 0.5 mmol) with styrene (69 μL, 0.6 mmol) gave, after FC (eluent: EtOAc/*n*-hexane = 1: 50), α,β-unsaturated ketone **3l** (102 mg, yield: 87%). White solid; Mp 114-116 °C; ^{1}H NMR (400 MHz, $CDCl_3$) δ 7.74 (d, *J* = 16.0 Hz, 2H), 7.59-7.64 (m, 4H), 7.38-7.44 (m, 6H), 7.08 (d, *J* = 16.0 Hz, 2H); ^{13}C NMR (100 MHz, $CDCl_3$) δ 188.9, 143.3, 134.8, 130.5, 128.9, 128.4, 125.4; IR (film) ν_{max}: 3082, 3059, 2922, 2855, 1653, 1586, 985, 761, 689 cm^{-1}; HRMS calcd for $[C_{17}H_{14}NaO]^+$ ($M+Na^+$): 257.0937; found: 257.0937.

(*E*)-Methyl 5-*oxo*-7-phenylhept-6-enoate (3m)[10]

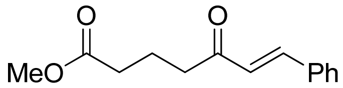

Following the general procedure **II**, the reaction of amide **1s** (125 mg, 0.5 mmol) with styrene (69 μL, 0.6 mmol) gave, after FC (eluent: EtOAc/*n*-hexane = 1: 5), α,β-unsaturated ketone **3m** (96 mg, yield: 83%). White wax; ^{1}H NMR (400 MHz, $CDCl_3$) δ 7.52-7.59 (m, 3H), 7.37-7.42 (m, 3H), 6.73 (d, *J* = 16.2 Hz, 1H), 3.68 (s, 3H), 2.75 (t, *J* = 7.2 Hz, 2H), 2.41 (t, *J* = 7.2 Hz, 2H), 2.01 (tt, *J* = 7.2, 7.2 Hz, 2H); ^{13}C NMR (100 MHz, $CDCl_3$) δ 199.4, 173.7, 142.7, 134.4, 130.5, 128.9, 128.2, 126.0, 51.5, 39.5, 33.1, 19.3; IR (film) ν_{max}: 3067, 3017, 2959, 2909, 1735, 1648, 1449, 1196, 997, 985, 765, 686 cm^{-1}; HRMS calcd for $[C_{14}H_{16}NaO_3]^+$ ($M+Na^+$): 255.0992; found: 255.0988.

(*E*)-1,5-Diphenylnon-1-en-3-one (3n)[11]

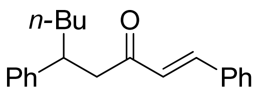

Following the general procedure **II**, the reaction of amide **1t** (110 mg, 0.5 mmol) with styrene (69 μL, 0.6 mmol) gave, after FC (eluent: EtOAc/*n*-hexane = 1: 50), α,β-unsaturated ketone **3n** (88 mg, yield: 60%). White solid; Mp 89-91 °C; ^{1}H NMR (400 MHz, $CDCl_3$) δ 7.42-7.50 (m, 3H), 7.34-7.39 (m, 3H), 7.14-7.32 (m, 5H), 6.64 (d, *J* = 16.2 Hz, 1H), 3.18-3.27 (m, 1H), 2.94 (dd, *J* = 7.0, 3.1 Hz, 2H), 1.57-1.75 (m, 2H), 1.07-1.35 (m, 4H), 0.83 (t, *J* = 7.0 Hz, 3H); ^{13}C NMR (100 MHz, $CDCl_3$) δ 199.2, 144.8, 142.4, 134.5, 130.3, 128.9, 128.4, 128.2, 127.5, 126.5, 126.2, 48.4, 41.6, 36.0, 29.6, 22.6, 13.9; IR (film) ν_{max}: 3065, 3030, 2962, 2924, 2847, 1640, 973, 762, 698 cm^{-1}; MS (ESI) *m/z* 315 ($M+Na^+$, 100%).

(*E*)-1-(2-Butylphenyl)-3-phenylprop-2-en-1-one (3o)

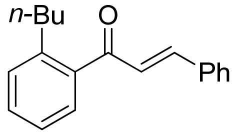

Following the general procedure **II**, the reaction of amide **1u** (96 mg, 0.5 mmol) with styrene (69 μL, 0.6 mmol) gave, after FC (eluent: EtOAc/*n*-hexane = 1: 50), α,β-unsaturated ketone **3o** (91 mg, yield: 69%). Colorless oil; ^{1}H NMR (500 MHz, $CDCl_3$) δ 7.52-7.58 (m, 2H), 7.38-7.44 (m, 6H), 7.24-7.32 (m, 2H), 7.11 (d, *J* = 16.1 Hz, 1H), 2.75 (t, *J* = 7.6 Hz, 2H), 1.54-1.61 (m, 2H), 1.29-1.37 (m, 2H), 0.88 (t, *J* = 7.6 Hz, 3H); ^{13}C NMR (125 MHz, $CDCl_3$) δ 196.2, 145.1, 140.7, 138.0, 133.6, 129.6, 129.3, 129.2, 128.0, 127.4, 126.9, 126.2, 124.4, 32.9, 32.0, 21.6, 12.9; IR (film) ν_{max}: 3062, 3020, 2953, 2927, 2860, 1662, 1598, 1454, 1332, 756 cm^{-1}; HRMS calcd for $[C_{19}H_{20}NaO]^+$ (M+Na$^+$): 287.1406; found: 287.1406.

(*E*)-1-(2-Iodophenyl)-3-phenylprop-2-en-1-one (3p)[12]

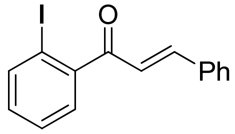

Following the general procedure **II**, the reaction of amide **1v** (131 mg, 0.5 mmol) with styrene (69 μL, 0.6 mmol) gave, after FC (eluent: EtOAc/*n*-hexane = 1: 20), α,β-unsaturated ketone **3p** (122 mg, yield: 73%). Colorless oil; ^{1}H NMR (500 MHz, $CDCl_3$) δ 7.91-7.96 (m, 1H), 7.53-7.59 (m, 2H), 7.36-7.48 (m, 6H), 7.14-7.19 (m, 1H), 7.08 (d, *J* = 16.2 Hz, 1H); ^{13}C NMR (125 MHz, $CDCl_3$) δ 196.1, 147.0, 144.6, 140.0, 134.4, 131.3, 130.9, 129.0, 128.6, 128.5, 128.0, 125.6, 92.1; IR (film) ν_{max}: 3055, 3019, 2914, 1649, 1598, 1332, 1286, 1204, 755 cm^{-1}; MS (ESI) *m/z* 357 (M+Na$^+$, 100%).

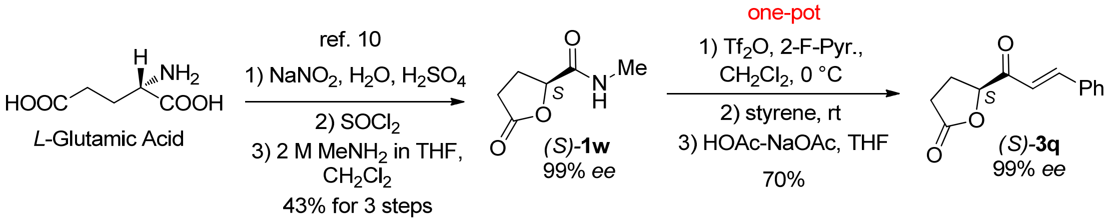

Supplementary Figure 59. Synthesis of a Chiral Building Block Enone 3q

(*S*)-5-Cinnamoyldihydrofuran-2(3*H*)-one (3q)

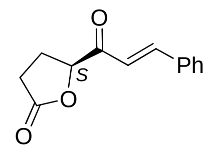

Following the general procedure **II**, the reaction of amide **1w**[13] (72 mg, 0.5 mmol) with styrene (69 μL, 0.6 mmol) (hydrolysis conditions: 5 mL THF and 5 mL of an aqueous solution of HOAc-NaOAc, the resulting mixture was stired for 0.5 h.) gave, after FC (eluent: EtOAc/*n*-hexane = 1: 2), α,β-unsaturated ketone **3q** (76 mg, yield: 70%, 99% *ee*). White solid; Mp 65-67 °C; $[\alpha]_D^{20}$ = +51.5 (*c* = 1.0, $CHCl_3$); ^{1}H NMR (400 MHz, $CDCl_3$) δ 7.78 (d, *J* = 16.0 Hz, 1H), 7.56-7.65 (m, 2H), 7.37-7.43 (m, 3H), 7.06 (d, *J* = 16.0 Hz, 1H), 5.06-5.14 (m, 1H), 2.51-2.63 (m, 3H), 2.32-2.45 (m, 1H); ^{13}C NMR (100 MHz, $CDCl_3$) δ 195.4, 176.2, 146.2, 133.9, 131.3, 129.0, 128.8, 119.9, 81.1, 27.3, 25.0; IR (film) ν_{max}: 3062, 3020, 2933, 2850, 1787, 1691, 1613, 1454, 1169, 1044, 707 cm^{-1}; HRMS calcd for $[C_{13}H_{12}NaO_3]^+$ (M+Na$^+$): 239.0679; found: 239.0677; Conditions for the chiral HPLC analysis: Chiralpak AD-H (*n*-hexane/isopropanol, 50: 50), flow rate = 1.0 mL·min^{-1}, R_t = 5.72, 6.45 min, respectively. The enantiomeric excess was determined to be 99%.

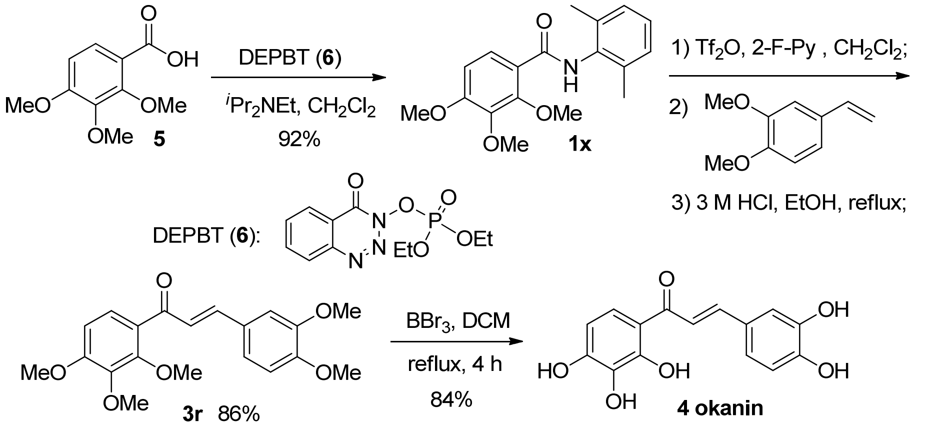

Supplementary Figure 60. Short synthesis of Okanin (4)

***N*-(2,6-Dimethylphenyl)-2,3,4-trimethoxybenzamide (1x)**

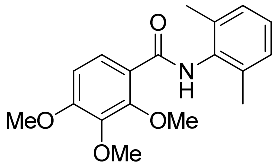

To a solution of 2,3,4-trimethoxybenzoic acid **5** (212 mg, 1.0 mmol, 1.0 equiv) and DEPBT (**6**) (498 mg, 2.0 mmol, 2.0 equiv) in CH_2Cl_2 (5 mL, 0.2 M), after being cooled to 0 °C, ^{i}Pr$_2$NEt (350 μL, 2.0 mmol, 2.0 equiv) was added, and the reaction mixture was stirred at 0 °C for 10 min,

2,6-dimethylaniline (135 μL, 1.1 mmol, 1.1 equiv) was added, then the mixture was allowed to warm-up to room temperature and stirred for 2 h. Saturated NaCl solution was added, and extracted with CH_2Cl_2 (3 × 10 mL). The combined organic layer was washed with brine and dried over anhydrous Na_2SO_4, filtered, and concentrated under reduced pressure. The residue was purified by flash column chromatography on silica gel to give amide **1x** (290 mg, yield: 92%). White solid; Mp 108-109 °C; ^{1}H NMR (400 MHz, $CDCl_3$) δ 9.24 (br s, 1H), 8.00 (d, *J* = 8.9 Hz, 1H), 7.09-7.16 (m, 3H), 6.83 (d, *J* = 8.9 Hz, 1H), 4.10 (s, 3H), 3.94 (s, 3H), 3.93 (s, 3H), 2.30 (s, 6H); ^{13}C NMR (100 MHz, $CDCl_3$) δ 163.1, 156.7, 152.4, 141.7, 135.4, 134.5, 128.1, 127.2, 127.0, 118.6, 107.6, 61.9, 61.0, 56.1, 18.7; IR (film) ν_{max}: 3350, 2933, 2841, 1668, 1591, 486, 1457, 1294, 1092 cm^{-1}; HRMS calcd for $[C_{18}H_{21}NNaO_4]^+$ $(M+Na^+)$: 338.1363; found: 338.1364.

(*E*)-3-(3,4-Dimethoxyphenyl)-1-(2,3,4-trimethoxyphenyl)prop-2-en-1-one (3r)

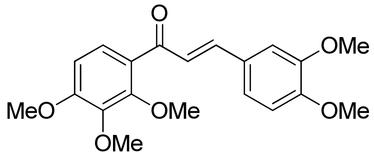

Following the general procedure **II**, the reaction of amide **1x** (157 mg, 0.5 mmol) with 1,2-dimethoxy-4-vinylbenzene (99 mg, 0.6 mmol) gave, after FC (eluent: EtOAc/*n*-hexane = 1: 3), α,β-unsaturated ketone **3r** (154 mg, yield: 86%). Colorless oil; ^{1}H NMR (400 MHz, $CDCl_3$) δ 7.62 (d, *J* = 15.8 Hz, 1H), 7.46 (d, *J* = 8.4 Hz, 1H), 7.34 (d, *J* = 15.8 Hz, 1H), 7.20 (dd, *J* = 8.4, 1.9 Hz, 1H), 7.14 (d, *J* = 1.9 Hz, 1H), 6.89 (d, *J* = 8.4 Hz, 1H), 6.76 (d, *J* = 8.4 Hz, 1H), 3.91-3.94 (m, 15H); ^{13}C NMR (100 MHz, $CDCl_3$) δ 191.0, 156.7, 153.5, 151.2, 149.2, 143.4, 142.1, 128.1, 127.0, 125.5, 124.6, 122.8, 111.1, 110.2, 107.3, 62.1, 61.0, 56.1, 55.9, 55.9; IR (film) ν_{max}: 3055, 2998, 2936, 2841, 1665, 1585, 1518, 1457, 1287, 1258, 1098, 1021 cm^{-1}; HRMS calcd for $[C_{20}H_{22}NaO_6]^+$ $(M+Na^+)$: 381.1309; found: 381.1312.

Okanin (4)

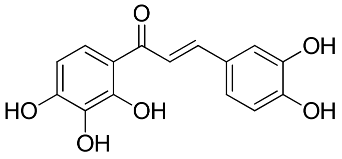

To a previously cooled solution (0 °C) of methoxy substituted chalcone **3r** (358 mg, 1.0 mmol) in dried CH_2Cl_2 (20 mL), a 1 M solution of BBr_3 in DCM (12 mL, 12 equiv) was slowly added under Ar atmosphere. After stirring 0.5 h at room temperature under Ar, the reaction was refluxed and

stirred for 4 h. Then cooled to rt, H_2O was added to the reaction mixture. A solid precipitate was formed, it was separated by filtration and washed with H_2O and CH_2Cl_2 to give amide **okanin** (**4**) (242 mg, yield: 84%). Yellow amorphous powder; ^1H NMR (500 MHz, MD_3OD) δ 7.72 (d, J = 15.4 Hz, 1H), 7.49-7.56 (m, 2H), 7.18 (s, 1H), 7.10 (d, J = 8.2 Hz, 1H), 6.82 (d, J = 8.2 Hz, 1H), 6.47 (d, J = 8.8 Hz, 1H); ^{13}C NMR (125 MHz, MD_3OD) δ 194.9, 155.3, 154.1, 150.8, 147.7, 146.8, 134.6, 129.3, 124.5, 124.1, 119.3, 117.5, 116.7, 116.0, 109.4; IR (film) ν_{max}: 3251, 2921, 2847, 1643, 1604, 1283, 1191, 1178 cm^{-1}; HRMS calcd for $[C_{15}H_{12}NaO_6]^+$ $(M+Na^+)$: 311.0526; found: 311.0526.

Supplementary References:

1. Medley, J. W. & Movassaghi, M. Direct dehydrative *N*-pyridinylation of amides. *J. Org. Chem.* **74**, 1341–1344 (2009).
2. Jochims, J. C., Hehl, S. & Herzberger, S. Preparation and Beckman rearrangement of *o*-(chiorooxalyl) oximes. *Synthesis* 1128–1133 (1990).
3. White, K. L., Mewald, M. & Movassaghi, M. Direct observation of intermediates involved in the interruption of the Bischler−Napieralski reaction. *J. Org. Chem.* **80**, 7403−7411 (2015).
4. Huang, C.-Y. (D.), Doyle, A. G. Nickel-catalyzed Negishi alkylations of styrenyl aziridines. *J. Am. Chem. Soc.* **134**, 9541−9544 (2012).
5. Jiang, Q., Jia, J., Xu, B., Zhao, A., Guo, C.-C. Iron-facilitated oxidative radical decarboxylative cross-coupling between α-oxocarboxylic acids and acrylic acids: an approach to α,β-unsaturated carbonyls. *J. Org. Chem.* **80**, 3586–3596 (2015).
6. Tu, S., Sha, Y., Xu, L., Xiao, Z., Ye, L., Fang, J. Studies on the synthesis of 2-acyl-1*H*-indenes via one-pot palladium-catalysed tandem Heck–aldol reaction. *J. Chem. Res.* **34**, 254−258 (2010).
7. Kotani, S., Osakama, K., Sugiura, M., Nakajima, M. A tertiary amine as a hydride donor: trichlorosilyl triflate-mediated conjugate reduction of unsaturated ketones. *Org. Lett.* **13**, 3968−3971 (2011).
8. Hori, K., Ando, M., Takaishi, N., Inamoto, Y. Palladium-catalyzed acylation of activated alkenes with bridgehead acid chlorides. *Tetrahedron Lett.* **28**, 5883–5886 (1987).
9. Hua, Z. G., Liu, J., Zeng, P. L., Dong, Z. B. Synthesis of α,α’-bis(substituted benzylidene)ketones catalysed by a $SOCl_2$/ EtOH reagent. *J. Chem. Res.* **28**, 55−56 (2004).
10. Inaba, S.-I., Rieke, R. D. Metallic nickel-mediated synthesis of ketones by the reaction of benzylic, allylic, vinylic, and pentafluorophenyl halides with acid halides. *J. Org. Chem.* **50**, 1373−1381 (1985).
11. Šebesta, R., Pizzuti, M. G., Minnaard, A. J., Feringa, B. L. Copper-catalyzed enantioselective conjugate addition of organometallic reagents to acyclic dienones. *Adv. Synth. Catal.* 349, 1931–1937 (2007).
12. Minatti, A., Zheng, X., Buchwald, S. L. Use of deuterium labeling studies to determine the stereochemical outcome of Palladium migrations during an asymmetric intermolecular Heck reaction. *J. Org. Chem.* **72**, 9253−9258 (2007).
13. Ruan, Y.-P., Wei, B.-G., Xu, X.-Q., Liu, G., Yu, D.-S., Liu, L.-X., Huang, P.-Q. Detailed studies on the enantioselective synthesis and HPLC enantioseparation of *N*-protected 3-hydroxyglutarimides. *Chirality* **17**, 595−599 (2005).
